# A qualitative investigation of paediatric intensive care staff attitudes towards the diagnosis of lower respiratory tract infection in the molecular diagnostics era

**DOI:** 10.1007/s44253-023-00008-z

**Published:** 2023-07-07

**Authors:** John A. Clark, Andrew Conway Morris, Constantinos Kanaris, David Inwald, Warwick Butt, Joshua Osowicki, Luregn J. Schlapbach, Martin D. Curran, Deborah White, Esther Daubney, Shruti Agrawal, Vilas Navapurkar, M. Estée Török, Stephen Baker, Nazima Pathan

**Affiliations:** 1 Department of Paediatrics, University of Cambridge, Level 8, Addenbrooke’s Hospital, Cambridge Biomedical Campus, Cambridge, CB2 0QQ UK; 2 Cambridge University Hospitals NHS Foundation Trust, Cambridge, UK; 3 Division of Anaesthesia, Department of Medicine, University of Cambridge, Cambridge, UK; 4 Division of Immunology, Department of Pathology, University of Cambridge, Cambridge, UK; 5 Blizard Institute, Queen Mary University of London, London, UK; 6 Paediatric Intensive Care Unit, Royal Children’s Hospital Melbourne, Melbourne, Australia; 7 Department of Critical Care, University of Melbourne, Melbourne, Australia; 8 Infectious Diseases Unit, Department of General Medicine, Royal Children’s Hospital Melbourne, Parkville, Australia; 9 Department of Intensive Care and Neonatology and Children’s Research Center, University Children’s Hospital Zürich, Zurich, Switzerland; 10 United Kingdom Health Security Agency, Clinical Microbiology and Public Health Laboratory, Cambridge, UK; 11 Division of Infectious Diseases, Department of Medicine, University of Cambridge, Cambridge, UK; 12 Cambridge Institute of Therapeutic Immunology and Infectious Disease, University of Cambridge, Cambridge, UK

**Keywords:** Pneumonia, Critical care, Paediatric, Diagnostic techniques, Respiratory system, Evaluations, Qualitative

## Abstract

**Background:**

In the past decade, molecular diagnostic syndromic arrays incorporating a range of bacterial and viral pathogens have been described. It is unclear how paediatric intensive care unit (PICU) staff diagnose lower respiratory tract infection (LRTI) and integrate diagnostic array results into antimicrobial decision-making.

**Methods:**

An online survey with eleven questions was distributed throughout paediatric intensive care societies in the UK, continental Europe and Australasia with a total of 755 members. Participants were asked to rate the clinical factors and investigations they used when prescribing for LRTI. Semi-structured interviews were undertaken with staff who participated in a single-centre observational study of a 52-pathogen diagnostic array.

**Results:**

Seventy-two survey responses were received; most responses were from senior doctors. Whilst diagnostic arrays were used less frequently than routine investigations (i.e. microbiological culture), they were of comparable perceived utility when making antimicrobial decisions. Prescribers reported that for arrays to be clinically impactful, they would need to deliver results within 6 h for stable patients and within 1 h for unstable patients to inform their immediate decision to prescribe antimicrobials. From 16 staff interviews, we identified that arrays were helpful for the diagnosis and screening of bacterial LRTI. Staff reported it could be challenging to interpret results in some cases due to the high sensitivity of the test. Therefore, results were considered within the context of the patient and discussed within the multidisciplinary team.

**Conclusions:**

Diagnostic arrays were considered of comparable value to microbiological investigations by PICU prescribers. Our findings support the need for further clinical and economic evaluation of diagnostic arrays in a randomised control trial.

**Trial registration:**

Clinicaltrials.gov, NCT04233268. Registered on 18 January 2020.

**Supplementary Information:**

The online version contains supplementary material available at 10.1007/s44253-023-00008-z.

## Introduction

Lower respiratory tract infection (LRTI) is a leading cause of hospitalisation and mortality in children [1, 2]. In paediatric intensive care units (PICU), broad-spectrum antimicrobial therapy is commonly prescribed for LRTI without microbiological confirmation of the causative agent [3]. A recent series of focus groups highlighted clinicians’ concerns for adverse consequences if they did not prescribe antimicrobials [4]. Although there are definitions for the diagnosis of community-acquired pneumonia (CAP) and ventilator-associated pneumonia (VAP) in children, in practice, they have poor specificity, and it is unclear how much they are used for antimicrobial decision-making [5–8].

When trialling novel diagnostics such as molecular arrays, it is critical to consider factors contributing to antimicrobial decision-making and clinician buy-in. An antimicrobial decision-making study using cytokines found that although specific biomarkers worked, this method did not change the prescribing practices of clinicians [9]. As was highlighted following the process evaluation, “considerable work needs to be done to understand these complex behavioural issues and prescribing influences, if diagnostic tests are to have a greater chance of influencing outcomes in conditions like suspected VAP” [10]. Studies of prescribing decisions related to LRTI are limited to adults with mild illness [11, 12], post hoc analysis of children presenting to emergency departments [13] and intensivists’ opinions before introduction and experience of using a diagnostic array [14, 15].

From April 2020 to January 2022, a single-centre diagnostic study of a custom TaqMan array card (TAC) was undertaken within a 13-bed general PICU at Addenbrooke’s Hospital, Cambridge, UK [16, 17]. The TAC is unique among diagnostic arrays as it is user-customisable. It outputs a cycle threshold (Ct) value for each pathogen target as an indication of the target copy number, providing a result to clinicians on a wide range of respiratory pathogens [13].

Here, we sought to identify how international PICU prescribers make treatment decisions in children with suspected LRTI and determine how local PICU staff perceived the implementation of a diagnostic array into clinical practice. This was conducted via (1) an online survey distributed by paediatric intensive care societies in the UK, continental Europe and Australasia and (2) semi-structured interviews with staff who participated in the study in Cambridge.

## Methods

### Survey study design

We undertook a cross-sectional survey with five sections and eleven questions developed to address elements of the Checklist for Reporting Results of Internet E-Surveys (CHERRIES) [18]. The survey was directed at PICU prescribers (Supplementary materials). Participants were asked to report clinical factors or investigations they considered relevant to the scenario of a patient with a suspected respiratory infection, then asked to rate these factors on a Likert-scale [19], from low importance (0) to high importance (100) via adaptive questioning.

### Study preparation

The survey was advertised via the European Society of Paediatric and Neonatal Intensive Care (ESPNIC) by the Infection Inflammation and Sepsis Section, the Paediatric Critical Care Society Study Group, UK (PCCS-SG) and the Australian and New Zealand Intensive Care Society Paediatric Study Group (ANZICS-PSG). These groups reviewed the study protocol internally and formally endorsed the project. The networks emailed potential participants the study information sheet and survey via their membership database on behalf of the research team.

### Survey administration

The study was delivered via REDCap, a secure electronic data management system hosted by the University of Cambridge [20] from November 2021 to April 2022.

### Statistical analysis

All responses to the survey were included in the analysis where the participant had completed a response to at least the first scenario. The data were described using simple descriptive statistics and proportions for binary variables. Likert scales were transformed into a scale of 0 to 100. Skewed data were reported by median and interquartile range, and normally distributed data were reported by mean and standard deviation. In two-stage questions relating to clinical factors (4b, 5b, 6a, 7b), the survey instrument asked respondents to give a rating only where they had identified the factor to be relevant. Variables not considered applicable were corrected to zero. However, in two-stage questions relating to investigations, this correction was not performed, given it is possible that the respondent did not have the investigation available at their institution. Medians were compared using the Mann–Whitney *U* test, whilst means were compared using the Student *t* test. Proportions were compared using the Fisher’s exact test. Graphs were generated with R studio v7.1, R version 4.2.0 using ggplot2 [21, 22]. Figures were created in Biorender.com.

### Interview study design

The interviews were reported according to the consolidated criteria for reporting qualitative studies (COREQ) checklist [23]. Interviews were undertaken after the completion of the TAC diagnostic study.

A semi-structured interview guide (Supplemental materials) was developed by the research team using established interview methodology [24], with input from psychology and decision-making experts. Interviews were determined to be the optimal qualitative research method, given their ability to capture ‘desired outcomes’ and enhance the ‘peripheral vision’ of the researchers, as described by *Sofaer* [25]. Following informed consent, interviews with participants were recorded and transcribed by the interviewer (Supplementary materials). Thematic analysis and coding were then undertaken in NVivo 12.7.0 [26], with themes identified on exploration of the data using an inductive approach [27].

## Results

### Survey results

#### Respondent and centre characteristics

There were 72 respondents to the survey in 44 PICUs (Table 1) located across 22 countries (Figure S1). The response rate was 72/755 (10%). Eighteen centres reported their current burden of antimicrobial use and pneumonia (Table S2). There were a total of 59 (68%) mechanically ventilated patients were receiving systemic antimicrobial therapy. Of mechanically ventilated patients, 14 (16%) had suspected CAP, and 11 (12%) had suspected VAP.

**Table 1:** Demographics of survey respondents and participating centres

Factor	Participants *N* = 72	Centres *N* = 44
	*N* (%)	*N (%)*
Location		
UK	30 (42)	11 (25)
Continental Europe	21 (29)	19 (43)
Australasia	11 (15)	5 (12)
Asia	6 (8)	5 (12)
Africa	3 (4)	3 (7)
South America	1 (1)	1 (2)
Services provided within the PICU of respondent		
General	72 (100)	44 (100)
Surgical	61 (85)	36 (82)
Neuro-intensive care	58 (81)	33 (75)
Cardiac	34 (47)	18 (41)
Combined adult ICU	5 (7)	4 (9)
Professional group		
Senior doctor	52 (72)	
Senior^a^ doctor-in-training	12 (17)	
Junior^a^ doctor-in-training	2 (3)	
Advanced nurse practitioner	5 (7)	
Nurse practitioner	1 (1)	

*PICU* Paediatric intensive care unit

^a^Doctors-in-training are described as ‘junior’ and ‘senior’ according to their completion of post-graduate examinations in a primary medical specialty

#### Clinical features contributing to the decision to prescribe antimicrobial therapy for CAP and VAP

Features in the clinical history ranked highly in importance in relation to antimicrobial commencement for CAP are shown in Table S3. The most frequently reported features used by clinicians to determine the need to treat CAP were immunosuppression of the patient (*n* = 65; 90%), history of chronic respiratory disease (*n* = 61; 85%), colonisation of the respiratory tract (*n* = 56; 78%) and known colonisation with antimicrobial resistant organisms (*n* = 52; 72%). Fewer prescribers used physical findings in their decision-making, with the highest-ranking factors being fever (*n* = 50; 69%), oxygen requirement (*n* = 42; 58%) and ventilator pressures (*n* = 29; 40%). However, the frequency of the use of these factors did not necessarily relate to their perceived importance (Figure S2a).

Physical findings were used more frequently than features on clinical history by prescribers deciding whether to treat VAP (Table S3), including an increase in ventilator requirements (*n* = 54; 86%), fever (*n* = 52; 83%) and an increase in oxygen requirement (*n* = 51; 81%). However, the factors of greatest perceived importance were whether the patient was immunosuppressed or had a generalised respiratory deterioration (Figure S2b).

#### Investigations for CAP and VAP

The most frequently requested investigations for CAP were inflammatory markers (*n* = 71; 99%), chest radiograph (*n* = 70; 97%), viral respiratory virus qPCR panel (*n* = 64; 89%) and respiratory microbiology (*n* = 60; 83%) (Table S4). Microbiology cultures were more commonly performed on ETT aspirates (ETA) (CAP (*n* = 53; 74%), VAP (*n* = 46; 73%)), than invasive methods such as bronchoscopy (CAP (*n* = 6; 8%), VAP (*n* = 8; 13%)) or mini-BAL (CAP (*n* = 16; 22%), VAP (*n* = 8; 13%)). The most frequently requested investigations for VAP were chest radiograph (requested in all cases), inflammatory markers (94%), microbiological culture (92%) and viral respiratory virus qPCR panel (75%). Several investigations were significantly more likely to be requested for CAP than VAP, including blood cultures (*p* = 0.014), respiratory viral qPCR panels via NPA (*p* = 0.037) and swabs (*p* = 0.018) and urinary pneumonia antigens (*p* = 0.017).

Excluding the two centres partaking in diagnostic array studies, 35/64 (55%) of clinicians reported they would request a multi-pathogen array for CAP, and 23/55 (42%) would request one for VAP. This would be most commonly obtained from an ETA sample.

**Fig. 1:**
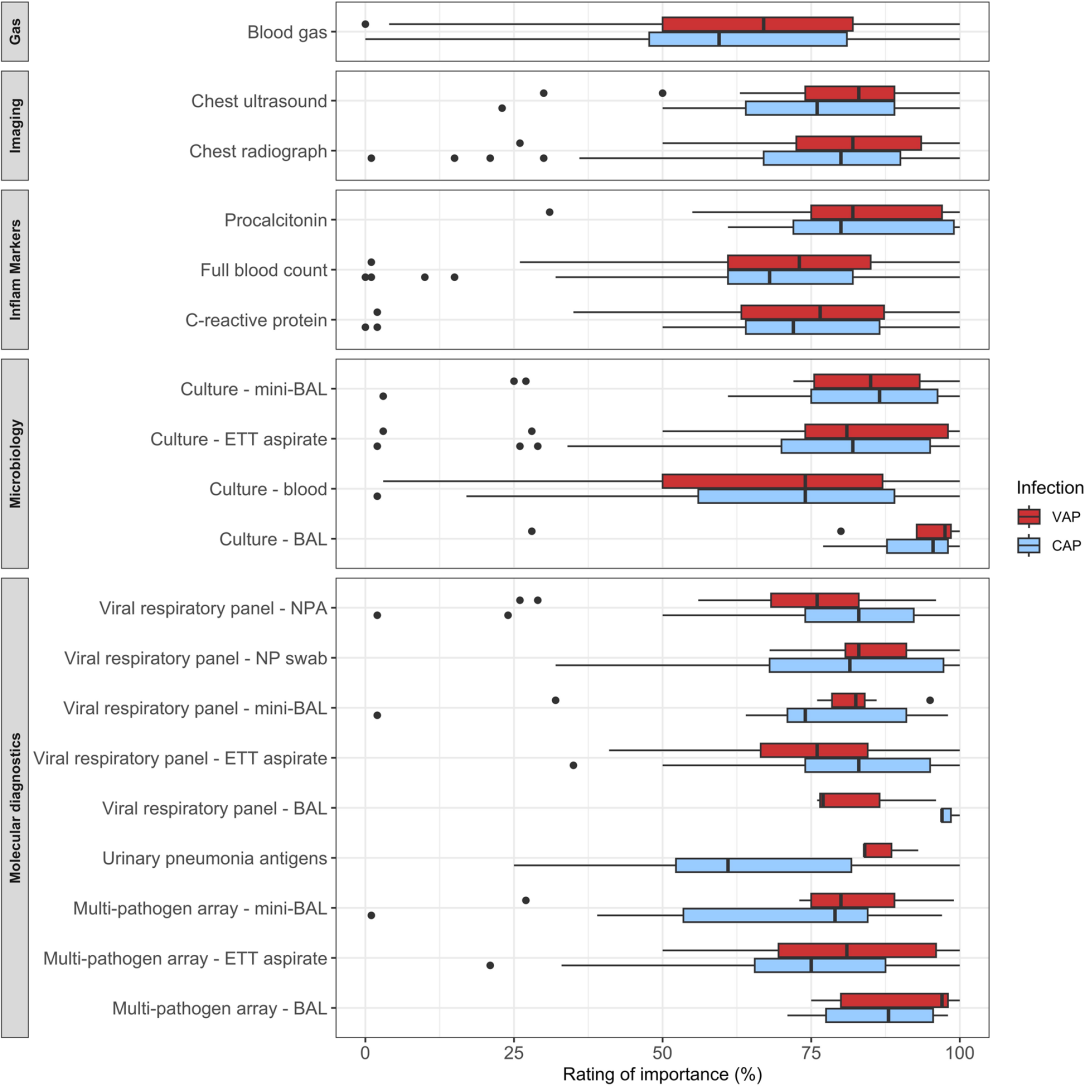
Rating of investigations used by clinicians making prescribing decisions for the treatment of pneumonia in the paediatric intensive care unit. This box and whisker plot represents the perceived relevance, according to prescribers, of investigations contributing to the decision to commence antimicrobial therapy in ventilated children with suspected respiratory infections. BAL: bronchoalveolar lavage; CAP: community-acquired pneumonia; ETT: endotracheal tube; mini-BAL: non-bronchoscopic bronchoalveolar lavage; NP: nasopharyngeal; NPA: nasopharyngeal aspirate; VAP: ventilator-associated pneumonia

The perceived usefulness of commonly requested investigations to determine antimicrobial prescription varied greatly, with broad interquartile ranges for many investigations (Fig. 1).

#### Failing treatment for LRTI

Prescribers most frequently reported an increase in ventilator requirements (*n* = 52, 88%), oxygen requirement (*n* = 45; 76%) and fever (*n* = 41; 69%) as indicators that patients may not be responding to antimicrobial therapy (Table S5). Ventilator requirements (median 79% perceived relevance, IQR 66.0–90.0) and oxygen requirements (median 75% perceived relevance, IQR 55.0–94.0) were of greatest relevance Figure S3). The most frequently requested investigations for treatment failure were inflammatory markers (*n* = 55; 93%), chest radiograph (*n* = 47; 80%) and respiratory microbiological culture (*n* = 40; 68%) (Table S6). More clinicians would request a procalcitonin (*n* = 36, 61%) for the investigation of suspected failed antimicrobial therapy than when considering antimicrobial therapy for CAP (*n* = 33; 46%) and VAP (*n* = 33; 52%). A multi-pathogen array would be requested by the 18/51 (35%) of participants not involved in diagnostic array studies. The value of such investigations was perceived comparably (Figure S4), except for the BAL samples used to undertake microbiology culture or multi-pathogen array.

#### Cessation of antimicrobial therapy

The majority of clinicians reported that they would decide to cease antimicrobial therapy based on the clinical status of the patient (92%) (Table S7). This was considered more relevant than investigations (Figure S5).

#### Confidence in the diagnosis of VAP

Clinicians reported they were 70.4% (SD 17.6%) confident in making a diagnosis of VAP. There was no significant difference in the confidence of senior doctors (72.3%, SD 18.0%) versus doctors-in-training and nurse prescribers (64.4%, SD 15.3%, *p* = 0.118).

#### Turnaround times for diagnostic arrays

Prescribers reported that if an ideal diagnostic array were available, with high sensitivity and specificity, they would be willing to wait median 6 h (IQR 4–24 h) before starting antimicrobial therapy in stable patients with suspected CAP and median 6 h (IQR 4–18 h) in patients with suspected VAP. Acceptable turnaround times were significantly shorter if the patient were unstable, accepting median 1 h (IQR 0–3 h, *p* < 0.001) for CAP and median 1 h (IQR 0–3.5 h, *p* < 0.001) for VAP.

### Staff interviews

Interviews occurred between January 2022 and April 2022, following 21 months of unit experience with the TAC diagnostic array and the completion of patient recruitment for the diagnostic study. The median interview duration was 13 min (IQR 12–17 min). The total interview time was 2 h 35 min. Interviewing continued until thematic saturation was reached. Of all PICU staff, 5/8 (63%) senior doctors, 6/12 (50%) doctors-in-training and 5/60 (8%) nurses participated in interviews. One senior doctor was excluded as the chief investigator of the study.

Here we describe areas of exploration in the interviews and themes identified within these topics. Supporting quotations for these themes are presented.

#### Staff perception of benefits and challenges of using TAC

Integration of TAC into clinical practice was felt to be an exciting development that could assist with decision-making (Table S8, Quotes 1–2). There was no situation in which staff described reversing decisions made based on TAC results once routine investigation results became available (Table S8, Quote 3). TAC was requested proactively and was felt to have become a standard component of care in the PICU (Table S8, Quotes 4–7). Routine microbiological culture continued to be ordered during the study; however, there was a reduction in routine viral NPA tests (Table S8, Quotes 8–9). This reduction was due to reduced viral multiplex qPCR test availability due to laboratory pressures during the SARS-CoV-2 pandemic.

TAC was highly sensitive given it is a qPCR test and sometimes identified microorganisms that may have been commensals or pathobionts (commensals that can become pathogenic). Staff found these additional detections challenging to interpret at times (Table S8, Quotes 10–18), particularly those with a borderline Ct values (Table S8, Quote 19). Due to the high sensitivity of TAC, there were occasions in which unexpected infectious airborne pathogens were detected. These results had an impact on senior nursing staff, who had to consider the best use of single rooms and infection control within the PICU (Table S8, Quote 20).

#### Situations in which a TAC was requested

**Fig. 2:**
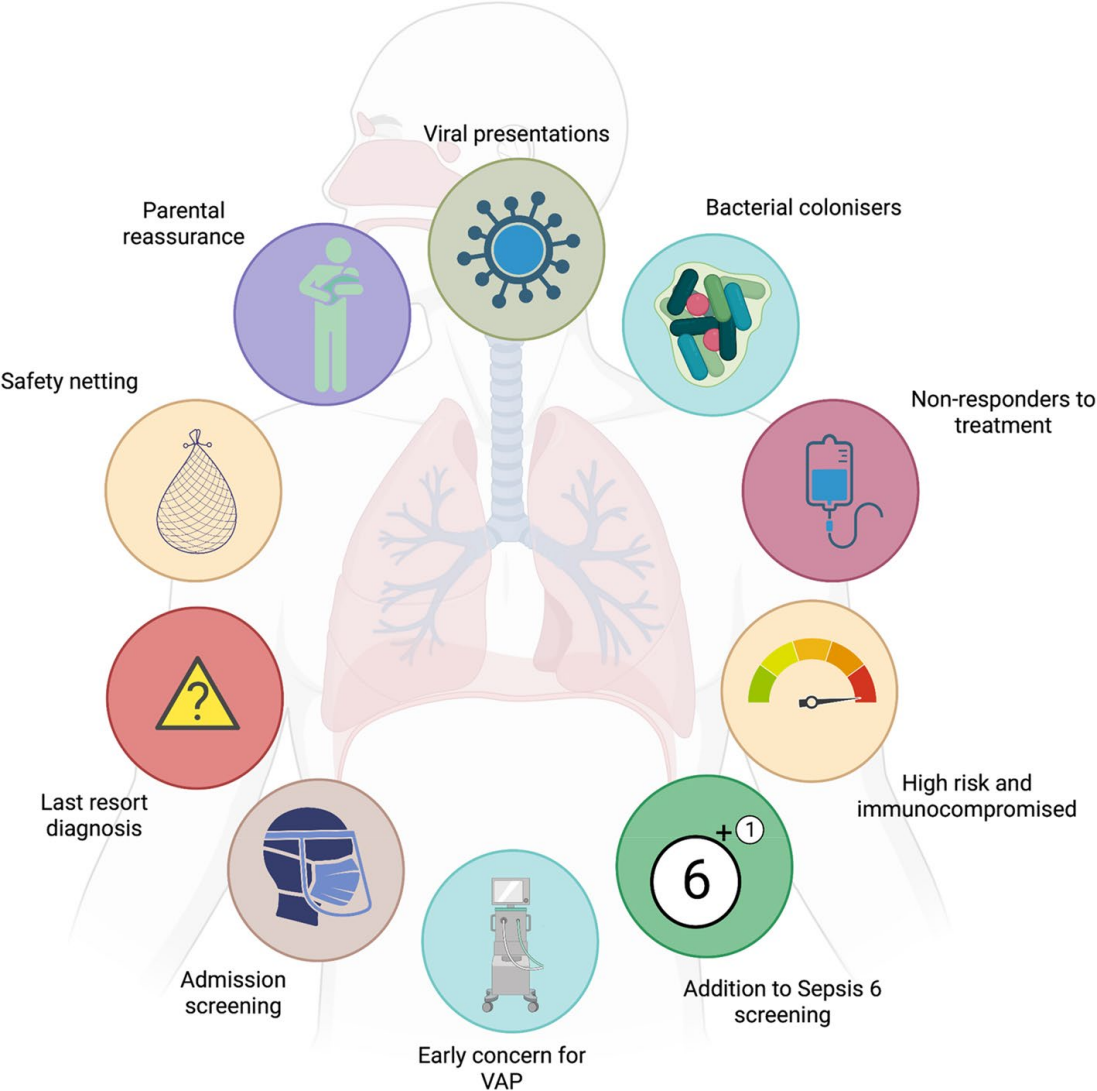
Situations in which custom TaqMan array card was considered helpful according to paediatric critical care staff. Paediatric critical care staff were asked why they requested a custom 52-respiratory pathogen diagnostic TaqMan array card (TAC) within the bounds of an observational study. They reported that the test was used for common presentations to the paediatric intensive care unit (PICU), which were generally viral in nature, but the test also helped identify bacterial organisms in children known to have respiratory colonisation. Some children received the test as they were not responding to antimicrobial therapy, or they at high risk of opportunistic infection. There were suggestions that TAC could be used as a screening tool, as an additional element to screening in sepsis (the Sepsis 6) and ventilator-associated pneumonia (VAP), and for cohorting patients on admission to the PICU. Given the array covered a broad range of targets, the staff found it reassuring that it meant there was a ‘safety net’ in place to avoid missing severe bacterial infection. There were some situations in which TAC was requested when a source of infection could not be identified in seriously unwell children. Being able to report results earlier than standard diagnostic tests was reassuring for parents in understanding the cause of their child’s illness

Within the bounds of the study, there were a range of reasons staff utilised TAC (Fig. 2), with supporting quotations presented in the supplementary materials (Table S9).

#### Interpretation of TAC by PICU staff

**Fig. 3:**
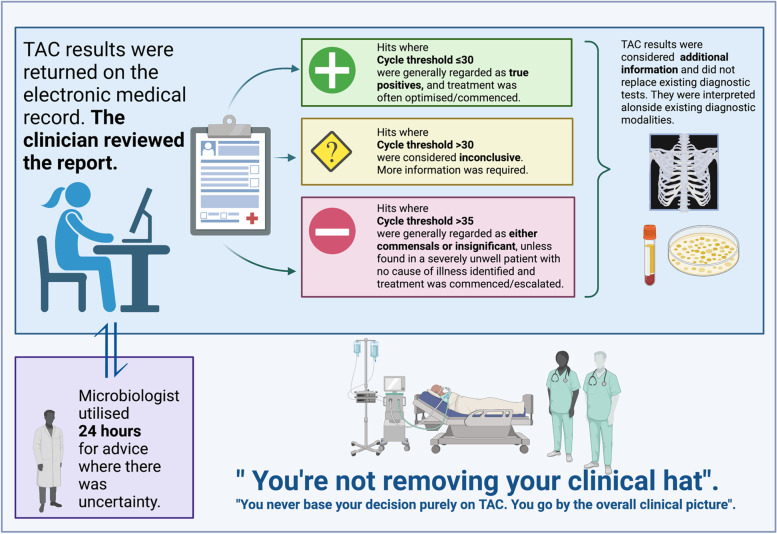
Approach taken by paediatric critical care staff in interpreting TaqMan array card results. This figure highlights how clinicians interpreted a custom 52-pathogen TaqMan array card (TAC) for the diagnosis of suspected respiratory infection. Results were reported according to Cycle threshold (Ct), which represents the number of cycles of PCR amplification that occurred before an exponential increase of the fluorescent reporter signal. Clinicians considered patient-related factors and engaged with the microbiology team in situations of uncertainty

TAC was used as an adjunct to existing investigations for respiratory infection (Table S10, Quotes 1–4) and was taken in the context of the clinical history and status of the patient (Table S10, Quotes 5–9). There was a misunderstanding among a minority of staff regarding the scope of TAC and other existing molecular tests (Table S10, Quotes 10–13). Some were confused regarding the direction in which a Ct value indicates the positivity of the test (Table S10, Quotes 14–15). The reporting of Ct values gave staff confidence regarding the relevance of detections (Table S10, Quotes 16–21). There was some variation in the thresholds at which clinicians perceived significance (Fig. 3). Staff reported that their confidence in the interpretation of TAC increased throughout the study (Table S10, Quotes 22–24). Doctors had a low threshold to discuss results within the multidisciplinary team (Table S10, Quotes 25–27), which helped in situations of uncertainty.

#### Actions of PICU staff after TAC results became available

TAC was used by prescribers in their decision to commence and cease antimicrobial therapy as well as tailor the spectrum of antimicrobial cover (Table 2, Quotes 1–9). If clinicians had a high degree of suspicion of bacterial infection despite a negative TAC result, they would continue antimicrobial therapy (Table 2, Quote 10). If Ct values were borderline, clinicians would await further information prior to changing treatment (Table 2, Quote 11) unless the patient was severely unwell (Table 2, Quote 12). Nursing staff reported that TAC results had implications for their practice in terms of making infection control decisions (Table 2, Quotes 13–14) but also used it to assist their interpretation of patient physiology (Table 2, Quote 15). Some patients required additional immunological investigations when TAC yielded unusual organisms (Table 2, Quotes 16–17).

**Table 2:** Actions following TaqMan array card result—quotations supporting thematic analysis

Theme	Supporting quotations
Prescribers used TAC results in their decision to stop, start and change antimicrobial prescriptions. Understanding the overall impact of TAC on antimicrobial prescriptions is complex due to the severity and heterogeneity of PICU patients	1. “…the investigation helped us to titrate antibiotic therapy properly, so we were treating the bug causing the problem. Or, if there were no bugs, to stop antibiotics in a timely manner”.—C1
	2. “…it would affect narrowing down the spectrum of antibiotics if we had a result earlier that suggested we could use something narrow spectrum”.—C5
	3. “I would like it in most respiratory cases as early as possible… these patients come on a broad-spectrum cephalosporin plus or minus sometimes clarithromycin if they’re suspecting atypical. Allowing us to stop those earlier is a long-term gain really in reducing, in our region, resistance to those antibiotics. It’s obviously beneficial to the patient directly as well”.—C5
	4. “…if the presentation is in keeping with the assay report, we would treat it otherwise, probably not really. We would rely on our usual cultures”.—C2
	5. “…if the clinical suspicion is higher for a pneumonia or something bacterial if the array doesn’t show any bacterial organisms, we would wait for the culture rather than just ignoring the result. You would still continue the antibiotic at that point”.—C2
	6. “I found it very useful in trying to identify any pathogens earlier than usual, which would then affect my decision as to whether to either have a narrow spectrum antibiotic, continue with broad-spectrum, or discontinue antibiotics altogether”. —C5
	7. “…if someone is on ceftriaxone or something like that, then you’ll change it to co-amoxiclav or something like that if there’s anaerobes in there”.—R2
	8. “…there were occasions when we had patients that maybe were intubated … [for]… something that wasn’t … lung pathology related (..) had a TAC that had some results of reasonable (.) kind of intermittent (intermediate) Ct values that weren’t particularly low that ended up on antibiotics for a longer period of time than perhaps they would have done previously. It’s difficult to say (.) a lot of patients on PICU end up on antibiotics for a longer period of time than they might do (on a ward) because they’re critically unwell, and I understand that. So (.) it’s difficult to say how much that information played into that, but that was something I noticed during the study”.—R1
	9. “I think more than anything; it helped us to stop antibiotics”.—R4
If clinicians still had a high degree of suspicion of secondary bacterial infection, they would continue antimicrobial therapy regardless of TAC results	10. “…if you’re quite concerned about secondary infection despite them having a viral infection, then I think you are going to say you might continue antibiotics. So yes, it guides, but you’re not removing your clinical hat”.—R2
A wait-and-see approach was used in patients with borderline cycle threshold results, with a plan for escalation of treatment if the patient deteriorated	11. “…when the Ct values are borderline, we might wait to depend on what the patient is doing – their clinical state, their inflammatory markers, as well as awaiting culture results. We might withhold escalating the treatment unless the patient is really sick, and then we would act on that”.—C3
Borderline TAC results were more likely to be used to direct treatment in severely unwell patients where other diagnostic tests had not identified a pathogen	12. “It is an adjunct to current practice rather than stand-alone evidence for infection. Depending on the clinical scenario. If someone is in sepsis or in shock, we would not ignore it”.—C2
TAC results were used for infection control decisions	13. “We’ve isolated children based on the TAC result”.—N6
	14. “We only have three cubicles, so I think it helped us make the decision whether to isolate them in a cubicle or not. Or whether we could leave them where they were. Either in a single bay or whether we could cohort them, I guess that would be the other thing. Which is very different from going to the ward because they isolate most viruses”.—N6
Nursing staff developed their clinical understanding of a patient’s physiology based on organisms found on TAC	15. “I think sometimes it’s just confirmation of why they are so unwell. I think that’s the thing. When you grow a specific virus, you often say it explains they do what they do. I think we are all very aware you can get a virus; viruses will cause certain children to behave in a particular way”.—N6
If unusual organisms were found on TAC, this led to immune screening in some patients	16. “I remember having one that had quite a few weird and wonderful sporadic ones, which, I think it led off to immune system testing because it wasn’t something you’d typically find from what I remember”. —N4
	17. “From what I remember, there were a couple that led to further testing of other things”.—N4

*C* Consultant (senior doctor), *N* Nurse, *PICU* Paediatric intensive care unit, *R* Registrar (senior doctor-in-training), *TAC* TaqMan array card

#### Staff recommendations for future research of diagnostic arrays

PICU staff reported that the format in which TAC results were reported was important in avoiding test misinterpretation (Table S11, Quote 1). Staff recommended a control group without respiratory infection that could identify background detection of bacteria on TAC in future studies to help determine the relevance of intermediate results (Table S11, Quote 2). TAC was used for both screening and diagnostic purposes—some staff felt it could be used on all ventilated PICU admissions whilst others felt there should be specific indications to request a TAC (Table S11, Quotes 3–9). Economic analysis was considered an important element of any future larger-scale implementation (Table S11, Quote 10).

## Discussion

This research highlights factors that influence prescribing behaviour of PICU prescribers and their perceptions following the implementation of a diagnostic array incorporating bacterial, fungal and viral pathogens for the first time. Most prescribers would request inflammatory markers in the setting of CAP and VAP, however only white cell count is included in the Centers for Disease Control and Prevention (CDC) paediatric pneumonia diagnostic criteria [7]. Full blood count and C-reactive protein (CRP) cannot differentiate between bacterial and viral infection in hospitalised children [28], so these investigations are potentially over-utilised for pneumonia diagnosis. Procalcitonin may have a role in ruling out bacterial co-infection [29]; however, it was a less frequently utilised by PICU prescribers in this survey.

Blood cultures were often requested, as recommended by the *British Society Guidelines* [30]; however, in practice, blood cultures are only positive in 1.1–1.5% of children hospitalised for pneumonia [31–33]. Unlike practice in adult intensive care [34], there was a preference towards sampling for culture using ETA over more invasive methods such as non-bronchoscopic bronchoalveolar lavage (mini-BAL) or formal bronchoscopy. Prescribers tended to rely on the clinical presentation and rated chest radiographs highly in making a diagnosis of VAP, as was the case in an antimicrobial decision-making study for adults with VAP [34, 35]. Procalcitonin was most frequently requested in the setting of treatment failure, which is supported by its ability to predict complicated pneumonia [36, 37].

The cessation of antimicrobial therapy was predominantly based on the patient’s clinical status rather than investigation results. This result may be due to a low perceived value of investigations to direct antimicrobial therapy after commencement or fear of missed diagnosis and the high morbidity and mortality of VAP [35]. Antimicrobial duration in critical care units is often determined based on the perception that the treatment will prevent adverse outcomes, perhaps due to the limitations of microbiology tests [14, 38]. In addition to objective indicators of the patient’s status, intuitive processes are described as a part of antimicrobial decision-making in PICU. This process may be caused by ‘gut feeling’, pattern recognition, or sometimes motivated by fear [38]. This is not something that was captured in the survey, but important to consider in antimicrobial prescribing behaviour. Prescribers felt that some features included in the CDC pneumonia criteria, including auscultation findings and new onset cough, were of limited relevance in their antimicrobial decision-making [7]. For pneumonia to be labelled bacterial in origin, according to these criteria, a bacterial organism must be isolated on microbiology culture, or a histopathological examination is required. This does not frequently occur in clinical practice [39, 40], hence the potential benefit of diagnostic arrays.

The range of reasons staff described using TAC, within the bounds of the protocol, was greater than anticipated. Diagnostic array studies to date have focussed on measures such as the test performance compared to routine investigations, impacts on antimicrobial prescriptions, length of stay and achievement of clinical cure [41]. Whilst these outcomes are important, they do not necessarily capture the nuance of how the tests may be used in clinical practice, both as a screening ‘rule out’ test and diagnostic ‘rule in’ test. This distinction reflects the approach taken by intensivists who make antimicrobial decisions based on a balance of risk. TAC may be a helpful addition to the ‘Sepsis 6’ investigations and interventions for children with suspected sepsis in the PICU [42]. In other situations, diagnostic tests are requested as a rule-in test, where there is a moderate to high pre-test probability, but morbidity and mortality outcomes may be moderate [43]. The use of TAC appeared to be greatest as a rule-out test so that children with viral LRTI, with possible bacterial co-infection, could have earlier antimicrobial therapy cessation. This approach has been previously highlighted as a benefit of diagnostic arrays, and clinicians have reported they would consider antimicrobial cessation based on negative results [14, 43]. If a narrower range of pathogens were incorporated on the array there would be less certainty in ruling out the potential of co-infection. As demonstrated by an adult intensive care study of custom TAC, it is critical that the targets captured by the molecular diagnostic are wide ranging and incorporate the most common pathogens for the disease of interest [44]. Without this range, clinicians may not have confidence that the test has excluded co-infection [44]. Previous investigations of a narrower range TAC in adult patients with suspected VAP identified that multiple episodes of potential antimicrobial rationalisation were not performed [44]. The authors highlighted ‘clinician education and buy-in’ as necessities for the effective implementation of diagnostic arrays [44], as identified in the present study.

This study had limitations—the survey had a lower response rate than was anticipated, which may have been due to a lack of awareness of the survey or survey fatigue. This was most notably in continental Europe, whilst the response rates in Australasia and the UK were higher. The survey was conducted during winter in the Northern Hemisphere, which may have influenced the number of patients admitted to PICUs with pneumonia and receiving antimicrobial therapy. Most respondents were from high-income countries; hence investigations and prescribing would have been weighted towards practice in this setting. It is important to note that although we obtained prescribers’ opinions on antimicrobial decision-making, this may not necessarily reflect actual clinical practice. The interviews were limited by being a single-centre and the non-randomised selection of participants. Participation was low in nursing staff compared to medical staff; however, this was due to rapid thematic saturation in this craft group.

## Conclusions

PICU prescribers rate diagnostic arrays highly when making antimicrobial prescribing decisions. These tests are considered to be of value for both screening and diagnostic purposes. Researchers should consider sensitivity, interpretation, and reporting of diagnostic arrays when designing a future dedicated paediatric randomised control trial of this technology.

## Supplementary Information

**Additional file 1: Fig. S1.** Location of survey participants. **Table S2.** Estimation of the rates of antimicrobial use and pneumonia in mechanically ventilated children. **Table S3.** Clinical features used by clinicians to make prescribing decisions for community acquired and ventilator associated pneumonia. **Fig. S2.** Rating of clinical factors used by clinicians making prescribing decisions (a) community-acquired and (b) ventilator-associated pneumonia. **Table S4.** Investigations used by clinicians to make prescribing decisions for community-acquired and ventilator-associated pneumonia. **Table S5.** Factors raising concern for clinicians that antimicrobial therapy is failing to treat respiratory infection. **Fig. S3.** Rating of the importance of clinical features of patients in the escalation of antimicrobial therapy. **Table S6.** Investigations requested by clinicians in the setting of failed treatment of respiratory infection in mechanically ventilated children. **Fig. S4.** Rating of the importance of investigation of patients in which antimicrobial therapy is failing to treat respiratory infection. **Table S7.** Factors considered by prescribers in the cessation of antimicrobial therapy for respiratory infection. **Fig. S5.** Rating of the importance of investigations for patients in the cessation of antimicrobial therapy. **Table S8.** Benefits and challenges of the integration of a custom TaqMan array card into clinical practice – Quotations supporting thematic analysis. **Table S9.** Purposes of the TaqMan array card – Quotations supporting thematic analysis. **Table S10.** Interpretation of TaqMan array card – Quotations supporting thematic analysis. **Table S11.** Future research recommendations – Supporting quotations of thematic analysis

**Additional file 2:** PRAMS survey.

**Additional file 3:** RASCALS interview guide.

**Additional file 4:** Participant consent form.

**Additional file 5:** Participant information sheet.

**Additional file 6:** Raw data.

## Data Availability

The interview transcripts and survey data are available at the Open Science Framework: A qualitative investigation of paediatric intensive care staff attitudes towards the diagnosis of lower respiratory tract infection in the molecular diagnostics era [Internet]. OSF; 2023. Available from: DOI 10.17605/OSF.IO/7STEB.

## Abbreviations

ANZICS-PSG: Australian and New Zealand Intensive Care Society Paediatric Study Group
CAP: Community-acquired pneumonia
CDC: Centers for Disease Control and Prevention
CHERRIES: Checklist for Reporting Results of Internet E-Surveys
COREQ: Consolidated criteria for reporting qualitative studies
CRP: C-reactive protein
Ct: Cycle threshold
ESPNIC: European Society of Paediatric and Neonatal Intensive Care
ETA: Endotracheal tube aspirate
LRTI: Lower respiratory tract infection
Mini-BAL: Non-bronchoscopic bronchoalveolar lavage
PCCS-SG: Paediatric Critical Care Society Study Group, UK
PICU: Paediatric intensive care unit
TAC: TaqMan array card
VAP: Ventilator-associated pneumonia

## References

[1] Jain S, Williams DJ, Arnold SR (2015). Community-acquired pneumonia requiring hospitalization among U.S. children. N Engl J Med.

[2] Global Burden of Disease Collaborators (2017). Global, regional, and national age-sex specific mortality for 264 causes of death, 1980–2016: a systematic analysis for the Global Burden of Disease Study 2016. Lancet.

[3] Clark J, White D, Daubney E (2021). Low diagnostic yield and time to diagnostic confirmation results in prolonged use of antimicrobials in critically ill children. Wellcome Open Res.

[4] Pandolfo AM, Horne R, Jani Y, et al (2021) Understanding decisions about antibiotic prescribing in ICU: an application of the Necessity Concerns Framework. BMJ Qual Saf. 10.1136/bmjqs-2020-012479 10.1136/bmjqs-2020-012479 PMC8899486 34099497

[5] Goodman D, Crocker ME, Pervaiz F (2019). Challenges in the diagnosis of paediatric pneumonia in intervention field trials: recommendations from a pneumonia field trial working group. Lancet Respir Med.

[6] Foglia E, Meier MD, Elward A (2007). Ventilator-associated pneumonia in neonatal and pediatric intensive care unit patients. Clin Microbiol Rev.

[7] National Health Safety Network (2023) Pneumonia (Ventilator-associated [VAP] and non-ventilator- associated Pneumonia [PNEU]) Event. Centers for Disease Control and Prevention. Atlanta. https://www.cdc.gov/nhsn/pdfs/pscmanual/6pscvapcurrent.pdf.

[8] da Silva PSL, de Aguiar VE, de Carvalho WB, Machado Fonseca MC (2014). Value of clinical pulmonary infection score in critically ill children as a surrogate for diagnosis of ventilator-associated pneumonia. J Crit Care.

[9] Hellyer TP, McAuley DF, Walsh TS (2020). Biomarker-guided antibiotic stewardship in suspected ventilator-associated pneumonia (VAPrapid2): a randomised controlled trial and process evaluation. Lancet Respir Med.

[10] Hellyer TP, McAuley DF, Walsh TS (2020). More research is required to understand factors influencing antibiotic prescribing in complex conditions like suspected ventilator-associated pneumonia. Ann Transl Med.

[11] Brookes-Howell L, Hood K, Cooper L (2012). Clinical influences on antibiotic prescribing decisions for lower respiratory tract infection: a nine country qualitative study of variation in care. BMJ Open.

[12] Kraus EM, Pelzl S, Szecsenyi J, Laux G (2017). Antibiotic prescribing for acute lower respiratory tract infections (LRTI) - guideline adherence in the German primary care setting: an analysis of routine data. PLoS ONE.

[13] Gotta V, Baumann P, Ritz N (2017). Drivers of antibiotic prescribing in children and adolescents with febrile lower respiratory tract infections. PLoS ONE.

[14] Pandolfo AM, Horne R, Jani Y (2022). Understanding decisions about antibiotic prescribing in ICU: an application of the Necessity Concerns Framework. BMJ Qual Saf.

[15] Pandolfo AM, Horne R, Jani Y (2021). Intensivists’ beliefs about rapid multiplex molecular diagnostic testing and its potential role in improving prescribing decisions and antimicrobial stewardship: a qualitative study. Antimicrob Resist Infect Control.

[16] Clark JA, Kean IRL, Curran MD (2021). Rapid Assay for Sick Children with Acute Lung infection Study (RASCALS): diagnostic cohort study protocol. BMJ Open.

[17] Clark JA, Conway Morris A, Curran MD (2023). The rapid detection of respiratory pathogens in critically ill children. Crit Care.

[18] Eysenbach G (2004). Improving the quality of web surveys: the checklist for reporting results of Internet E-Surveys (CHERRIES). J Med Internet Res.

[19] Joshi A, Kale S, Chandel S, Pal D (2015). Likert scale: explored and explained. Br J Appl Sci Technol.

[20] Harris PA, Taylor R, Thielke R (2009). Research electronic data capture (REDCap)—a metadata-driven methodology and workflow process for providing translational research informatics support. J Biomed Inform.

[21] R Studio Team (2022). RStudio: Integrated Development for R.

[22] Wickham H (2016). ggplot2: Elegant Graphics for Data Analysis.

[23] Tong A, Sainsbury P, Craig J (2007). Consolidated criteria for reporting qualitative research (COREQ): a 32-item checklist for interviews and focus groups. Int J Qual Health Care.

[24] DeJonckheere M, Vaughn LM (2019). Semistructured interviewing in primary care research: a balance of relationship and rigour. Fam Med Community Health.

[25] Sofaer S (1999). Qualitative methods: what are they and why use them. Health Serv Res.

[26] QRS International Pty Ltd (2019). NVivo.

[27] Braun V, Clarke V (2006). Using thematic analysis in psychology. Qual Res Psychol.

[28] Erixon ER, Cunningham KJ, Schlicher AN (2020). Use of procalcitonin for identification of cobacterial pneumonia in pediatric patients. J Pediatr Pharmacol Ther.

[29] Stockmann C, Ampofo K, Killpack J (2018). Procalcitonin accurately identifies hospitalized children with low risk of bacterial community-acquired pneumonia. J Pediatric Infect Dis Soc.

[30] Harris M, Clark J, Coote N (2011). British Thoracic Society guidelines for the management of community acquired pneumonia in children: update 2011. Thorax.

[31] Davis TR, Evans HR, Murtas J (2017). Utility of blood cultures in children admitted to hospital with community-acquired pneumonia. J Paediatr Child Health.

[32] Youssef AS, Fanous M, Siddiqui FJ (2020). Value of blood cultures in the management of children hospitalized with community-acquired pneumonia. Cureus.

[33] McCulloh RJ, Koster MP, Yin DE (2015). Evaluating the use of blood cultures in the management of children hospitalized for community-acquired pneumonia. PLoS One.

[34] Kenaa B, O’Hara NN, O’Hara LM (2022). Understanding healthcare provider preferences for ordering respiratory cultures to diagnose ventilator associated pneumonia: a discrete choice experiment. Antimicrob Steward Healthc Epidemiol.

[35] Kenaa B, O’Hara LM, Richert ME (2022). A qualitative assessment of the diagnosis and management of ventilator-associated pneumonia among critical care clinicians exploring opportunities for diagnostic stewardship. Infect Control Hosp Epidemiol.

[36] Nascimento-Carvalho CM, Cardoso M-RA, Barral A (2010). Procalcitonin is useful in identifying bacteraemia among children with pneumonia. Scand J Infect Dis.

[37] Ratageri VH, Panigatti P, Mukherjee A (2022). Role of procalcitonin in diagnosis of community acquired pneumonia in Children. BMC Pediatr.

[38] Fontela PS, Gaudreault J, Dagenais M (2022). Clinical reasoning behind antibiotic use in PICUs: a qualitative study*. Pediatr Crit Care Med.

[39] Versporten A, Bielicki J, Drapier N (2016). The Worldwide Antibiotic Resistance and Prescribing in European Children (ARPEC) point prevalence survey: developing hospital-quality indicators of antibiotic prescribing for children. J Antimicrob Chemother.

[40] Clark J, White D, Daubney E (2022). Low diagnostic yield and time to diagnostic confirmation results in prolonged use of antimicrobials in critically ill children. Wellcome Open Res.

[41] Brigadoi G, Gastaldi A, Moi M (2022). Point-of-care and rapid tests for the etiological diagnosis of respiratory tract infections in children: a systematic review and meta-analysis. Antibiotics.

[42] Daniels R, Nutbeam T, McNamara G, Galvin C (2011). The sepsis six and the severe sepsis resuscitation bundle: a prospective observational cohort study. Emerg Med J.

[43] Conway Morris A, Bos LDJ, Nseir S (2022). Molecular diagnostics in severe pneumonia: a new dawn or false promise?. Intensive Care Med.

[44] Jones NK, Conway Morris A, Curran MD (2020). Evaluating the use of a 22-pathogen TaqMan array card for rapid diagnosis of respiratory pathogens in intensive care. J Med Microbiol.

