# Supplementary material for: A qualitative investigation of paediatric intensive care staff attitudes towards the diagnosis of lower respiratory tract infection in the molecular diagnostics era
Source: Intensive Care Med Paediatr Neonatal. 2023 Jul 7;1(1):10. doi: 10.1007/s44253-023-00008-z (PMC10329081; doi:10.1007/s44253-023-00008-z)
Supplement: Supplementary file 1 — Additional file 1: Fig. S1. Location of survey participants. Table S2. Estimation of the rates of antimicrobial use and pneumonia in mechanically ventilated children. Table S3. Clinical features used by clinicians to make prescribing decisions for community acquired and ventilator associated pneumonia. Fig. S2. Rating of clinical factors used by clinicians making prescribing decisions (a) community-acquired and (b) ventilator-associated pneumonia. Table S4. Investigations used by clinicians to make prescribing decisions for community-acquired and ventilator-associated pneumonia. Table S5. Factors raising concern for clinicians that antimicrobial therapy is failing to treat respiratory infection. Fig. S3. Rating of the importance of clinical features of patients in the escalation of antimicrobial therapy. Table S6. Investigations requested by clinicians in the setting of failed treatment of respiratory infection in mechanically ventilated children. Fig. S4. Rating of the importance of investigation of patients in which antimicrobial therapy is failing to treat respiratory infection. Table S7. Factors considered by prescribers in the cessation of antimicrobial therapy for respiratory infection. Fig. S5. Rating of the importance of investigations for patients in the cessation of antimicrobial therapy. Table S8. Benefits and challenges of the integration of a custom TaqMan array card into clinical practice – Quotations supporting thematic analysis. Table S9. Purposes of the TaqMan array card – Quotations supporting thematic analysis. Table S10. Interpretation of TaqMan array card – Quotations supporting thematic analysis. Table S11. Future research recommendations – Supporting quotations of thematic analysis [file 44253_2023_8_MOESM1_ESM.pdf]

Supplementary information - A qualitative investigation of paediatric intensive care staff attitudes towards the diagnosis of lower respiratory tract infection in the molecular diagnostics era.

John A Clark [1, 2*], Andrew Conway Morris [2, 3, 4], Constantinos Kanaris [2, 5], David Inwald [2], Warwick Butt [6, 7], Joshua Osowicki [8], Luregn Schlapbach [9], Martin D Curran [10], Deborah White [2], Esther Daubney [2], Shruti Agrawal [1, 2], Vilas Navapurkar [2], M. Estée Török [2, 11], Stephen Baker [12], and Nazima Pathan [1, 2]

1. Department of Paediatrics, University of Cambridge, UK
2. Cambridge University Hospitals NHS Foundation Trust, UK
3. Division of Anaesthesia, Department of Medicine, University of Cambridge, UK
4. Division of Immunology, Department of Pathology, University of Cambridge, UK
5. Blizard Institute, Queen Mary University of London, UK
6. Paediatric Intensive Care Unit, Royal Children's Hospital Melbourne
7. Department of Critical Care, University of Melbourne
8. Infectious Diseases Unit, Department of General Medicine, Royal Children's Hospital Melbourne
9. Department of Intensive Care and Neonatology, University Children's Hospital Zurich
10. United Kingdom Health Security Agency, Clinical Microbiology and Public Health Laboratory, Cambridge, UK
11. Division of Infectious Diseases, Department of Medicine, University of Cambridge, UK
12. Cambridge Institute of Therapeutic Immunology and Infectious Disease, University of Cambridge, UK

*Corresponding author: Dr John Clark, Department of Paediatrics, Level 8, Addenbrooke's Hospital, Cambridge Biomedical Campus, Cambridge CB2 0QQ, UK. Email: jac302@cam.ac.uk

<u>Supplemental methods</u>

Survey study design

Data collection methods

The questionnaire was tested online by twenty-two prescribers through the lead author's network. Minor modifications were made to make the questions clearer in description and to allow respondents to provide additional responses to questions. The study population was PICU antimicrobial prescribers. Although the survey targeted individuals working in centres within the UK, continental Europe and Australasia, additional responses from other locations were included. Question order was not randomised. Participants could return to review responses prior to submission of the survey. No incentives were offered for survey participation. Given the data capture instrument provided a unique identifier to each survey participant multiple responses were considered unlikely, however this was not confirmed with participant registration, IP checks or log file analysis. The instrument integrates completeness checks.

Sample characteristics

The total potential participants were 339 members of the European Society of Paediatric and Neonatal Intensive Care (ESPNIC), 296 members of the Australian and New Zealand Intensive Care Society Paediatric Study Group (ANZICS-PSG) and 120 members of the Paediatric Critical Care Society Study Group, UK (PCCS-SG). From the total cohort of 755, the sample size was 72 (10%). This was lower than the target of a 20% response rate. The total view rate of the survey is unknown. The survey was fully completed by 59/72 (82%) of participants. Incomplete surveys were included where a full response was given to at least the first question.

Interview study design

Interviews were conducted by JC, a senior, male, paediatric registrar and PhD candidate. The interviewer received guidance from the Winton Centre for Risk and Evidence Communication and academic staff of the University of Cambridge School of Clinical Medicine. The interviewer had previous interaction with participants as a doctor working for the PICU and retrieval service. Participants were aware that in the context described herein, the interviewer was a research investigator.

Participants were advised of the study via staff email and approached by the interviewer face-to-face. A convenience sampling method was used, to ensure that interviewing would not disturb clinical work. An approach to participate only occurred during day shifts where there were sufficient levels of staffing to ensure that patients were not negatively impacted by the research. Interviews were undertaken in clinical offices. Feedback was not obtained from participants relating to the findings. It was not possible to record field notes. Interview transcripts were not provided to participants for comment. Interviews were not repeated.

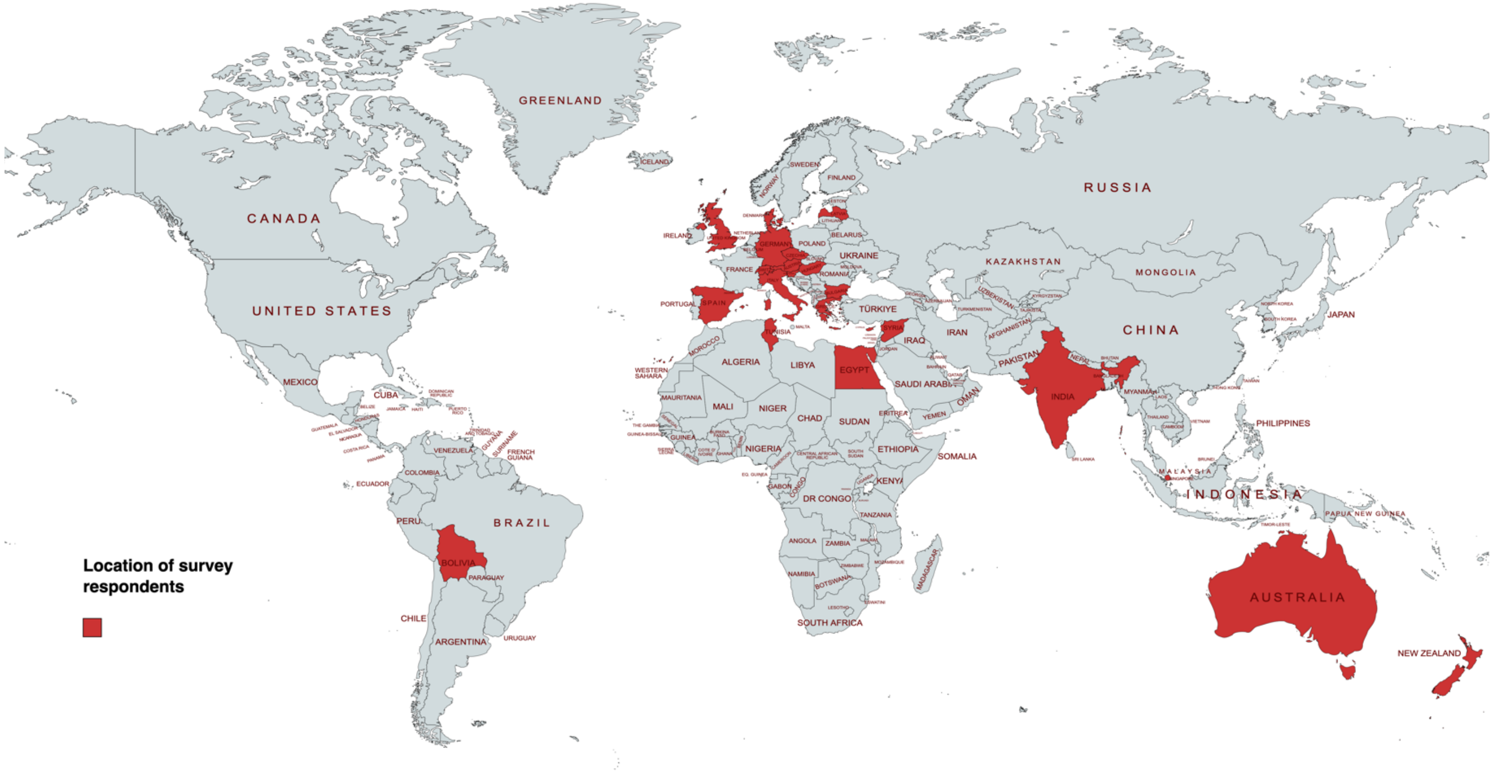

Continent	*N* (%)	Country	*N* (%)
Africa	3 (4)	Egypt	1 (1)
		Syria	1 (1)
		Tunisia	1 (1)
Asia	6 (8)	India	4 (6)
		Singapore	2 (3)
Australasia	11 (15)	Australia	10 (14)
		New Zealand	1 (1)
Europe	51 (71)	Austria	1 (1)
		Bulgaria	1 (1)
		Cyprus	1 (1)
		Czech Republic	1 (1)
		Denmark	1 (1)
		Germany	1 (1)
		Greece	4 (6)
		Hungary	2 (3)
		Italy	4 (6)
		Latvia	1 (1)
		Slovenia	1 (1)
		Spain	2 (3)
		Switzerland	1 (1)
		United Kingdom	30 (42)
South America	1 (1)	Bolivia	1 (1)
**Total**	**72 (100)**		**72 (100)**

Fig. S1 Location of survey participants

This figure was created with mapchart.net

Table S2 Estimation of the rates of antimicrobial use and pneumonia in mechanically ventilated children.

PICU	Region	Ventilated admissions	Patients on systemic antibiotics		Patients with CAP		Patients with VAP	
		N	*N*	(%)	*N*	(%)	*N*	(%)
A*	Australasia	11.3	6.3	(56)	1.3	(12)	1	(9)
B	Australasia	8	6	(75)	0	(0)	2	(25)
C	Continental Europe	1	1	(100)	0	(100)	?	?
D	Continental Europe	5	5	(100)	0	(0)	2	(40)
E	Continental Europe	2	0	(0)	0	(0)	0	(0)
F	Continental Europe	3	3	(100)	2	(67)	1	(33)
G	Continental Europe	8	3	(37.5)	0	(0)	0	(0)
H*	Continental Europe	3	3	(100)	0.3	(11)	0	(0)
I*	Continental Europe	9	8	(88.9)	2	(22)	1	(11)
J	Continental Europe	3	2	(66.7)	0	(0)	1	(33)
K	Continental Europe	4	2	(50)	1	(25)	0	(0)
L	UK	3	1.5	(50)	0	(0)	0	(0)
N*	UK	3.25	3.2	(100)	0.5	(15)	0	(0)
O	UK	3	2	(66.6)	0	(0)	0	(0)
P	UK	9	6	(66.7)	3	(33)	1	(11)
Q*	UK	8	4.3	(54.2)	0.5	(6)	1.5	(19)
R	UK	4	3	(75)	2	(50)	0	(0)
**TOTAL**		**87.5**	**59.4**	**(67.9)**	**13.7**	**(16)**	**10.5**	**(12)**

* Mean number used to calculate number of admissions in these centres due to responses from multiple clinicians; ?: no response to this question; CAP: community acquired pneumonia; PICU: paediatric intensive care unit; VAP: ventilator associated pneumonia

Table S3 Clinical features used by clinicians to make prescribing decisions for community acquired and ventilator associated pneumonia.

**Community acquired pneumonia** Responses = 72				**Ventilator associated pneumonia** Responses = 63			
**Clinical factor**	**Rank**	***N* (%)**	**Relevance – median % (IQR)**	**Clinical factor**	**Rank**	***N* (%)**	**Relevance – median % (IQR)**
Immunosuppression of the patient	1	65 (90)	88.0 (74.3 – 98.0)	Increased ventilatory requirements	1	54 (86)	79.0 (71.0 – 88.0)
History of chronic respiratory disease	2	61 (85)	65.0 (38.8 – 88.0)	Fever	2	52 (83)	75.0 (50.0 – 87.0)
Known colonisation of the respiratory tract	3	56 (78)	72.0 (39.3 – 83.8)	Increased oxygen requirement	3	51 (81)	77.0 (68.0 – 88.0)
Known/previous antimicrobial resistance	4	52 (72)	66.0 (0 – 84.5)	Immunosuppression of the patient	4	49 (78)	82.0 (56.0 – 96.0)
Fever	5	50 (69)	61.0 (0 – 75.8)	Quality or quantity of secretions	= 5	47 (75)	74.0 (0 – 84.0)
Age of the patient	6	45 (63)	44.0 (0 – 76.0)	Generalised clinical deterioration	= 5	47 (75)	81.0 (0 – 97.0)
Tracheostomy/home ventilation	= 7	44 (61)	59.0 (0 – 76.0)	History of chronic respiratory disease	6	34 (54)	50.0 (0 – 76.0)
New/increased production of respiratory secretions	= 7	44 (61)	61.5 (0 – 78.8)	Known colonisation of the respiratory tract	7	37 (59)	58.0 (0 – 76.0)
Oxygen requirements	= 9	42 (58)	62.0 (0 – 81.2)	Duration of mechanical ventilation	8	33 (52)	56.0 (0 – 77.0)
Chronic underlying medical condition	= 9	42 (58)	42.0 (0 – 64.0)	Known/previous antimicrobial resistance	9	28 (44)	0 (0 – 75.0)
Findings on auscultation	11	37 (51)	22.5 (0 – 67.0)	Chronic underlying medical condition	10	26 (41)	0 (0 – 63.0)
Escalation of respiratory support	12	30 (41)	0 (0 – 75.6)	Findings on auscultation	11	22 (35)	0 (0 – 63.0)
Ventilator pressure requirements	13	29 (40)	0 (0 – 68.8)	None of the above	N/A	0 (0)	
New cough	14	21 (29)	0 (0 – 55.3)				
Respiratory rate (prior to intubation)	15	18 (25)	0 (0 – 30.0)				
None of the above	16	1 (1)					

IQR: interquartile range

a.

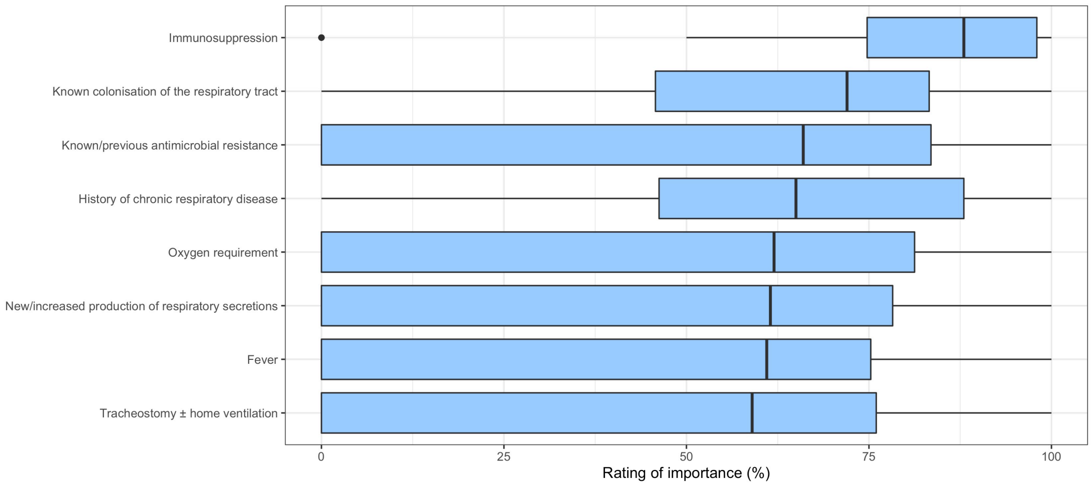

b.

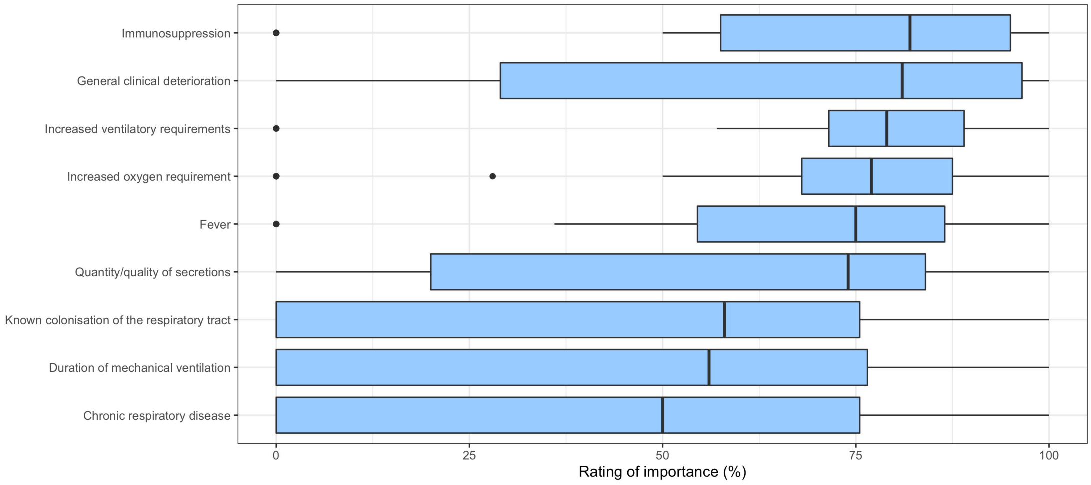

Fig. S2 Rating of clinical factors used by clinicians making prescribing decisions (a) community-acquired and (b) ventilator-associated pneumonia.

This box and whisker plot represents the perceived relevance, according to prescribers, of clinical features contributing to the decision to commence antimicrobial therapy in ventilated children with suspected respiratory infection. Only the highest-ranking clinical features, which had a median ≥ 50%, are represented.

Table S4 Investigations used by clinicians to make prescribing decisions for community-acquired and ventilator-associated pneumonia

**Investigation**	**Community acquired pneumonia** Responses = 72			**Ventilator associated pneumonia** Responses = 63			***p*-value***
	**Rank**	***N* (%)**	**Relevance – median % (IQR)**	**Rank**	***N* (%)**	**Relevance – median % (IQR)**	
Inflammatory markers (any)	1	71 (99)		2	59 (94)		0.336
C-reactive protein		60 (83)	72.0 (64.0 – 87.0)		52 (83)	76.5 (61.8 – 87.8)	1.000
Full blood count		63 (88)	68.0 (61.0 – 82.0)		53 (84)	73.0 (61.0 – 85.5)	0.626
Procalcitonin		33 (46)	80.0 (71.5 – 99.5)		33 (52)	82.0 (72.5 – 97.0)	0.493
Chest radiograph	2	70 (97)	80.0 (65.5 – 90.5)	1	63 (100)	82.0 (72.0 – 94.0)	0.499
Viral PCR panel (any)	3	64 (89)		4	47 (75)		1.000
Viral PCR panel – BAL		3 (4)	97.0 (97.0 – 98.5)		3 (5)	77.0 (76.5 – 86.5)	1.000
Viral PCR panel – ETT aspirate		25 (35)	75.0 (64.0 – 91.0)		27 (43)	76.0 (66.0 – 86.0)	0.378
Viral PCR panel – mini-BAL		13 (18)	74.0 (68.0 – 92.0)		10 (16)	82.5 (77.5 – 84.5)	0.821
Viral PCR panel - NPA		36 (50)	83.0 (72.0 – 95.5)		20 (32)	76.0 (62.8 – 83.0)	0.037
Viral PCR panel – NP swab		25 (35)	81.5 (66.0 – 97.8)		10 (16)	83.0 (77.5 – 93.0)	0.018
Culture – respiratory (any)	4	60 (83)		3	58 (92)		0.193
Culture – BAL		6 (8)	95.5 (83.8 – 98.5)		8 (13)	97.5 (84.3 – 99.5)	0.573
Culture – ETT aspirate		53 (74)	82.0 (70.0 – 95.5)		46 (73)	81.0 (73.5 – 98.0)	1.000
Culture - mini-BAL		16 (22)	85.6 (71.0 – 96.8)		8 (13)	85.0 (74.5 – 93.8)	0.179
Culture - blood	5	57 (79)	74.0 (53.0 – 89.5)	6	37 (59)	74.0 (50.0 – 87.5)	0.014
Blood gas	6	55 (76)	59.5 (45.8 – 82.3)	5	41 (65)	67.0 (50.0 – 82.0)	0.184
Multi-pathogen array (any)	7	41 (57)		7	28 (44)		0.170
Multi-pathogen array – BAL		6 (8)	88.0 (75.8 – 96.5)		5 (8)	97.0 (77.5 – 99.0)	1.000
Multi-pathogen array – ETT aspirate		31 (43)	83.0 (74.0 – 95.5)		19 (30)	81.0 (69.0 – 96.0)	0.153
Multi-pathogen array – mini-BAL		15 (21)	79.0 (50.0 – 85.0)		13 (21)	80.0 (75.0 – 91.0)	1.000
Chest ultrasound	8	24 (33)	76.0 (64.0 – 91.0)	8	21 (33)	83.0 (73.0 – 89.0)	1.000
Urinary pneumonia antigens	9	14 (19)	61.0 (50.0 – 84.3)	9	3 (5)	84.0 (84.0 – 88.5)	0.017
16S/18S rRNA gene sequencing - blood	= 10	1 (1)	93	10	1 (2)	83	1.000
16S/18S rRNA gene sequencing - respiratory	= 10	1 (1)	66		0 (0)		1.000
None of the above		0 (0)			0 (0)		1.000

* Fishers exact test comparing frequency investigation is requested for community acquired pneumonia and ventilator associated pneumonia; BAL: bronchoalveolar lavage; ETT: endotracheal tube; mini-BAL: non-bronchoscopic bronchoalveolar lavage; IQR: interquartile range; NP: nasopharyngeal; NPA: nasopharyngeal aspirate

Table S5 Factors raising concern for clinicians that antimicrobial therapy is failing to treat respiratory infection

**Clinical factor**	***N* (%)**	**Rank**	**Relevance - median %, (IQR)**
Ventilator requirements increased	52 (88)	1	79.0 (66.0 – 90.0)
Oxygen requirements increased	45 (76)	2	75.0 (55.0 – 94.0)
Fever	41 (69)	3	73.0 (0 – 84.0)
Quality/quantity of secretions	29 (49)	= 4	0 (0 – 81.0)
Haemodynamic instability	29 (49)	= 4	0 (0 – 87.0)
Respiratory rate	10 (17)	= 6	0 (0 – 0)
Findings on auscultation	10 (17)	= 6	0 (0 – 0)

NB: There were 59 responses to this question; IQR: inter-quartile range

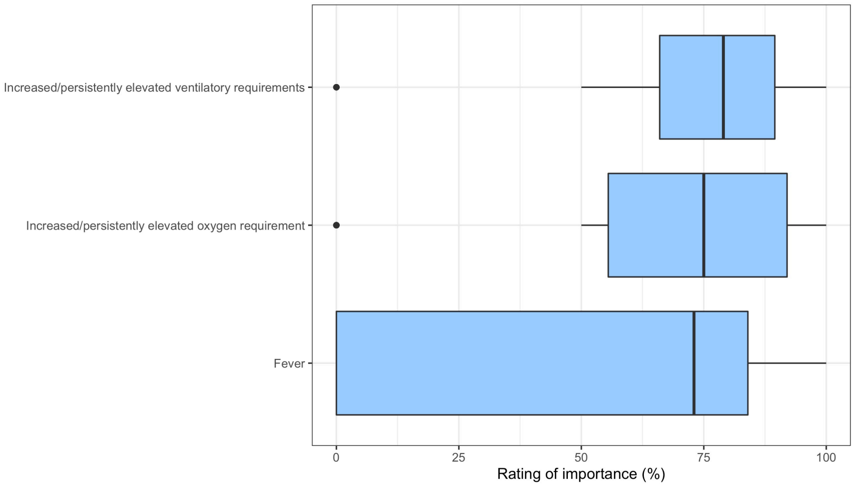

Fig. S3 Rating of the importance of clinical features of patients in the escalation of antimicrobial therapy
This box and whisker plot represents the perceived relevance, according to prescribers, of clinical features contributing to the decision to escalate antimicrobial therapy in ventilated children with suspected respiratory infection. Only the highest-ranking clinical features, which had a median ≥ 50%, are represented.

Table S6 Investigations requested by clinicians in the setting of failed treatment of respiratory infection in mechanically ventilated children

Investigation	Rank	*N* (%)
Inflammatory markers (any)	1	55 (93)
C-reactive protein		44 (75)
Full blood count		39 (66)
Procalcitonin		36 (61)
Chest radiograph	2	47 (80)
Cultures – respiratory (any)	3	40 (68)
Culture – ETT aspirate		26 (44)
Culture - mini-BAL		21 (36)
Culture – BAL		4 (7)
Blood gas	4	26 (44)
Multi-pathogen array (any)	5	22 (37)
Multi-pathogen array – mini-BAL		14 (24)
Multi-pathogen array – ETT aspirate		11 (19)
Multi-pathogen array – BAL		3 (5)
Culture - blood	6	21 (36)
Viral PCR panel (any)	7	20 (34)
Viral PCR panel – ETT aspirate		11 (19)
Viral PCR panel - NPA		8 (14)
Viral PCR panel – mini-BAL		8 (14)
Viral PCR panel – NP swab		3 (5)
Viral PCR panel – BAL		1 (2)
Chest ultrasound	8	16 (27)
Urinary pneumonia antigens	9	4 (7)
Other investigation	=10	1 (2)
16S/18S rRNA gene sequencing - blood	=10	1 (2)
16S/18S rRNA gene sequencing - respiratory	N/A	0 (0)
None of the above	N/A	0 (0)

NB: There were 59 responses to this question; BAL: bronchoalveolar lavage; ETT: endotracheal tube; mini-BAL: non-bronchoscopic bronchoalveolar lavage; IQR: interquartile range; NP: nasopharyngeal; NPA: nasopharyngeal aspirate

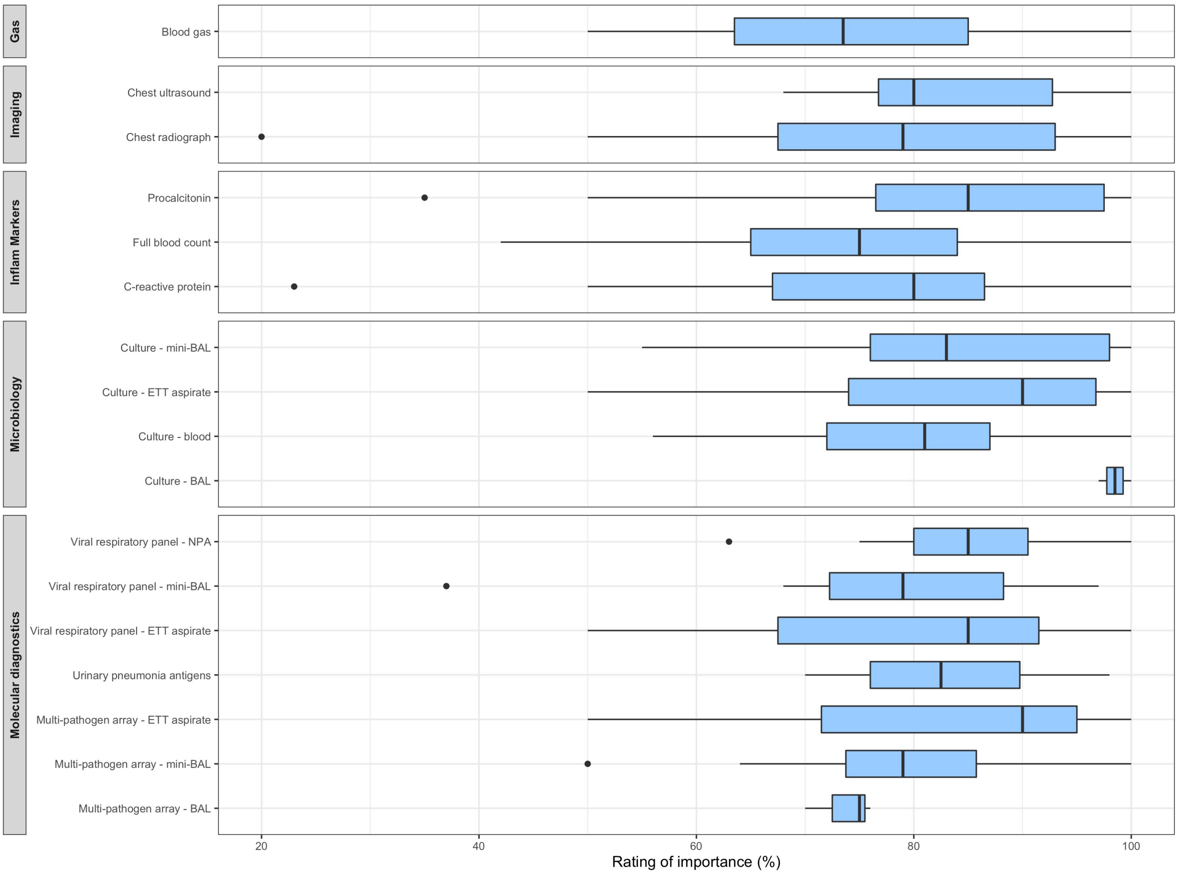

Fig. S4 Rating of the importance of investigation of patients in which antimicrobial therapy is failing to treat respiratory infection.

This box and whisker plot represents the perceived relevance, according to prescribers, of clinical features contributing to the decision to escalate antimicrobial therapy in ventilated children with suspected respiratory infection. BAL: bronchoalveolar lavage; ETT: endotracheal tube; mini-BAL: non-bronchoscopic bronchoalveolar lavage; NPA: nasopharyngeal aspirate

Table S7 Factors considered by prescribers in the cessation of antimicrobial therapy for respiratory infection

**Clinical factor**	***N* (%)**	**Rank**	**Relevance - median %, (IQR)**
Clinical/physiological parameters	54 (92)	1	85.0 (66.0 – 97.0)
Biochemical investigations	38 (64)	2	63.0 (0 – 80.0)
Microbiological investigations	35 (59)	3	63.0 (0 – 86.0)
Virological investigations	22 (37)	4	0 (0 – 81.0)
Imaging investigations	19 (32)	5	0 (0 – 61.0)
Pre-determined protocol regarding antimicrobial duration	18 (31)	6	0 (0 – 67.0)

NB: There were 59 responses to this question; IQR: interquartile range

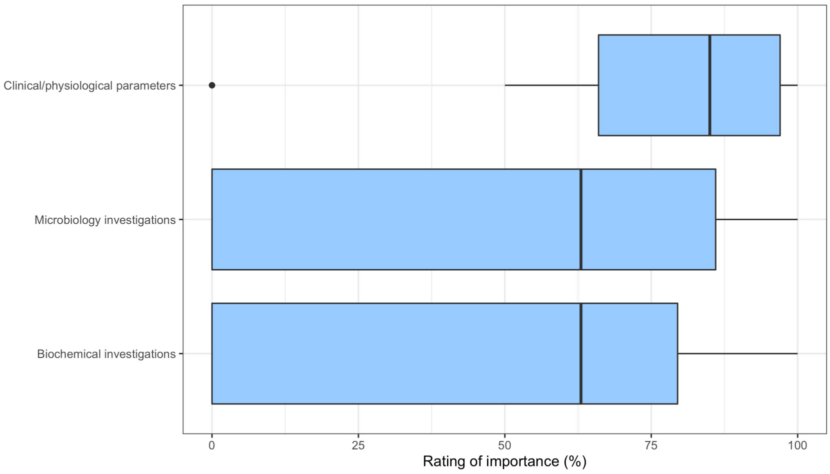

Fig. S5 Rating of the importance of investigations for patients in the cessation of antimicrobial therapy
This box and whisker plot represents the perceived relevance, according to prescribers, of investigation results contributing to the decision to cease antimicrobial therapy in ventilated children with suspected respiratory infection. Only the highest-ranking clinical features, which had a median ≥ 50%, are represented.

Table S8 Benefits and challenges of the integration of a custom TaqMan array card into clinical practice – Quotations supporting thematic analysis

Theme		Supporting quotations
The implementation of TAC was received positively by staff.	1.	"It was very good; I mean I think it was a very positive thing. It seemed to be useful in decision making…"- C1
	2.	"There was certainly quite a lot of excitement around it and to suddenly have something which would give us fairly quick and definitive results". – C4
TAC was considered a reliable investigation.	3.	"…there weren't cases where we then ended up having to backtrack and restart antibiotics … because something had been missed". – R4
TAC became integrated into routine practice on the PICU.	4.	"…someone would come in and say 'have we done the TAC'? and be proactively asking for it." – R4
	5.	"…the microbiology staff seem very accepting of it". - R3
	6.	"TAC has become a routine part of PICU life and a routine part of PICU testing. Just because of the ease of doing it, the speed at which it comes back, in its value in tailoring antimicrobial care". - C4
	7.	"There's probably not a ward round that goes by now without mentioning TAC. Which I guess is a sign of success." – C4
Whilst TAC was used as an adjunct to microbiology culture it sometimes replaced viral respiratory investigations. This was in part due to laboratory pressures during the COVID-19 pandemic.	8.	"…I don't think we stopped sending any of the other routine cultures [and] I wonder if we did a few less NPAs than we might have beforehand" – C1
	9.	"They weren't at one point even running [tests] for your basic rhinoviruses, the normal winter bugs you would get so actually the TAC gave us answers that weren't available to us at that point." – N3
Given the sensitivity of TAC, sometimes pathobionts were detected alongside clinically relevant pathogens. This caused some difficulties with test interpretation.	10.	"…when you have a very sensitive investigation relying on PCR sometimes things flag up as positive when perhaps they're not, they're just bystanders…". – C1
	11.	"We would get normal commensals as well". – C2
	12.	"Clinically we know this is not possible to have *stenotrophomonas* or something like that but it would still come up on the RASCAL study. That would sometimes confuse the clinical picture, I mean confuse the treating team. But at that point we would actually check with a microbiologist". – C2
	13.	"You'd sometimes get an almost zoo of different pathogens coming through, and it becomes very difficult to interpret that". – C3
	14.	"I think the biggest challenge we've had is dealing with the sensitivity of the test. Because we are getting a lot more positive results than we did on culture". – C4
	15.	"…the doctors would say, that it was throwing up red herrings and we're not going to treat it". – N1
	16.	"…there was too much in there and stuff that looked like it was potential contamination, so it wasn't really beneficial from that point of view because you didn't know if it's a true result or not" – N3
	17.	"…towards the end…people took less notice of it because they were a bit like 'oh yeah it shows up stuff but the kids aren't actually symptomatic with those it doesn't matter', so I think sometimes when there was something that was relevant it got missed because people had become so blasé about the fact that there were so many positives being picked up on it". – N2
	18.	"The issue we did raise…is when that's not in context which is either an organism you're not expecting, or it's detected at a relatively low level, so you think is that colonisation? Is it really a thing? But I think that's not a new decision, it's not a new process. I think almost every diagnostic test we have; we have to make that decision". – C4
Borderline cycle threshold values could be challenging for prescribers when making antimicrobial decisions.	19.	"But yeah, if you get a borderline Ct value, you're like, is this significant, is it not? Should we be treating it or should we not? I think that can be tricky, especially when you have potentially different clinicians who have a different view on that". – R4
Given additional pathogens were detected on TAC, this sometimes resulted in unanticipated infection control issues.	20.	"The sample might come up positive for something we weren't expecting and all of a sudden, we're like 'oh they're in the middle of the bay, we might need to put them in a cubicle', or 'they're okay to be here while they're in closed circuit ventilation but once extubated we might need to isolate' or vice versa". – N3

C: Consultant (senior doctor); N: Nurse; PICU: Paediatric intensive care unit; RASCAL: Rapid Assay for Sick Children with Acute Lung infection; R: Registrar (senior doctor-in-training); TAC: TaqMan array card

Table S9 Purposes of the TaqMan array card – Quotations supporting thematic analysis

Theme	Supporting quotations
Identification of a viral pathogen on TAC reassured clinicians that they could cease antimicrobial therapy.	1. "…those with a viral illness where we could stop antibiotics early, were the more frequent beneficiaries of the TAC". – C5
	2. "If there was [an] RSV positive [TAC] on such patients we would be more confident in stopping antibiotics, not waiting until the cultures are back. If RSV is positive we could stop acyclovir and ceftriaxone". – C2
	3. "It's … particularly useful in children like the RSV positive bronch's, because actually once you've got a clinical history of bronch' and then you've got a positive RSV often these children are on ceftriaxone and aciclovir and you can stop that because you know what's causing them to be sick".- N2
TAC was used to help identify microorganisms in complex patients known to have bacterial colonisation and recurrent hospital admissions.	4. "We're getting patients with more complicated histories, certainly more patients who have multiple hospital stays, multiple other problems and you're looking for organisms outside *Rhinovirus*, RSV, and all of that sort of thing. So certainly, it's making a difference for those sorts of patients". – C4
Clinicians used TAC to identify atypical pathogens in children that were immunosuppressed.	5. "…we're dealing with children who are on immunomodulation, for example, certainly looking for bacterial foci, looking for atypical foci, has become definitely a bigger part of our work". – C4
TAC was used to help in situations of diagnostic uncertainty.	6. "We had a child who came in who was a teenager who was ventilated, who had radiological features of ARDS with four quadrant airway shadowing, and we didn't have a diagnosis and the TAC came up as *Chlamydia psittaci*. So, we were able to make a diagnosis of psittacosis. Which is something I've never seen before in a child. And the investigation prompted us to go back and as the family if there was a parrot at home, and there was". – C1
	7. "…the most helpful thing I think is in a patient you had no other focus or no other bug for, and something flagged up positive all of a sudden, your mindset and your management changes as you're less worried that you're missing some horrendous abdominal sepsis and in fact you've got a guilty organism. So that was really helpful". – R5
	8. "I would use it perhaps if it were one of those patients that didn't come back on the routine, oh it's not COVID, it's not RSV (..) I might use it then... I think I would do other tests first". – N1
	9. "…if they've come in with any sort of respiratory symptoms or unknown (..) sepsis, temperature of unknown origin, it would probably be what we would think about from a nursing point of view". – N4
TAC was used to screen for suspected ventilator associated pneumonia.	10. "[We would use the test to consider]…whether or not the patient is developing VAP". – C3
	11. "…in cases of a more chronic child, someone being on the unit for longer and developing an early suspected VAP, TAC would allow us to detect that earlier. So that [was a] more direct and immediate gain to that population…"- C5
	12. "…the intubated patients who are quite unwell and you're suspecting VAP that [TAC] would certainly guide your treatment". – R3
	13. "…if you're worried about a VAP, you can use it couldn't you"? – R2
In situations where all existing diagnostic tests had been used, TAC sometimes provided a solution to diagnostic dilemmas.	14. "There was a diagnostic dilemma with this child that had nutritional deficiency (..) came in with unexplained fever and multisystem problems post-COVID infection and it was not certain whether this patient had PIMS-TS pathophysiology or not. And, despite having some early improvement, this child was then deteriorating again and it was unclear as to what the main driver of that deterioration was. He also had significant skin involvement so there was a question mark around translocation and development of secondary sepsis. TAC was extremely helpful as it gave us [the result of] *Aspergillus* which is what the patient had and then we could direct antifungal treatment specifically towards that. And this child had, had, prior to that (..) had had quite a lot of steroids as well as immune suppression because of the multisystem involvement and possibly that's what drove the *Aspergillus*. So, we would not have picked it up otherwise and it gave us an early result and we could titrate the treatment towards this specific pathogen". – C3
	15. "…having extra information particularly when you do have diagnostic dilemmas and diagnostic uncertainty then it's certainly going to be really useful".- C4

Table S9 cont.

TAC was also requested in children that were not responding to antimicrobial therapy.	16. "[An indication for TAC was when] they're not responding to treatment" – R2.
TAC provided an additional safety net, sometimes detecting unexpected pathogens.	17. "It occasionally picked up some rare things". – N1
	18. "…in conjunction with any other … studies or inflammatory markers or persistence of fever would be an extra sort of safeguard that we are making the right clinical choice" – C5
	19. "We're often looking for *Rhinovirus*, RSV, you know any of that so, it's confirmed our diagnosis you might say and sometimes it might throw up something useful you need to treat". – N3
TAC was considered by some staff as a component of routine screening for patients admitted to the PICU.	20. "In terms of the intensive care planning, it's useful if we have an early viral result to try to define whether the patient needs to be isolated and that has huge implications for the staffing and whether we can admit or not". – C3
	21. "It gets a good admission screen, as I think that's helpful". – R5
	22. "It's no different to when we do respiratory PCRs on admission anyway for those sorts of patients so […], it's not causing more work, we already have to do aspirates anyway, so it makes sense". – N4
Some parents were reassured to receive an early diagnosis through TAC.	23. "…it's confirmation for the parents. From a psychological point of view I think it helps when families are able to identify what has actually cause the problem for their children". – N6

ARDS: Acute respiratory distress syndrome; C: Consultant (senior doctor); N: Nurse; PICU: Paediatric intensive care unit; PIMS-TS: Paediatric multi-system inflammatory disorder temporally associated with SARS-CoV-2 infection; R: Registrar (senior doctor-in-training); RSV: *Respiratory syncytial virus*; TAC: TaqMan array card; VAP: ventilator-associated pneumonia

Table S10 Interpretation of TaqMan array card – Quotations supporting thematic analysis

Theme	Supporting quotations
TAC was an adjunct to respiratory cultures.	1. "It's quite unusual to get a positive bacteriological diagnosis so being able to have the information about bacteriology despite perhaps a negative culture was incredibly helpful…". – C1
	2. "I don't think it replaces a respiratory culture, so we continued to do those". – R4
	3. "…it's an adjunct rather than a replacement, but I suspect that comes down to lots of doctors wanting more data points. So, if you've got lots more data. You can add data points to your CRP, your cultures, your PCR then you feel like you have a fuller picture. If people said let's stop doing cultures and CRPs and lets just do a TAC people would push back". – R5
	4. "Over time it has become a really important part of the diagnostic process as much as a culture I would say. It's at that level". – C4
Staff consistently reported that they would consider the clinical history and patient status when interpreting the TAC results.	5. "First of all I would see if this fits into the clinical scenario. …Clinically if it's fitting, I would take it as this even though Ct values are on the higher side, not necessarily the cut-off of thirty. If not…we would go clinically…we wouldn't treat it unnecessarily". - C2
	6. "We have to interpret the results in context with the patient we're looking after. Part of that is obviously taking the clinical information and adding the diagnostic test to either confirm or deny what we've found. Rather than looking for something new that's suddenly going to change our management". – C4
	7. "there's probably an additional element of screening that you need to do of a positive result to say it's positive, sure, but is it actually positive in the context of the patient"? – C4
	8. "I guess we've got to choose wisely because it's annoying getting a result that you have to deal with when actually it's a bit of a distraction from the clinical picture".– R4
	9. "…it's important to know the patient's history…how they're ventilating, their x-rays, their inflammatory screen … it will be just one other tool, used in the context of all the other tools we had. With the old analogy of the jigsaw it will be once more piece to add to it. Is it a very useful piece? Yeah, and it's quite a big piece as well." – C4
Some staff had misconceptions relating to the scope of TAC and existing diagnostic tests. There was a tendency to overestimate the range of pathogens analysed on existing diagnostic tests, and underestimate the range of bacterial and fungal pathogens on the TAC.	10. "So *Strep*', *Pseudomonas*, I can't think of what else is on there now". – N2
	11. "…sometimes I didn't appreciate the extent of the number of things that you get back on TAC. For example, I think I remember there was one apnoeic young child where we said 'oh could this be *Pertussis*'? We ended up doing an upper nasal or NPA swab for *Pertussis* purely because I didn't appreciate, that it came back on the TAC". – R4
	12. "…we will often do the Biofire now, I think it has 30-40 viruses on it, quite a lot of viruses on it…" – N2
	13. "We use it in place of a standard respiratory viral PCR. That was my understanding". – R3
There was some difficulty for staff in recalling the direction of positivity on TAC based on cycle threshold.	14. "If the Ct score, is it the higher the number, the more likely there is to be a viral load? Or the lower number? I can't remember which way around it is now". - N2
	15. "…if there was a low Ct value then we would not be as excited as if it was super high. Have I got that the right way around? Other way around". – R5
Staff found the reporting of cycle threshold values helpful in placing a weighting on their confidence in the TAC results reported.	16. "The Ct values are useful". – R4
	17. "Having the Ct values gives you a higher degree of confidence rather than a positive/negative, where you think positive for *Rhinovirus* and *Enterovirus*, great, what are you going to do"? – R3
	18. "I think it probably gives you a better range of perhaps the load of the virus, and perhaps getting the actual viruses as opposed to maybe not getting anything". – N6
	19. "…if the Ct value is over 30, I tend to discount it". – C1
	20. "If it's lower than 30 than I worry it may be significant. If its below 25 or 20 I'm fairly convinced it is significant".- C1
	21. "…one really has to have experience as well as the clinical oversite of the patient to take it in context…whether or not what we're seeing is really the reason why the patient is sick….especially for those borderline Ct values when you have them between 25 and 32. You know when you have it (..) although it is below 30 it's not quite (.) very low and it's 27 and 28 and whether or not I want to add specific antibiotic to cover that pathogen. It's something which is difficult at times".- C3

Table S10 cont.

Confidence in interpreting TAC grew throughout the study.	22.	"[We're] certainly a lot more comfortable than when we started and when you started doing the study". – C3
	23.	"…some consultants were more comfortable … with looking at the results that were given by TAC and coming to the conclusion that this was likely a commensal organism that actually was not necessarily pathogenic in this particular case, Other consultants were less happy with that and…would more readily continue antimicrobial therapy or broaden antimicrobial therapy based on the results…".– R1
	24.	"I think our interpretation of it is getting better". – C4
Decision making relating to TAC findings was made within the multi-disciplinary team.	25.	"…we have microbiology backup, we would just run it by them as a second opinion.." - C2
	26.	"…we make a joint decision whether we're escalating antibiotics or antifungals or whether we're in a situation where we can take them away".- C3
	27.	"…the useful thing is that you have a collective knowledge and on top of that you have the collective knowledge of everyone interpreting the test. You have a big clinical team, all interpreting the test, you have input from microbiology". – C4

C: Consultant (senior doctor); CRP: C-reactive protein; Ct: cycle threshold; NPA: nasopharyngeal aspirate; N: Nurse; PICU: Paediatric intensive care unit; R: Registrar (senior doctor-in-training); TAC: TaqMan array card

Table S11 Future research recommendations – Supporting quotations of thematic analysis

Theme	Supporting quotations
Ensuring the format in which TAC results are reported is easy to follow could help avoid test misinterpretation.	1. "…in Epic because it could be really easy to see something and say it's positive and therefore it's significant without going down and looking whether it is or not. So initially I didn't really understand you know, the full set of information that we were being given and what it meant. But, you know, after a couple of weeks or whatever a few of these being put in front of you I think it's easy enough. But I guess if there was a way to make that slightly more idiot proof it would probably be good". – R4
A study with healthy controls could help determine the significance of detections on TAC in children with suspected LRTI.	2. "You could sample well people and see what you get up from that. But I think from our point of view the reason they've been selected in the first place is because they've got a respiratory issue and so then I would be inclined if they had a significant Ct value to take it at face value, that's the cause of the problem most of the time". – R4
TAC could be used for both screening and diagnostic purposes. Most staff felt TAC should be used in a sub-set of patients where the test is likely to be highest yield.	3. "I think it's useful to have it there as an option to do, I don't think we should be doing it on everyone". – N2
	4. "You might just have to make sure it's not for everyone or something. A separate criteria. There's probably no point or your traumatic brain injury patient but in wintertime for your respiratory patient or those where you're not sure what's going on and you've looked at all other avenues". – N3
	5. "…it has great uses when the pre-test probability of a lower respiratory tract infection is high". – R1
	6. "It may become a routine part of testing. You know, we have to be careful of that. I think we probably have to ration it's use and make it cost effective and not just do it for everybody".- C4
	7. "…its cost effectiveness, it's diagnostic yield, the difference it being a diagnostic test or a screening test. Whether it fills the criteria for both". – C4
	8. "I would expect from an ITU (intensive care unit) point of view it would become part of our admission process. Normally when a ventilated patient comes in you do an ETT sample of secretions as well as an NPA or something. But it may well be that TAC would become part of that". – N3
	9. "An issue that I had with those particular patients, was that perhaps that these tests were ordered perhaps without necessarily thinking about where the pathology might be".- R1
An economic assessment needs to take place to understand the role of TAC.	10. "It needs to be taken with a broader context of cost in a publicly funded system, however. If money was no object, absolutely yes. If it costs a lot to make those decisions slightly more accurate, then maybe not". - C5

C: Consultant (senior doctor); Ct: cycle threshold; ETT: endotracheal tube; LRTI: lower respiratory tract infection; NPA: nasopharyngeal aspirate; N: Nurse; PICU: Paediatric intensive care unit; R: Registrar (senior doctor-in-training); TAC: TaqMan array card
