# Supplementary material for: A qualitative investigation of paediatric intensive care staff attitudes towards the diagnosis of lower respiratory tract infection in the molecular diagnostics era
Source: Intensive Care Med Paediatr Neonatal. 2023 Jul 7;1(1):10. doi: 10.1007/s44253-023-00008-z (PMC10329081; doi:10.1007/s44253-023-00008-z)
Supplement: Supplementary file 2 — Additional file 2: PRAMS survey. [file 44253_2023_8_MOESM2_ESM.pdf]

Paediatric Respiratory Antimicrobial decisions Survey

Thank you for considering taking part in PRAMS - the Paediatric Respiratory Antimicrobial decision Survey. This project aims to help us understand what underlies antimicrobial decision making in PICU for lower respiratory tract infection. We estimate that the survey will take approximately 10 minutes to complete. The full participant information is below – by continuing to answer the survey questions we will assume you have understood this information and provide your consent to participate.

Thank you for providing your valuable time,

Dr John Clark

PICU Fellow/PhD Student

On behalf of the study team

PARTICIPANT INFORMATION

PRAMS: Paediatric Respiratory Antimicrobial decision Making Survey

Introduction: Before you decide to take part in this study it is important for you to understand why the research is being done and what it will involve. A member of the team can be contacted if there is anything that is not clear or if you would like more information.

Purpose of the study: This project is a survey that will be distributed to staff that work in paediatric intensive care units in Europe, the UK, Australia, and New Zealand. It is investigating how healthcare professionals make decisions to start, change and stop antibiotics in critically unwell children with lung infection. There is limited information about how healthcare professionals use a patient's physical signs and hospital tests to make these decisions. This information is of interest as there is a global drive to reduce unnecessary use of antibiotics, given this can contribute to the development of antibiotic resistant bugs.

Why have I been chosen? You have been approached as a prescriber in a paediatric intensive care unit in Australia, Europe, New Zealand, or the United Kingdom.

Do I have to take part? Participation is entirely voluntary. Withdrawal is possible any time prior to the end of the survey collection period, by contacting the study team.

What will happen to me if I take part? You will be asked to complete a 10-minute online survey, which follows this participant information sheet.

What do I have to do? You will answer a range of multiple choice and Likert scale (rating least to most highly applicable) questions relating to how you make antimicrobial decisions in the PICU.

Are there possible disadvantages and/or risks in taking part? There are no foreseeable risks related to this study, we are not collecting identifiable information.

What are the possible benefits of taking part? There are no direct benefits to taking part in this study, however it will help our understanding of how antimicrobial decisions are made. This evidence will assist research development for quality improvement projects in PICUs internationally.

Will my taking part in this project be kept confidential? No personal data will be collected as part of this project, ensuring your information will remain confidential. We will request the name of your hospital, which will be de-identified by the study team. This will allow data to be matched to regions, to understand broad variations in practice. This data will be kept secure within the University's electronic data management system.

What will happen to the results of the research project? On completion of the study, the data will be made available via an open access framework, as required by peer reviewed journals. This open access data will not include the names of participant's hospitals. We will share the study findings in peer reviewed medical journals and international conferences.

Who is organising and funding the research? The research is being supported by the Department of Paediatrics at the University of Cambridge. Support for survey distribution will be sought via the European Society of Paediatric and Neonatal Intensive Care (ESPNIC), Paediatric Critical Care Society (PCCS) and The Australian and New Zealand Intensive Care Society – Paediatric Study Group (ANZICS-PSG). There is no direct funding for this project.

Ethical review of the study: The project has been reviewed by the University of Cambridge Psychology Research Ethics Committee.

Contact for further information: For any queries, please contact Dr John Clark jac302@cam.ac.uk Department of Paediatrics, University of Cambridge, Level 8, Addenbrooke’s Hospital, Cambridge Biomedical Campus, Cambridge CB2 0QQ United Kingdom

1a) Which country is your PICU located in?

◯ Afghanistan
◯ Albania
◯ Algeria
◯ Andorra
◯ Angola
◯ Antigua and Barbuda
◯ Argentina
◯ Armenia
◯ Australia
◯ Austria
◯ Azerbaijan
◯ Bahamas
◯ Bahrain
◯ Bangladesh
◯ Barbados
◯ Belarus
◯ Belgium
◯ Belize
◯ Benin
◯ Bhutan
◯ Bolivia
◯ Bosnia and Herzegovina
◯ Botswana
◯ Brazil
◯ Brunei
◯ Bulgaria
◯ Burkina Faso
◯ Burundi
◯ Côte d'Ivoire
◯ Cabo Verde
◯ Cambodia
◯ Cameroon
◯ Canada
◯ Central African Republic
◯ Chad
◯ Chile
◯ China
◯ Colombia
◯ Comoros
◯ Congo (Congo-Brazzaville)
◯ Costa Rica
◯ Croatia
◯ Cuba
◯ Cyprus
◯ Czechia (Czech Republic)
◯ Democratic Republic of the Congo
◯ Denmark
◯ Djibouti
◯ Dominica
◯ Dominican Republic
◯ Ecuador
◯ Egypt
◯ El Salvador
◯ Equatorial Guinea
◯ Eritrea
◯ Estonia
◯ Eswatini (fmr. "Swaziland")
◯ Ethiopia
◯ Fiji
◯ Finland
◯ France
◯ Gabon
◯ Gambia
◯ Georgia
◯ Germany
◯ Ghana
◯ Greece
◯ Grenada
◯ Guatemala

◯ Guinea
◯ Guinea-Bissau
◯ Guyana
◯ Haiti
◯ Holy See
◯ Honduras
◯ Hungary
◯ Iceland
◯ India
◯ Indonesia
◯ Iran
◯ Iraq
◯ Ireland
◯ Israel
◯ Italy
◯ Jamaica
◯ Japan
◯ Jordan
◯ Kazakhstan
◯ Kenya
◯ Kiribati
◯ Kuwait
◯ Kyrgyzstan
◯ Laos
◯ Latvia
◯ Lebanon
◯ Lesotho
◯ Liberia
◯ Libya
◯ Liechtenstein
◯ Lithuania
◯ Luxembourg
◯ Madagascar
◯ Malawi
◯ Malaysia
◯ Maldives
◯ Mali
◯ Malta
◯ Marshall Islands
◯ Mauritania
◯ Mauritius
◯ Mexico
◯ Micronesia
◯ Moldova
◯ Monaco
◯ Mongolia
◯ Montenegro
◯ Morocco
◯ Mozambique
◯ Myanmar (formerly Burma)
◯ Namibia
◯ Nauru
◯ Nepal
◯ Netherlands
◯ New Zealand
◯ Nicaragua
◯ Niger
◯ Nigeria
◯ North Korea
◯ North Macedonia
◯ Norway
◯ Oman
◯ Pakistan
◯ Palau
◯ Palestine State
◯ Panama
◯ Papua New Guinea
◯ Paraguay
◯ Peru
◯ Philippines
◯ Poland

◯ Portugal
◯ Qatar
◯ Romania
◯ Russia
◯ Rwanda
◯ Saint Kitts and Nevis
◯ Saint Lucia
◯ Saint Vincent and the Grenadines
◯ Samoa
◯ San Marino
◯ Sao Tome and Principe
◯ Saudi Arabia
◯ Senegal
◯ Serbia
◯ Seychelles
◯ Sierra Leone
◯ Singapore
◯ Slovakia
◯ Slovenia
◯ Solomon Islands
◯ Somalia
◯ South Africa
◯ South Korea
◯ South Sudan
◯ Spain
◯ Sri Lanka
◯ Sudan
◯ Suriname
◯ Sweden
◯ Switzerland
◯ Syria
◯ Tajikistan
◯ Tanzania
◯ Thailand
◯ Timor-Leste
◯ Togo
◯ Tonga
◯ Trinidad and Tobago
◯ Tunisia
◯ Turkey
◯ Turkmenistan
◯ Tuvalu
◯ Uganda
◯ Ukraine
◯ United Arab Emirates
◯ United Kingdom
◯ United States of America
◯ Uruguay
◯ Uzbekistan
◯ Vanuatu
◯ Venezuela
◯ Vietnam
◯ Yemen
◯ Zambia
◯ Zimbabwe

1b) What is the name of your hospital?

____

2) What services does your PICU provide?
Tick all applicable options

☐ Cardiac intensive care
☐ General intensive care
☐ General surgical intensive care
☐ Neurosurgical intensive care
☐ Integrated adult/paediatric intensive care

3) What is your role in the intensive care unit?

◯ Consultant/attending
◯ Senior medical trainee (completed primary examinations of relevant post-graduate college)
◯ Junior medical trainee (yet to undertake postgraduate specialty examinations)
◯ Advanced nurse practitioner
◯ Nurse practitioner

Section B: Scenario

Sections B-D include case scenarios to understand
1. How PICU prescribers use information at the bedside to make antimicrobial decisions for community acquired pneumonia (CAP) and ventilator associated pneumonia (VAP); and
2. How PICU prescribers use investigations to support antimicrobial decision making for CAP and VAP.

Section B scenario: A boy receiving mechanical ventilation requires admission to your PICU. He has features suggestive of community acquired pneumonia.

4a) Select the factor/s that you consider relevant in making a decision whether to prescribe or continue antimicrobial therapy.

☐ Age of the patient
☐ History of chronic respiratory disease
☐ History of chronic (non-respiratory) medical problem
☐ Immunosuppression - due to disease status or medication
☐ Known colonisation of the respiratory tract
☐ Known/previous antimicrobial resistance,
☐ Tracheostomy +/- home ventilation
☐ Escalation of respiratory support eg. High flow to CPAP
☐ Fever
☐ Findings on auscultation
☐ New cough
☐ New/increased production of respiratory secretions
☐ Oxygen requirement
☐ Ventilator pressure requirements
☐ Respiratory rate (prior to intubation),
☐ Other feature on history or examination
☐ None of the above

4b) Of the factor/s you have highlighted, rate their importance in your decision to commence/continue antimicrobial therapy.
You can place the slide bar anywhere on the range from not important to highly important.

Age of the patient — Not important ——— Highly important

(Place a mark on the scale above)

History of chronic respiratory disease — Not important ——— Highly important

(Place a mark on the scale above)

History of chronic (non-respiratory) medical problem — Not important ——— Highly important

(Place a mark on the scale above)

Immunosuppression - due to disease status or medication — Not important ——— Highly important

(Place a mark on the scale above)

Known colonisation of the respiratory tract — Not important ——— Highly important

(Place a mark on the scale above)

Known/previous antimicrobial resistance — Not important ——— Highly important

(Place a mark on the scale above)

Tracheostomy +/- home ventilation	Not important — Highly important<br>(Place a mark on the scale above)
Escalation of respiratory support eg. high flow to CPAP	Not important — Highly important<br>(Place a mark on the scale above)
Fever	Not important — Highly important<br>(Place a mark on the scale above)
Findings on auscultation	Not important — Highly important<br>(Place a mark on the scale above)
New cough	Not important — Highly important<br>(Place a mark on the scale above)
New/increased production of respiratory secretions	Not important — Highly important<br>(Place a mark on the scale above)
Oxygen requirement	Not important — Highly important<br>(Place a mark on the scale above)
Ventilator pressure requirements	Not important — Highly important<br>(Place a mark on the scale above)
Respiratory rate (prior to intubation)	Not important — Highly important<br>(Place a mark on the scale above)

You selected that you would use other features on history/examination to aid your decision making to prescribe antimicrobial therapy in this patient.

In the following free text box please -
a) Describe what additional features you consider important on history or examination.
b) Rate each of these features out of 100 in their importance (0 = not important, 100 = highly important).

____

4c) Which of the following investigations (if any) would you routinely request to investigate this patient's pneumonia?

- ☐ Chest x-ray
- ☐ Chest ultrasound
- ☐ Blood gas (venous/capillary/arterial)
- ☐ Full blood count
- ☐ C-Reactive protein
- ☐ Procalcitonin
- ☐ Multi-pathogen array - endotracheal aspirate
- ☐ Multi-pathogen array - non-bronchoscopic bronchoalveolar lavage
- ☐ Multi-pathogen array - broncho-alveolar lavage
- ☐ Culture - blood
- ☐ Culture - endotracheal aspirate
- ☐ Culture - non-bronchoscopic bronchoalveolar lavage
- ☐ Culture - bronchoscopic bronchoalveolar lavage
- ☐ Viral respiratory PCR panel - endotracheal aspirate
- ☐ Viral respiratory PCR panel - nasopharyngeal aspirate
- ☐ Viral respiratory PCR panel - nasopharyngeal swab
- ☐ Viral respiratory PCR panel - non-bronchoscopic bronchoalveolar lavage
- ☐ Viral respiratory PCR panel - bronchoscopic bronchoalveolar lavage
- ☐ 16S/18S ribosomal RNA sequencing- blood
- ☐ 16S/18S ribosomal RNA sequencing- respiratory sample
- ☐ Urinary pneumonia antigens - eg. S pneumoniae, pneumococcal, legionella antigen
- ☐ Other investigation
- ☐ None of the above

4d) Of the investigations you selected, rate their usefulness in making your decision whether to commence/continue antimicrobial therapy in this patient.

You can place the slide bar anywhere on the range from not useful to extremely useful

---

Chest x-ray

Not useful — Extremely useful

(Place a mark on the scale above)

Chest ultrasound

Not useful — Extremely useful

(Place a mark on the scale above)

Blood gas (venous/capillary/arterial)

Not useful — Extremely useful

(Place a mark on the scale above)

Full blood count

Not useful — Extremely useful

(Place a mark on the scale above)

C-Reactive protein

Not useful — Extremely useful

(Place a mark on the scale above)

Procalcitonin

Not useful — Extremely useful

(Place a mark on the scale above)

Multi-pathogen array - endotracheal aspirate

Not useful — Extremely useful

(Place a mark on the scale above)

Multi-pathogen array - non-bronchoscopic bronchoalveolar lavage

Not useful Extremely useful

(Place a mark on the scale above)

Multi-pathogen array - bronchoscopic bronchoalveolar lavage

Not useful Extremely useful

(Place a mark on the scale above)

Culture - blood

Not useful Extremely useful

(Place a mark on the scale above)

Culture - endotracheal aspirate

Not useful Extremely useful

(Place a mark on the scale above)

Culture - non-bronschoscopic bronchoalveolar lavage

Not useful Extremely useful

(Place a mark on the scale above)

Culture - bronchoscopic bronchoalveolar lavage

Not useful Extremely useful

(Place a mark on the scale above)

Viral respiratory panel - endotracheal aspirate

Not useful Extremely useful

(Place a mark on the scale above)

Viral respiratory PCR panel - Nasopharyngeal aspirate (NPA)

Not useful Extremely useful

(Place a mark on the scale above)

Viral respiratory PCR panel - Nasopharyngeal swab

Not useful Extremely useful

(Place a mark on the scale above)

Viral respiratory PCR panel - Non-bronchoscopic bronchoalveolar lavage (NB-BAL)

Not useful Extremely useful

(Place a mark on the scale above)

Viral respiratory PCR panel - Bronchoscopic bronchoalveolar lavage (BAL)

Not useful Extremely useful

(Place a mark on the scale above)

16S/18S ribosomal RNA sequencing - blood

Not useful Extremely useful

(Place a mark on the scale above)

16S/18S - ribosomal RNA sequencing - respiratory sample

Not useful Extremely useful

(Place a mark on the scale above)

Urinary pneumonia antigens - eg. S pneumoniae, pneumococcal, legionella antigen

Not useful Extremely useful

(Place a mark on the scale above)

You have stated you would request an 'other' investigation.
In the following free text box please -
a) Describe what 'other' investigation/s you would order
b) Rate each of these investigation/s out of 100 in their importance (0 = not important, 100 = highly important).

____

Section C: Scenario

A five year old girl has been ventilated on the PICU for several days. She has not yet received any antimicrobial therapy. A member of the nursing team states they are concerned that the child may have ventilator associated pneumonia (VAP).

5a) Of the following clinical factors, select all those that you would consider relevant in your decision whether to commence antimicrobial therapy.

- ☐ Chronic respiratory disease
- ☐ Chronic underlying medical condition
- ☐ Immunosuppression - by disease status or medication
- ☐ Known colonisation of the respiratory tract
- ☐ Known/previous antimicrobial resistance
- ☐ Duration of mechanical ventilation
- ☐ Increased ventilatory requirements
- ☐ Increased oxygen requirement
- ☐ Quality or quantity of secretions
- ☐ Fever
- ☐ Findings on auscultation
- ☐ Respiratory rate (if not paralysed)
- ☐ Generalised clinical deterioration
- ☐ Other
- ☐ None of the above

5b) Of the factors you selected, rate them in their importance in your decision to commence antimicrobial therapy

Chronic respiratory disease

Not important Highly important

(Place a mark on the scale above)

Chronic underlying medical condition

Not important Highly important

(Place a mark on the scale above)

Immunosuppression - by disease status or medication

Not important Highly important

(Place a mark on the scale above)

Known colonisation of the respiratory tract

Not important Highly important

(Place a mark on the scale above)

Known/previous antimicrobial resistance

Not important Highly important

(Place a mark on the scale above)

Duration of mechanical ventilation

Not important Highly important

(Place a mark on the scale above)

Increased ventilatory requirements

Not important Highly important

(Place a mark on the scale above)

Increased oxygen requirement

Not important Highly important

(Place a mark on the scale above)

Quantity/quality of secretions	Not important Highly important<br>(Place a mark on the scale above)
Fever	Not important Highly important<br>(Place a mark on the scale above)
Findings on auscultation	Not important Highly important<br>(Place a mark on the scale above)
Respiratory rate (if not paralysed)	Not important Highly important<br>(Place a mark on the scale above)
General clinical deterioration	Not important Highly important<br>(Place a mark on the scale above)
You stated you would consider 'other' clinical parameters in your decision to start antimicrobial therapy in this patient.<br>In the following free text box please -<br>a) Describe what additional features you consider important.<br>b) Rate each of these features out of 100 in their importance (0 = not important, 100 = highly important).	__________________________________

5c) Which of the following investigations would you request to investigate this patient for ventilator associated pneumonia?

☐ Chest x-ray
☐ Chest ultrasound
☐ Blood gas (venous/capillary/arterial)
☐ Full blood count
☐ C-Reactive protein
☐ Procalcitonin
☐ Multi-pathogen array - endotracheal aspirate
☐ Multi-pathogen array - non-bronchoscopic bronchoalveolar lavage
☐ Multi-pathogen array - broncho-alveolar lavage
☐ Culture - blood
☐ Culture - endotracheal aspirate
☐ Culture - non-bronchoscopic bronchoalveolar lavage
☐ Culture - bronchoscopic bronchoalveolar lavage
☐ Viral respiratory PCR panel - endotracheal aspirate
☐ Viral respiratory PCR panel - nasopharyngeal aspirate
☐ Viral respiratory PCR panel - nasopharyngeal swab
☐ Viral respiratory PCR panel - non-bronchoscopic bronchoalveolar lavage
☐ Viral respiratory PCR panel - bronchoscopic bronchoalveolar lavage
☐ 16S/18S RNA ribosomal sequencing- blood
☐ 16S/18S RNA ribosomal sequencing- respiratory sample
☐ Urinary pneumonia antigens - eg. S pneumoniae, pneumococcal, legionella antigen
☐ Other investigation
☐ None of the above

5d) Of the investigations you selected, rate their usefulness in making your decision whether to commence antimicrobial therapy for ventilator associated pneumonia

You can place the slide bar anywhere on the range from not useful to extremely useful

Chest x-ray	Not useful	Extremely useful
		(Place a mark on the scale above)
Chest ultrasound	Not useful	Extremely useful
		(Place a mark on the scale above)
Blood gas (venous/capillary/arterial)	Not useful	Extremely useful
		(Place a mark on the scale above)
Full blood count	Not useful	Extremely useful
		(Place a mark on the scale above)
C-Reactive protein	Not useful	Extremely useful
		(Place a mark on the scale above)
Procalcitonin	Not useful	Extremely useful
		(Place a mark on the scale above)
Multi-pathogen array - endotracheal aspirate	Not useful	Extremely useful
		(Place a mark on the scale above)
Multi-pathogen array - non-bronchoscopic bronchoalveolar lavage	Not useful	Extremely useful
		(Place a mark on the scale above)
Multi-pathogen array - bronchoalveolar lavage (BAL)	Not useful	Extremely useful
		(Place a mark on the scale above)
Culture - blood	Not useful	Extremely useful
		(Place a mark on the scale above)
Culture - endotracheal aspirate	Not useful	Extremely useful
		(Place a mark on the scale above)
Culture - non-bronschoscopic bronchoalveolar lavage	Not useful	Extremely useful
		(Place a mark on the scale above)
Culture - bronchoscopic bronchoalveolar lavage	Not useful	Extremely useful
		(Place a mark on the scale above)
Viral respiratory panel - endotracheal aspirate	Not useful	Extremely useful
		(Place a mark on the scale above)

Viral respiratory panel - Nasopharyngeal aspirate (NPA)

Not useful | Extremely useful

(Place a mark on the scale above)

Viral respiratory PCR panel - Nasopharyngeal swab

Not useful | Extremely useful

(Place a mark on the scale above)

Viral respiratory PCR panel - Non-bronchoscopic bronchoalveolar lavage (NB-BAL)

Not useful | Extremely useful

(Place a mark on the scale above)

Viral respiratory PCR panel - Bronchoscopic bronchoalveolar lavage (BAL)

Not useful | Extremely useful

(Place a mark on the scale above)

16S/18S ribosomal RNA sequencing - blood

Not useful | Extremely useful

(Place a mark on the scale above)

16S/18S ribosomal RNA sequencing - respiratory sample

Not useful | Extremely useful

(Place a mark on the scale above)

Urinary pneumonia antigens - eg. S pneumoniae, pneumococcal, legionella antigen

Not useful | Extremely useful

(Place a mark on the scale above)

You have stated you would request an 'other' investigation.
In the following free text box please -
a) Describe what 'other' investigation/s you would order
b) Rate each of these investigation/s out of 100 in their importance (0 = not important, 100 = highly important).

____

Section D: Scenario

A patient on your PICU has received 5 days of first line antimicrobial therapy for severe community acquired pneumonia. One of your colleagues is concerned regarding treatment failure.

6a) Which of the following clinical features would be of most concern to you that antimicrobial therapy was failing to treat the pneumonia?

☐ Increased/persistently elevated ventilatory requirements
☐ Increased/persistently elevated oxygen requirement
☐ Quantity/quality of secretions
☐ Fever
☐ Findings on auscultation
☐ Haemodynamic instability
☐ Respiratory rate (if not paralysed)
☐ Other

Of the factors you selected, rate them in their importance.

Increased/persistently elevated ventilatory requirements

Not important — Highly important

(Place a mark on the scale above)

Increased/persistently elevated oxygen requirement

Not important — Highly important

(Place a mark on the scale above)

Quantity/quality of secretions

Not important — Highly important

(Place a mark on the scale above)

Fever

Not important — Highly important

(Place a mark on the scale above)

Findings on auscultation

Not important — Highly important

(Place a mark on the scale above)

Haemodynamic instability

Not important — Highly important

(Place a mark on the scale above)

Respiratory rate (if not paralysed)

Not important — Highly important

(Place a mark on the scale above)

You stated you would consider 'other' features in this patient.
In the following free text box please -
a) Describe what additional features you consider important.
b) Rate each of these features out of 100 in their importance (0 = not important, 100 = highly important).

____

6b) Which of the following investigations would you request (if any) to help determine whether escalation of antimicrobial therapy is required?

- [ ] Chest x-ray
- [ ] Chest ultrasound
- [ ] Blood gas (venous/capillary/arterial)
- [ ] Full blood count
- [ ] C-Reactive protein
- [ ] Procalcitonin
- [ ] Multi-pathogen array - endotracheal aspirate
- [ ] Multi-pathogen array - non-bronchoscopic bronchoalveolar lavage
- [ ] Multi-pathogen array - broncho-alveolar lavage
- [ ] Culture - blood
- [ ] Culture - endotracheal aspirate
- [ ] Culture - non-bronchoscopic bronchoalveolar lavage
- [ ] Culture - bronchoscopic bronchoalveolar lavage
- [ ] Viral respiratory PCR panel - endotracheal aspirate
- [ ] Viral respiratory PCR panel - nasopharyngeal aspirate
- [ ] Viral respiratory PCR panel - nasopharyngeal swab
- [ ] Viral respiratory PCR panel - non-bronchoscopic bronchoalveolar lavage
- [ ] Viral respiratory PCR panel - bronchoscopic bronchoalveolar lavage
- [ ] 16S/18S ribosomal RNA sequencing- blood
- [ ] 16S/18S ribosomal RNA sequencing- respiratory sample
- [ ] Urinary pneumonia antigens - eg. S pneumoniae, pneumococcal, legionella antigen
- [ ] Other investigation
- [ ] None of the above

6c) Rate the usefulness of these investigations in your decision to escalate antimicrobial therapy

Chest x-ray — Not useful / Extremely useful

(Place a mark on the scale above)

Chest ultrasound — Not useful / Extremely useful

(Place a mark on the scale above)

Blood gas (venous/capillary/arterial) — Not useful / Extremely useful

(Place a mark on the scale above)

Full blood count — Not useful / Extremely useful

(Place a mark on the scale above)

C-Reactive protein — Not useful / Extremely useful

(Place a mark on the scale above)

Procalcitonin — Not useful / Extremely useful

(Place a mark on the scale above)

Multi-pathogen array - endotracheal aspirate — Not useful / Extremely useful

(Place a mark on the scale above)

Multi-pathogen array - non-bronchoscopic bronchoalveolar lavage (NB-BAL) — Not useful / Extremely useful

(Place a mark on the scale above)

Multi-pathogen array - bronchoalveolar lavage (BAL)
Not useful — Extremely useful
(Place a mark on the scale above)

Culture - blood
Not useful — Extremely useful
(Place a mark on the scale above)

Culture - endotracheal aspirate
Not useful — Extremely useful
(Place a mark on the scale above)

Culture - non-bronschoscopic bronchoalveolar lavage (NB-BAL)
Not useful — Extremely useful
(Place a mark on the scale above)

Culture - bronchoscopic bronchoalveolar lavage
Not useful — Extremely useful
(Place a mark on the scale above)

Viral respiratory PCR panel - endotracheal aspirate
Not useful — Extremely useful
(Place a mark on the scale above)

Viral respiratory PCR panel - Nasopharyngeal aspirate (NPA)
Not useful — Extremely useful
(Place a mark on the scale above)

Viral respiratory PCR panel - Nasopharyngeal swab
Not useful — Extremely useful
(Place a mark on the scale above)

Viral respiratory PCR panel - Non-bronchoscopic bronchoalveolar lavage (NB-BAL)
Not useful — Extremely useful
(Place a mark on the scale above)

Viral respiratory PCR panel - Bronchoscopic bronchoalveolar lavage (BAL)
Not useful — Extremely useful
(Place a mark on the scale above)

16S/18S ribosomal RNA sequencing- blood
Not useful — Extremely useful
(Place a mark on the scale above)

16S/18S ribosomal RNA sequencing- respiratory sample
Not useful — Extremely useful
(Place a mark on the scale above)

Urinary pneumonia antigens - eg. S pneumoniae, pneumococcal, legionella antigen
Not useful — Extremely useful
(Place a mark on the scale above)

You have stated you would request an 'other' investigation.
In the following free text box please -
a) Describe what 'other' investigation/s you would order
b) Rate each of these investigation/s out of 100 in their importance (0 = not important, 100 = highly important).

Section E: Final questions

7a) Which of the following (if any) do you consider when deciding to cease antimicrobial therapy for severe lower respiratory tract infection?

- ☐ Clinical/physiological parameters
- ☐ Biochemical investigations
- ☐ Microbiology investigations
- ☐ Virology investigations eg. PCR
- ☐ Imaging investigations (chest x-ray or ultrasound)
- ☐ Pre-determined protocol/guideline based decision to cease
- ☐ None of the above

7b) Rate the importance of these factors in your decision making to cease antimicrobial therapy

Clinical/physiological parameters

Low importance — High importance

(Place a mark on the scale above)

Biochemical investigations

Low importance — High importance

(Place a mark on the scale above)

Microbiology investigations

Low importance — High importance

(Place a mark on the scale above)

Virology investigations

Low importance — High importance

(Place a mark on the scale above)

Imaging investigations

Low importance — High importance

(Place a mark on the scale above)

Pre-determined protocol/guideline based decision to cease

Low importance — High importance

(Place a mark on the scale above)

8) How confident are you in making an accurate diagnosis of ventilator associated pneumonia using your own clinical assessment and investigations available in your hospital?

Not confident at all — Highly confident

(Place a mark on the scale above)

9) Rate the following components of the Modified Clinical Pulmonary Infection Score* on their ability to accurately diagnose ventilator associated pneumonia, based on your experience

* Singh, N., Rogers, P., Atwood, C., Wagener, M. M. & Yy, V. L. Short-course Empiric Antibiotic Therapy for Patients with Pulmonary Infiltrates in the Intensive Care Unit. Am. J. Respir. Crit. Care Med. 162, 505–511 (2000)

Temperature outside normal range

Not relevant — Highly relevant

(Place a mark on the scale above)

Blood leukocytes outside normal range

Not relevant — Highly relevant

(Place a mark on the scale above)

Tracheal secretions - presence and quality	Not relevant — Highly relevant<br>(Place a mark on the scale above)
Oxygenation index	Not relevant — Highly relevant<br>(Place a mark on the scale above)
Infiltrate on chest x-ray	Not relevant — Highly relevant<br>(Place a mark on the scale above)
Radiographic progression of pulmonary infiltration	Not relevant — Highly relevant<br>(Place a mark on the scale above)
Growth of pathogenic bacteria on tracheal aspirate	Not relevant — Highly relevant<br>(Place a mark on the scale above)

10) If a 'perfect' test (high sensitivity, high specificity) was available, how long (in hours) would you be willing to wait for investigation results prior to commencing antimicrobial therapy in the following patients?
Please respond in hours eg. 1 day = 24, 3 days = 72

A stable patient requiring mechanical ventilation admitted to the PICU with community acquired pneumonia (hours) ________________________

An unstable patient requiring mechanical ventilation admitted to the PICU with community acquired pneumonia (hours) ________________________

A stable patient with ventilator associated pneumonia (hours) ________________________

An ustable patient with ventilator associated pneumonia (hours) ________________________

These final questions are optional, based on your access to clinical data

11a) How many ventilated patients are currently receiving mechanical ventilation in your PICU? (optional) ______________________

11b) How many ventilated patients are receiving systemic antimicrobial therapy? (optional) ______________________

11c) How many ventilated patients are receiving treatment for community acquired pneumonia? (optional) ______________________

11d) How many patients are receiving treatment for ventilator associated pneumonia? (optional) ______________________

11e) How many of the patients with ventilator associated pneumonia have had their infection confirmed on microbiology tests? (optional) ______________________

If you have any further comments you would like to add about this survey, diagnosis and investigations for pneumonia in the PICU please write them here ____________________________
