# Supplementary material for: A qualitative investigation of paediatric intensive care staff attitudes towards the diagnosis of lower respiratory tract infection in the molecular diagnostics era
Source: Intensive Care Med Paediatr Neonatal. 2023 Jul 7;1(1):10. doi: 10.1007/s44253-023-00008-z (PMC10329081; doi:10.1007/s44253-023-00008-z)
Supplement: Supplementary file 3 — Additional file 3: RASCALS interview guide. [file 44253_2023_8_MOESM3_ESM.docx]

**
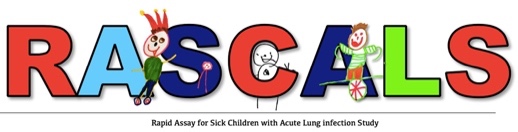
Rapid Assay for Sick Children with Acute Lung infection Study**

**Semi-structured interview guide**

REC number: A095474

IRAS number: 277039

Protocol version number 8.0, 31/05/2021

**Interview approach**: Participants will be sampled opportunistically (convenience sampling) from the PICU and clinical microbiology teams. This is to ensure that there is minimal disruption to the clinical responsibilities of staff, and that interviews occur at times when there is adequate staffing. Only staff that are familiar with the RASCALS will be selected to participate – as some staff may be from agencies or have rotated from other hospitals hence not been exposed to the study. A funnelled protocol will be used. Interviews will continue until thematic saturation is reached.

| **Interview component** | **Content and questions** |
| --- | --- |
| 1. Welcome (1min) | Thank you for agreeing to meet with us. I am sure several of us know each other already – I’m John, a paediatrician at CUH and a PhD student at Cambridge University. I have been coordinating RASCALS on the unit under the supervision of Dr Nazima Pathan, PICU consultant and Prof Stephen Baker, microbiologist.  The core of RASCALS related to implementation of a custom diagnostic microarray card on this unit for children with suspected respiratory infection. We have completed testing of the card after enrolling 100 children to the project. We are now gathering data relating to the staff experience of this diagnostic test to help us understand its real-world application. These semi-structured interviews have been approved via a human ethics committee and is a component of RASCALS jointly sponsored by CUH NHS FT and the University of Cambridge. |
| 1. Ground rules (1min) | This interview will take up to a maximum of 30 minutes. All your responses are confidential in this session. Whilst we will obtain your name and role for the purposes of consent, this will be anonymised when we go on to complete data analysis and report our findings.  This session will be recorded, however only the research team will have access to these recordings. These will be destroyed after they are transcribed and anonymised.  We expect our findings to be published in a peer-review journal and this may include direct quotes from these interviews. These quotes will be anonymised, listing the individual’s role within PICU only eg. Senior nurse, registrar.  Are there any questions relating to your participation in the project? |
| 1. Introductions (1min) | I’m going to start the recording now, is that okay?”  (Recording starts)  Can you please provide your name and role? |
| 1. Group discussion – topic 1: Application (8min) | **Topic 1: Application**   1. Describe your experience of TaqMan array card (TAC) on the PICU    1. PROBE: Can you describe times where you requested a TAC?    2. PROBE: What was your opinion on the use of TAC on our PICU? 2. Can you describe if you used TAC in the management of your patients? If yes, how so? 3. Were there any situations in which TAC was helpful?    1. PROBE: Can you provide an example of when this was the case?    2. PROBE: Were there any times one of your patients had a TAC where it aided their management? 4. Were there any situations where TAC was problematic?    1. PROBE: Can you provide an example of when this was the case?    2. PROBE: Were there any times where TAC resulted in confusion or uncertainty in patient management? 5. In what circumstances, if any, would you consider requesting TAC if it were routinely available?    1. PROBE: Are there any specific situations where you feel a TAC is beneficial? |
| 1. Group discussion – topic 2: Interpretation (8min) | **Topic 2: Interpretation**   1. Can you describe how you interpret a TAC?    1. PROBE: TAC results were reported with the name of a bacterial, viral, or fungal target and a corresponding cycle threshold value. Can you advise how you used this information? 2. How confident were you in interpreting TAC results?    1. PROBE: When TAC results became available did you feel that you were able to act on the results with certainty? 3. Did your confidence in interpreting TAC results change throughout the course of the study? *If yes:* Can you tell me about that?    1. Did you feel more confident after you had reviewed more TAC results? 4. Did you interpret results on your own or rely on others to interpret the test?    1. PROBE: Did you rely on the input of others, such as the microbiology, or respiratory team to understand what the test results meant? 5. How did you use TAC alongside existing diagnostic methods?    1. PROBE: How did you use TAC results alongside routine tests such as microbiology cultures and biochemical tests? |
| 1. Group discussion – topic 3: Test in context (6min) | **Topic 3: Test in context**   1. Did you change patient management based on TAC results?    1. PROBE: Did you stop, start, or change antibiotics based on TAC results? 2. Were there any factors relating to sample collection you feel had an impact on TAC performance? 3. Is TAC a reliable test?    1. PROBE: Where would you rate TAC on a scale of 0 to 10 (10 being the best in terms of reliability? Why did you give it that rating?    2. PROBE: How reliable do you think TAC is in identifying micro-organisms causing respiratory infection? 4. Would you recommend that TAC is embedded into routine clinical practice? |
| 1. Final thoughts (2 min) | Do you have any final thoughts you wanted to share? |
| 1. Conclusion (1min) | Thank you for participating in the interview. If you have any questions relating to the project, or decide you wish to withdraw your consent from the project, please contact us using the details on the participant information sheet. If you know others that may agree to take part please let us know. |
