# Supplementary material for: A qualitative investigation of paediatric intensive care staff attitudes towards the diagnosis of lower respiratory tract infection in the molecular diagnostics era
Source: Intensive Care Med Paediatr Neonatal. 2023 Jul 7;1(1):10. doi: 10.1007/s44253-023-00008-z (PMC10329081; doi:10.1007/s44253-023-00008-z)
Supplement: Supplementary file 4 — Additional file 4: Participant consent form. [file 44253_2023_8_MOESM4_ESM.docx]

**Addenbrooke’s Hospital**

Hills Road

Cambridge CB2 0QQ

Switchboard: 01223 245151

[www.addenbrookes.org.uk](http://www.addenbrookes.org.uk/)

**Department of Paediatric Intensive Care**

Dr Roddy O’Donnell

Dr Shruti Agrawal

Dr David Inwald

Dr Riaz Kayani

Dr Girish Neelegowda

Dr Nazima Pathan

Dr Stewart Reid

Dr Francesc Torres


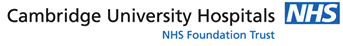


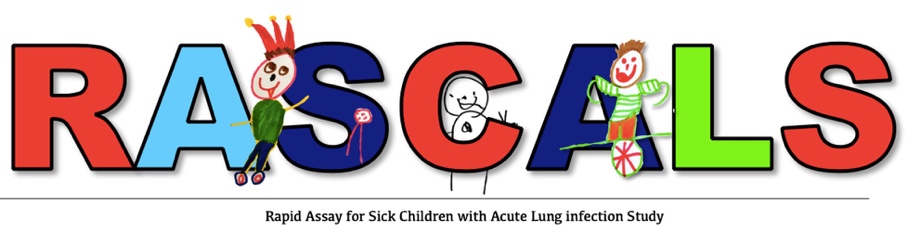


**Rapid Assay for Sick Children with Acute Lung infection Study**

**Staff focus group – experiences of TaqMan diagnostic array**

Thank you for taking the time to consider participating in the focus group for ‘RASCALS’ – the Rapid Assay for Sick Children with Acute Lung infection Study and reading the information sheets.

If you would like to participate in the focus group, please read and sign the following forms.

We will provide you with one copy of this form and retain a second copy securely in our research department at the hospital.

Kind regards,

**The Paediatric Intensive Care Unit Research Team**

RASCALS researchers: Dr Nazima Pathan, Dr John Clark, Dr Iain Kean, Dr Estée Török, Prof Gordon Dougan, Prof Stephen Baker, Dr Vilas Navapurkar, Ms Esther Daubney & Ms Deborah White.

Phone: 01223 336883

Email: [np409@medschl.cam.ac.uk](mailto:np409@medschl.cam.ac.uk)

| **Participant identification number**: | | **Initials** |
| --- | --- | --- |
| 1. | I have read the information sheet on this project, dated ___/___/____ (Version________) and have been given a copy to keep. I have been able to ask questions about the project and I understand why the research is being done and any risks involved. |  |
| 2. | I know how to contact the research team if I need to, and how to get information about the results of the research. |  |
| 3. | I understand that the focus group interview will be recorded, and this data will be transcribed with participant identity anonymised by the research team. |  |
| 4 | I understand that direct quotes from the deidentified interview transcript may be included in published research findings. |  |
| 5. | I understand that any information collected as part of this study will be stored securely in line with current NHS and University guidelines. I understand that only members of the clinical project team will have access to my identifiable information; all others analysing my data will only have access to anonymised data. I understand that my data will be retained for 15 years. |  |
| 6. | I understand that the information collected may be used to support other research in the future, and may be shared anonymously with other academic and commercial researchers external to the project within the UK and beyond |  |
| 7. | I consent to taking part in this study |  |

| **Written consent**  **Participant**  Name (Capitals):_____________________________________________________________________________  Signature:___________________________________________________ Date: dd/mm/yyyy |
| --- |
| **Person obtaining consent**  Name (Capitals):_____________________________________________________________________________  Signature:___________________________________________________ Date: dd/mm/yyyy  Role: _________________________________________________ |

| **Electronic consent**  Participant name (Capitals):_________________________________________________________________________ |
| --- |
| **Person obtaining consent**  Name (Capitals):_____________________________________________________________________________  Signature:___________________________________________________ Date: dd/mm/yyyy  Role: _________________________________________________ |

| **Verbal consent**  **Participant name** (Capitals):________________________________________________________________________ |
| --- |
| **Person obtaining consent**  Name (Capitals):_____________________________________________________________________________  Signature:___________________________________________________ Date: dd/mm/yyyy  Role: _________________________________________________  **Witness to verbal consent**  Name (Capitals):_____________________________________________________________________________  Signature:___________________________________________________ Date: dd/mm/yyyy  Role: _________________________________________________  **Primary investigator countersign for electronic and unwitnessed verbal consent**  **Dr Nazima Pathan** _________________________________________________ Date: dd/mm/yyyy |
