# Supplementary material for: A qualitative investigation of paediatric intensive care staff attitudes towards the diagnosis of lower respiratory tract infection in the molecular diagnostics era
Source: Intensive Care Med Paediatr Neonatal. 2023 Jul 7;1(1):10. doi: 10.1007/s44253-023-00008-z (PMC10329081; doi:10.1007/s44253-023-00008-z)
Supplement: Supplementary file 6 — Additional file 6: Raw data. [file 44253_2023_8_MOESM6_ESM.zip › All transcriptions combined.docx]

**Transcribed interviews - A qualitative investigation of paediatric intensive care staff attitudes towards the diagnosis of lower respiratory tract infection in the molecular diagnostics era**

These interviews were recorded between January –April 2022 at Cambridge University Hospitals NHS FT. They have been anonymised and categorised according to job role.

Acronyms in the transcription

COVID – Coronavirus disease 2019

CRP – C reactive protein

Ct – Cycle threshold

PICU – Paediatric intensive care unit

RSV – *Respiratory syncytial virus*

TAC – TaqMan array card

The participants are noted as

C_: Consultant (senior doctor)

JC: John Clark (interviewer)

N_: Nurse

R_: Registrar (senior doctor in training)

Date: 31/1/2022

Interview with R1

Interview duration: 3:50 + 14:10 = 18:00

Interview location: PICU doctors’ office

JC: Can you please give your name and role?

R1: [R1] PICU registrar

JC: Can you describe your experience, to me, of using TAC on PICU?

R1: As in requesting TAC (..) or (..)?

JC: (nods)

R1: The TAC was used for patients who were ventilated, who we were trying to identify what bugs may be causing their symptoms (..) It gave a respiratory viral panel and a bacterial panel. As well with bacteria that were identified and viruses that were identified it gave a Ct count (..) so gave, as far as my understanding was (..) the number of times the DNA had to be cycled through in order to identify (..) So a high count suggested these were just tiny particles of DNA that were not of any real significance and a low count suggested that there was significant DNA present there that didn’t require much replication in order to find so was much more significant as a result.

JC: Mm, okay, and what was your opinion on the use of TAC in our PICU?

R1: Umm, so (..) it was very useful (..) I (..) have had previous experience with viral respiratory microarrays in terms of trying to assess for patients that have viruses where we may be able to step antibiotics down. In terms of the bacteria, it was useful information (..) I don’t know enough about Ct values to say whether or not it (..) if we were able to fully (..) quantify the significance of a result essentially, so we had quite hard cut-offs of above 30 was insignificant, 25-30 was intermediately significant and less than 25 was (.) I may have got those numbers wrong but (.) I wasn’t (..) I don’t know where those numbers came from therefore it was more difficult for me sometimes to say how significant the result really was in that patient. For several patients it did allow us to identify pathogens significantly more quickly than the standard microbiological deep suctioning and culture and sensitives and the standard microbiological way. So, for some patients it was really good so we could narrow antibiotics down and give a much more targeted antibiotic regime. There were some patients where we (..) got both viruses and bacteria and some of those bacteria may or may not have had significance, but because we had a positive result, they may have ended up on a longer course of antibiotics than they would have done otherwise. I have a (..) sort of hobby interest in -

(Interruption by clinical staff 3:34-3:44)

R1: A hobby interest in antimicrobial stewardship so sometimes that was a little at odds from –

(Interruption by clinical staff 3:50 with query for R1 regarding a patient. Recording stopped)

(New recording commenced)

JC: So, you mentioned that you had an interest in antimicrobial stewardship?

R1: Yeah, so there were occasions when we had patients that maybe were intubated but were intubated with (.) something that wasn’t necessarily, particularly lung pathology related (..) had a TAC that had some results of reasonable (.) kind of intermittent (intermediate) Ct values that weren’t particularly low that ended up on antibiotics for a longer period of time than perhaps they would have done previously. It’s difficult to say (.) a lot of patients on PICU end up on antibiotics for a longer period of time than they might do (on a ward) because they’re critically unwell and I understand that. So (.) it’s difficult to say how much that information played into that, but that was something I noticed during the study.

JC: And you mentioned as well, you compared this test to the standard viral respiratory panels. Can you tell me a bit more about that?

R1: In terms of? (..) So (.) some of the patients have a BioFire I think is the fairly standard viral panel that we send and (..) again it gives the same information, but the TAC gives more information in terms of bacteria that it finds as well. I have had previous experience in both this hospital and another hospital using a viral panel to try and identify viruses. This is the first time I’ve had a kind of (..) RNA type sampling for bacteria in respiratory panels.

JC: Okay (..) so you’ve alluded to this a little bit. Can you tell me about any situations in which TAC was problematic for you?

R1: So, there were a couple of patients who attended with non-respiratory (..) well (.) were intubated and ventilated for non-respiratory reasons so I think at least one of them was a child with status epilepticus who had a complex genetic background, but the preceding symptoms were non-infective. It was a patient who had known epilepsy that was in status epilepticus, was intubated and ventilated for a period of time and they’d had a couple of desaturations over night (..) for which some secretions came up the tube. A TAC was requested and came back with a respiratory bacteria that was there (.) present with a medium Ct value as it were, and that patient was (..) I believe either commenced or continued on antibiotics for a seven-day course whereas your pre-test probability of this was a respiratory issue was pretty low all things considered. So that was an issue that I had with those particular patients, was that perhaps that these tests were ordered perhaps without necessarily thinking about where the pathology might be. Which is, I guess, is an issue with all testing everywhere really. But those are certainly a couple of patients and a couple of times with the TAC that it came at odds with what I felt which was perhaps the right way to use the test.

JC: So, are there any circumstances, if you had TAC routinely available, where you would request it for a particular indication?

R1: So, yeah, so I mean, a lot of the pathology that comes through intensive care is respiratory driven. Especially in winter, it’s the primary driver of what brings kids to PICU. Standard microbiological testing of

[5:00]

respiratory tract secretions is pretty poor at identifying pathogens. so, if I had a child that came in with a primary lung pathology that seemed to be infective in nature then I think most of the time most of the time these kids will have been started on antibiotics and most of the time we won’t grow anything of great use from a microbiological standpoint with standard microbiological testing. In that case, TAC can be really useful in terms of narrowing the spectrum of our antibiotics if we find something that is present and significant, as well as if there isn’t anything significant there, potentially deescalating if there is a viral pathogen that is plausible for causing this particular type of illness but de-escalating antimicrobial therapy when necessary. But yes, it has great uses when the pre-test probability of a lower respiratory tract infection is high.

JC: You gave me some sense of this, but how confident were you in interpreting TAC results?

R1: So, I was confident in where the testing (.) where the trial told me what was significant or what was considered non-significant (..) as a closet control freak it was not something I’ve looked at myself so in terms of my confidence in saying yes, I think this is a significant result and no I don’t think this is a significant result, not hugely confident. So, within the trial parameters I was able (.) clearly these were the Ct values that were cut-offs and were shown as to why that was the case. I did do a little bit of digging because I’m very sad like that and obviously this seems to be adult data from adult ITU if I’m correct that looked at the Ct values and what they sort of considered significant. But as newish technology I think that is something that is probably in the (..) the data’s still not necessarily all there. That’s a personal opinion and I could well be wrong but that seemed to be my confidence as to where that seemed to be where it was.

JC: Mm, and did your confidence in interpreting TAC results change from the start to the end of the study?

R1: Yeah, I think so because, like with most things that happen in PICU I tend to go and read about it and actually reading about the technology and reading about where the Ct values had originated from that this was something that was backed by at least some data from the adult world rather than just relatively a arbitrary cut-off where we thought significance lay. So that made me feel more confident in interpreting results in the extremes of Ct values. Obviously, there’s a spectrum and there’s a grey wash area in the middle which is more difficult to interpret. Certainly, looking at the data myself made me feel more confident about interpreting the data as the study went on.

JC: Okay, and did you interpret results on your own or did you rely on other people?

R1: I mainly relied on other people, as in, I looked at the results myself but the nature of PICU and the fact that we have microbiology ward rounds relatively regularly throughout the week meant that I wouldn’t make decisions based on the TAC results (..) I wouldn’t base the choices of my therapies based on the TAC results on my own, it was certainly in consultation with microbiology or the PICU consultant.

JC: Mm, and how did you use TAC alongside existing diagnostic tests?

R1 :(laughs) Um, it often depended on who was on. So, some consultants were more comfortable ...

[10:00]

R1: with looking at the results that were given by TAC and coming to the conclusion that this was likely a commensal organism that actually was not necessarily pathogenic in this particular case (.) that the clinical course and their experience was very much a (.) either non-bacterial or not infective in nature, whilst the result may have shown significance it didn’t alter the therapies that we were on. Other consultants were less happy with that and were more (.) would more readily continue antimicrobial therapy or broaden antimicrobial therapy based on the results themselves. So, it depended really who was in charge (laughs) to be honest with you.

JC: And were there any factors relating to the way samples were collected that you felt may have impacted on the test?

R1: Um, hhhh, the honest answer is I didn’t see very many samples being taken. So, it would probably be wrong of me to make any assumptions based on how they were taken, it was requested then happened. I did occasionally see people walking around with facemasks which I assume was part of it, but I didn’t see very many being taken so I probably couldn’t comment on that.

JC: Okay, so overall would you say TAC is a reliable diagnostic test?

R1: Yes, when factored in with every other diagnostic test which is to assess its pre-test probably and assess the results of it in that framework, but yes, I think it is a reliable test when used correctly.

JC: This is the last, specific, question. Would you recommend that TAC is routinely embedded into clinical practice?

R1: In intensive care yes, I would. It is a very useful medium and the more we are exposed to its information the more readily we will be able to interpret it properly and utilise it properly. In intensive care setting I think it has great value.

JC: Yes, and I noticed you mentioned intensive care specifically, so why do you highlight that?

R1: So, the reason is because I have a pre-existing bugbear about the sampling of respiratory secretions in terms of in the general paediatric side of things. Things like cough swabs, thinks like nasal swabs. You’re swabbing a non-sterile site, you’re gonna get bacteria, it’s gonna be there. The most sterile area you’re going to get is from an intubated and ventilated patient from deep respiratory secretions. That’s always been microbiological gold standard so I see no reason as to why it shouldn’t be the genetic, DNA gold standard as well. It has great value in intensive care. In the general paediatric population, my worry is that the data will be poorly implemented and poorly, what’s the word I’m trying to say, poorly interpreted and would lead to significantly more widespread use of antibiotics which is something I’m fervently against.

JC: Alright, and [R1} are there any other thoughts you would like to share?

R1: No, I don’t think so, this has been very cathartic. (laughs)

Date: 08/02/2022

Interview with: C1, PICU consultant

Interview duration: 12:28

Interview location: Consultant office

JC: To start off can you just say your name and what your role is in PICU

C1: My name is [C1] I’m a consultant in PICU at Cambridge University Hospitals NHS FT.

JC: Thank you [C1] can you tell me overall, what was your experience of using TAC in PICU?

C1: It was very good; I mean I think it was a very positive thing. It seemed to be useful in decision making (..) it was sometimes useful in titrating antibiotics and also useful in terms of kind of virology information available. Just in terms of patients being isolated and so on, like that.

JC: So, you’ve started to touch a bit on this already, can you tell me a bit about how you used TAC to manage patients specifically?

C1: How I used it to manage them?

JC: Mm

C1: I mean I suppose when children come into PICU with acute illness we often think of sepsis or infection or viral infection as likely culprits, as possible causes, so having the facility to make a molecular diagnosis, when we’re concerned the focus is in the chest, very quickly is extremely helpful. Particularly with respiratory infection in paediatrics, it’s quite unusual to get a positive bacteriological diagnosis so being able to have the information about the bacteriology despite perhaps a negative culture was incredibly helpful in understanding what particular antimicrobial therapy, if any, the patient should be on. So, I would say that the benefit in terms of diagnosis and management of bacterial infection was really helpful. It was also helpful in relation to viral respiratory infections but perhaps not quite so much as that information is available from other sources. I mean we still have the facility to do nasopharyngeal aspirates for virology in children and during the project there was another technology introduced by the hospital, the BioFire system. Which allows us to make similar a similar array of virological diagnoses as TAC. So, it was helpful in terms of viral infection, but I think the real benefit was in the diagnosis and management of bacterial infection.

JC: Okay, that’s great. The next question is, where there any situations in which TAC was helpful (.) you’ve answered that a bit so is there anything you want to add to that?

C1: Well, I mean I can give an example.

JC: Sure.

C1: We had a child who came in who was a teenager who was ventilated, who had radiological features of ARDS with 4 quadrant airway shadowing, and we didn’t have a diagnosis and the TAC came up as *Chlamydia psittaci*. So, we were able to make a diagnosis of psittacosis. Which is something I’ve never seen before in a child. And the investigation prompted us to go back and as the family if there was a parrot at home, and there was. So, it all became clear the parrot had died, and I’m sure you remember the case [JC]. So, it became clear that this was something that we needed to treat, and that was the cause. Now (?) we’d have got that information (..) well possibly at all (.) without TAC and I think that was incredibly useful. So, I suppose that the most prominent and obvious example in my mind, but there are many examples in children whom you know the microbiological diagnosis wasn’t clear and the investigation helped us to titrate antibiotic therapy properly, so we were treating the bug causing the problem. Or, if there were no bugs, to stop antibiotics in a timely manner.

JC: Okay, thanks [C1] so then looking at this from the other side, were there situations where TAC was problematic?

C1: Yeah, so I suppose the problem is that, you know, because you’re looking at an investigation which has, as far as I’m aware, not been clinically -

[5:00]

Validated it’s not totally clear how to do that. We’re sort of finding our way, particularly in relation to the Ct number and what is meaningful and what is not meaningful, and I mean I know we discussed cut-offs and having our own microbiology department involved and that was helpful. But I suppose I am still not entirely clear in my own mind how sensitive and specific those cut-offs are and what they really mean. Having said that it seemed to be, you know, as a clinician the numbers that we’ve picked seem to work clinically. But I suppose that’s the problem when you have a very sensitive investigation relying on PCR sometimes things flag up as positive when perhaps they’re not they’re just bystanders rather than causing disease.

JC: Mm, and so if TAC was routinely available outside of the study which situations would you use TAC in?

C1: I would use it in any child admitted to PICU who is ventilated in whom there is a possibility that the child has got a viral or bacterial lower respiratory tract infection.

JC: Okay, that’s great. So, the next part is seeing how people have understood the test. So, the first one is, can you describe how you interpret a TAC?

C1: How I interpret it?

JC: Mm

C1: I ask you (laughs). [Note that the research team did not make treatment recommendations but provided clinicians with a reminder of how the test worked when requested]. Well, obviously we look at which bugs have flagged up as positive, then I have a look at the Ct value and if the Ct value is over 30, I tend to discount it. If it’s lower than 30 than I worry it may be significant. If its below 25 or 20 I’m fairly convinced it is significant.

JC: Okay, how confident were you in interpreting the TAC results?

C1: At the beginning, not very confident but I think I became more confident as time went by.

JC: Okay that answered my next question which is good. And you’ve sort of eluded to this as well. Did you interpret the results alone or did you rely on others to interpret the test?

C1: I think it depended on when the results came back. If the results came back late in the day or perhaps when there wasn’t any microbiology advice readily available, then (?) I was interpreting them myself. On the other hand, if there was microbiology advice readily available or the microbiologists were doing their round, we would discuss it with them. (?) we would discuss it with them.

JC: Okay, and how did you use TAC alongside existing diagnostic methods, like your cultures, virology, biochemistry results (.) all of that?

C1: I mean I don’t think we stopped sending any of the other routine cultures. I have a feeling, I don’t have numbers, I think that we did less NPAs during the study period than before. We didn’t rely on it really at all in relation to diagnosis of COVID-19 because there are other ways of looking at that although it was useful to correlate the findings with the other investigations that were being done for COVID-19. So, yeah, I don’t think we stopped doing anything else during the study so I think all the other investigations continued to be done other than, I wonder, and I don’t have data for this, but I wonder if we did a few less NPAs than we might have beforehand.

JC: So then, this last section is more about what you did when the results were back. Did you change patient management based on TAC results?

C1: Yes.

JC: Okay, were there any factors relating the way samples were collected that you feel had an impact on the performance of the test?

C1: Well, I wasn’t directly involved in the sampling. So, I can’t really comment on that other than to say my assumption, which may be incorrect, is that the sampling was reasonable, so I tended to not concern myself too much with sampling. I mean I think if it’s a PCR test, everything is amplified massively -

[10:00]

C1: so, I don’t have a feeling for how sampling technique may have impacted on results. Though clearly that may be an issue.

JC: Okay, although you weren’t involved in doing the sampling yourself, are there any things you think of that might have impacted the performance of the test?

C1: Sampling wise.

JC: Mm

C1: Well, yeah, obviously the sampling protocol wasn’t followed appropriately that would impact on the results of the test.

JC: Yeah, can you be more specific though? What factors? Are there some that might have had greater impact than others in the protocol?

C1: Well, not getting a proper specimen, failing to suction deep enough, you know, failure to put in the required amount of saline. All that kind of thing.

JC: Okay, so overall do you feel that TAC is a reliable test?

C1: Yes.

JC: Okay, so if you were to rate the test from 0 being unreliable to 10 being highly reliable, where would you put it on that scale?

C1: Well, it depends on what the questions you’re asking is. Cause I think as a rule in, I would say very reliable, I would say 8 or 9. As a rule out I’m not so sure. But I don’t have data so I’d probably (?) and give it an 8 or 9. But I don’t have – That is kind just the feeling of a jobbing clinician rather than based on data.

JC: Okay, so would you recommend that TAC is implemented into routine clinical practice?

C1: Yes.

JC: Okay, in which circumstances and settings?

C1: Well as I said, I think ventilated children in whom there is a clinical concern that the child’s got lower respiratory tract infection either viral or bacterial.

JC: Okay, and is there anything else you wanted to comment on?

C1: No.

Date: 10/02/2022

Interview with: R2, R3

Interview duration: 14.31

Interview location: PICU doctor’s office

JC: Firstly, can you tell me your name and what your role is on PICU

R2: I’m [R2]

R3: [R3] PICU/PaNDR Fellow

JC: Can you tell me about your experience of using TAC on PICU?

R3: Do you mean the results?

JC: So firstly, can you talk about times you requested TAC on the unit?

R3: I’ve requested them for new admissions or put it in the notes and asked the nurses to do it for me.

JC: Okay, and in what circumstances?

R3: For intubated patients mainly. For intubated patients who are newly arrived, and it seems like a respiratory infection and that’s when we’ll tend to do TAC.

JC: And what was your opinion on the use of TAC in the PICU?

R3: I thought it was very good because I am of the impression that it seems to be very established, and the microbiology staff seem very accepting of it. It seemed to be a useful tool to guide treatment. I’m unsure about whether this was replacing our usual respiratory array or something different.

(Interruption by clinical staff 1:34)

JC: Can you describe how you use TAC in managing your patients in PICU?

R3: We use it in place of a standard respiratory viral PCR. That was my understanding, we used it to guide treatment, and expectant management you know.

R2: So, I think the TAC is useful because it has the Ct values, so if it is…

(Interruption by clinical staff 2:04)

R2: Okay so in regard to the Ct values, my understanding is that if it is less than 30 then it is significant. I think previously when you have an array of, like, candida and whatever it’s just helpful for you to sort of put weight on what’s important and what’s not important and also help you treat with the relevant antibiotics.

JC: Can you think of any more specific situations with the test was helpful?

R2: It’s difficult cause there have been quite a number of patients that have come in and I think it has guided, for example, if someone is on ceftriaxone or something like that, then you’ll change it to co-amoxiclav or something like that if there’s anaerobes in there. So, it has guided our use of antibiotics and obviously we know about antibiotic resistance so that's relevant in that sort of setting. (…) but I can't tell you specifically, about a specific patient. But it certainly helps us discontinue certain antibiotics, especially if there are some growing things…more viruses, then you can safely stop the antibiotics as well.

R3: I would agree with that. Yeah, I don’t have anything to add really.

JC: Okay and then on the other hand were there any situations where TAC was problematic?

R3: Not in terms of patient management that I can think of.

R2: I think obviously we all use our clinical judgement when you're reviewing a patient and you’re looking at a patient as whole. You’re looking at their other counts, your white cell count, your CRP, and if you’re quite concerned about secondary infection despite them having a viral infection then I think you are going to say you might continue antibiotics. So yes, it guides, but you’re not removing your clinical hat.

JC: And in what circumstances, if there are any at all, would you consider requesting TAC if it were routinely available outside the study?

R3: Yeah, it seems to be better than the standard respiratory microarray that we do. Presumably, like [R2] was saying, because it’s quantitative and the fact that it's probably more (..)because it gives you a wider range of results.

[5:00]

JC: So, the question is, more about, what times would you actually ask for it?

R2: What time did you actually ask for it? I suppose if you're worried about a VAP, you can use it couldn’t you? Or deterioration, or they’re not responding to treatment…

R3: Definitely (..) I mean certainly the intubated patients who are quite unwell and you’re suspecting VAP that will certainly guide your treatment. Obviously, somebody on optiflow or whatever it’s not better for that. But certainly, I think it’s useful.

JC: And you've touched on this a bit, but can you describe to me how you interpret a TAC?

R3: So as [R2] was saying - positive, negative, then if it’s positive the Ct value. It’s like the equivalent of a titre, isn’t it, presumably. A strong Ct value is a -

R2: We also discussions with micro, so we are lucky to have micro ward round twice a week. So, as well as having that TAC we do have guidance from the micro team [and can ask] is this relevant or not? Should we be treating? Even if the titre is a bit low and you have a zoo (laughs) in there and then you’ve got a patient that’s recovering and looking well you have to think is it relevant if the patient’s doing really well and maybe determining the duration of antibiotics. So yeah, guidance from the micro team is useful.

JC: Okay, and how confident were you in interpreting the results?

R2: I was looking at the Ct values to be honest. I’d look at the Ct values and say if it’s less than 30, you know, if it’s in the teens you thought it was relevant. And we would discuss with the consultant. That is a cop out isn’t it, but -

R3: Yeah, I was going to say the same thing. Having the Ct values gives you a higher degree of confidence rather than a positive/negative, where you think positive for rhinovirus and enterovirus, great, what are you going to do?

JC: Alright, and did you feel like from when you were first introduced to the study to the end did your confidence change at all with interpreting the test?

R3: No, I think because I was slightly confused as to why we were doing a Biofire and a TAC and a standard respiratory array. And everyone picks and choses what they use or I’m not sure what’s standard practice, so it was a bit unclear. That doesn’t really answer the question.

R2: So, I would say, I think that familiarity with the study is …obviously initially you think what is this Ct value but as time goes on you feel a bit more comfortable with it and understanding that it’s a BAL, you know you’re getting it from the lungs. So, it seems more relevant than the sputum sample where you can get contaminants and things like that. So sometimes you can compare what the TAC shows to the Biofire, which is more viral. And, also to the respiratory array and see the difference, whether you have any differences with the result. The sensitivity of the results as well.

JC: Did you interpret results on your own or rely on others to interpret the test

R2: Rely on others (laughs). So, you could make some suggestions but certainly confirmed , received support from others, I should say.

R3: I would agree with that

R2: (laughs)

JC: And you’re also touched on this a little bit as well, but how did you use the results of the TAC alongside diagnostics methods that we’d normally use on PICU?

R3: Yeah, so it’s like [R2] says you correlate it with the results of the other tests wouldn’t you. And then you interpret it within that context.

R3: I would agree.

[10:00]

JC: This is the last section, and this is putting the test in context. Did you change patient management based on the results?

R2: I can definitely remember a few times where we asked the question ‘should we stop antibiotics’? and then we said in conjunction with other results, and the TAC has shown, again (..) or the repeat TAC has been negative, or it has just shown picornavirus, then let’s stop ceftriaxone. So, in that respect it has.

JC: Were there any factors relating to the way samples were collected that you had a feel an impact on the performance of the test?

R3: I never saw the samples collected.

R2: So, I think you’re quite reliant…so we would request them, and you don’t know how it’s being done or, you know, whether it’s done well or not.

R3: This was a micro-BAL.

JC: Yes, that’s correct.

R3: I don’t even know what that means. I know what a BAL is. I don’t know what a micro-BAL is.

R2: (laughs)

R3: Do you know what a micro-BAL is?

R2: I suspect that they’re going down and using the suction probe to go down.

R3: But you put a bit of saline in do you?

R2: Yeah, put saline and –

JC: Yeah, so we would put down the in-line suction catheter that’s already been primed with saline and you put it down to the level of the carina. And we flush 1ml/kg of saline up to 10ml maximum and then –

R3: 10mL?

JC: Up to 10mL if they were a 10kg or more child. And then we aspirate it back up again straight away into a sputum trap.

R3: That’s interesting, so why am I getting so worried about saline bagging with 1ml..2mL when you’re putting 10mL down? Interesting, sorry.

JC: On the whole would you say it’s a reliable test?

R2: So, I suppose I don’t know in regard to sensitivity and specificity how specific it is. But it seems it was more reliable, but I don’t know whether that’s more reliable than the other test. I don’t know what your results show. But that’s the impression that I was getting. Certainly, the consultants seem to like the TAC.

R3: They loved the TAC.

R2: Yeah, they loved the TAC in comparison. And I understand the adult intensive care are using it, so they have some experience from there and maybe that’s why we are trying to adopt it in a paediatric population.

JC: So, would you recommend that the test is embedded into routine clinical practice or not?

R3: I don’t know is it TAC versus Biofire, is that what’s going on? (laughs) I’m not sure. Having that quantitative thing is really helpful, isn’t it? I would recommend it.

R2: I think it’s almost like everything isn’t it. When you have a pilot, and then you’ve got used to it, and now you ask us these questions. You’ve got us thinking perhaps we should be looking closely as to comparators. I’m not necessarily comparing all the three tests; I just have an impression of how people are using it. So, I think if we have to really quantitatively look, I suppose that’s what you’re doing in your study, you know then we would pay more attention to how it's being taken because I don’t know how it’s being taken. And pay more attention to making sure that how we are looking (..) how we are changing our practice. So, I think that saying recommend (..)it seems good but because it’s a pilot, if it came around again, we would pay more attention to it. Apologies, we were paying attention (laughs).

JC: Is there anything else you wanted to share?

R3: No, I don’t think so. Thank you.

Date: 11/02/2022

Interview with: N1

Interview duration: 04:56

Interview location: PICU

JC: So, what’s your name and role

N1: My name is [N1] and I’m a senior charge nurse on paediatric intensive care.

JC: First question, can you tell me about your experience of using TAC on PICU.

N1: Yes, I’ve only done that as part of the RASCAL trial and my experience of it was when we were first asked to put in the saline first for taking the samples, I was slightly nervous, because I thought it was a large volume. I did it about five times I think on the trial and during that time, every time I put the fluid down and I took it out it was difficult to measure how much I removed but I didn’t see any adverse effects. So, once I’d done it those first couple of times, I was happy with how much fluid I was putting in.

JC: Okay, that’s good to know. So, then we took that fluid and did a TAC test on it. What did you think of that test itself?

N1: That I wasn’t aware of really because the doctors were involved in looking at the TACs. And I was aware that some of the consultants were more interested in it than others, and I don’t know if that was just because they were interested in respiratory or they were interested in the research. So then were more interested in the respiratory. I was told that occasionally it picked up things that you never would have known about. It occasionally picked up some rare things. But then there was other things it was picking up and the doctors said oh that’s (..) just you get that for everybody. Some of the tests were worth doing, but it was almost as if they were looking for numbers and unless it picked up what they were looking for…well ah, yeah, yeah that’s a red herring.

JC: Okay, that makes sense. Can you describe if you used, as part of the PICU team, TAC in the management of patients?

N1: I think it was done on the PICU in the management as in that sometimes it directed antibiotics, but I think a lot of the antibiotics that we give anyway are quite broad spectrum and so then because it was being so specific it didn’t then necessarily change the antibiotic because they would have already been on those antibiotics which they used for broad cover usage.

JC: Can you think of any times where the test was helpful?

N1: Not me as a nurse, it wasn’t helpful. But I think it might have directed the doctors, but as a nurse, it never came to my management of the patient.

JC: And can you think of any times where the test was problematic?

N1: Only when it would throw, as the doctors would say, that it was throwing up red herrings and we’re not going to treat it. Well, what’s the point of testing for something, you’re now getting the result, but you’re just saying, yeah, but everyone has that result.

JC: And if you could (…) well you can order tests as a PICU nurse, in which situations would use it in?

N1: I think I would use it (..) if I was allowed to order that test, I think I would use it for, I think at the moment because everyone is being tested for COVID, everyone is being tested for RSV, I think I would use it perhaps if it were one of those patients that didn’t come back on the routine, oh it’s not COVID, it’s not RSV (..) I might use it then. But then I might find out it’s bocavirus, and then I would think hang on a minute what’s bocavirus? You’ve invented a new virus I’ve never heard about. I think I would do other tests first.

JC: This section is on interpretation of the test. I know that you said the doctors were doing it a bit, so let me know how this goes. Can you tell me how you interpret a TAC test?

N1: No.

JC: No problem, that’s fine. Do you interpret results on your own or rely on others?

N1: I always relied on others to interpret the results.

JC: And who was interpreting the tests?

N1: It was usually done on the ward round, and again it was certain consultants that seemed to have an interest in it so they would then look at the TAC in a bit more detail. So, they would then perhaps interpret those results and act on them accordingly.

JC: And how did you use TAC alongside other diagnostic methods on the unit?

N1: They would bring it up on Epic, so as part of Epic they could see it on there as well and so that was where that was used.

[interrupted by clinical staff]

Date: 14/02/2022

Interview with: C2

Interview duration: 11:05

Interview location: Consultants office

JC: Can you tell me what your name is and what your job is on the PICU?

C2: Hi my name is [C2] and I’m one of the PICU consultants here.

JC: Thanks [C2] so first of all, can you tell me overall what your experience of using TAC on the PICU?

C2: It was quite good actually, we had results turn over quite fast and we were able to make a decision based on it. Most of the time [inaudible] viruses or bacteria need for antibiotics, stuff like that. It was an added advantage I would say.

JC: How did you use it for your management on the unit?

C2: It was mainly a clinical decision. Most of the patients would come to us with some kind of antibiotics on. You would see, mainly during winter, we get a lot of bronchiolitis patients, and the RASCAL study would give us an answer quite quickly. Whether it’s a virus or whether there is any concurrent bacterial infection, it would give us information on the timing of antibiotics, whether it’s needed or not. And instead of waiting for the culture we could just stop it right away. (…) You could stop it within a day.

JC: You’ve given a bit of an example, but can you name some situations where you found the test helpful in particular

C2: Mainly bronchiolitis, I’ve already told. The history is typical most of the time, during winter, we know most of the time it is bronchiolitis but sometimes, especially in very young infants, they present with apnoea and we’re not quite sure whether there’s anything bacterial, if there’s any meningitis or something like that. They would be on triple antibiotic cover as you would expect. If there was RSV positive on such patients, we would be more confident in stopping antibiotics, not waiting until the cultures are back. If RSV is positive, we could stop acyclovir and ceftriaxone.

JC: Are there any times where you’ve found the test problematic?

C2: Probably, chronic lung patients, sometimes. We would get normal commensals as well. Or (..) with insignificant Ct values. Clinically we know this is not possible to have *stenotrophomonas* or something like that, but it would still come up on the RASCAL study. That would sometimes confuse the clinical picture, I mean confuse the treating team. But at that point we would actually check with a microbiologist and decide if it’s really needed or whether this could really fit in the clinical picture.

JC: That’s one of my other questions. If you felt you were able to interpret the test yourself or if you relied on other people to help you with the interpretation.

C2: So, most of the time we did it on our own. But again, as we have microbiology backup, we would just run it by them as a second opinion rather than as primary decision maker they would be our second opinion. They do come twice a week; we would often ask them rather than just making a decision on our own.

JC: If you were able to request a TAC outside of the study, in routine practice, would times would you ask for one?

C2: Sorry, I didn’t get the question.

JC: If you could routinely obtain a TAC what situations would you want to ask for the test?

C2: Mainly respiratory pathologies – bronchiolitis, or pneumonia and stuff like that. Not necessarily on every septic patient. Mainly those who present with respiratory symptoms, this could give us a lot of information quickly. Rather than waiting for longer. Mainly respiratory pathology patients.

JC: The next part is, how do you interpret a TAC? Can you give me your understanding of how to do that?

[5:00]

C2: First of all, I would see if this fits into the clinical scenario. See if it’s a respiratory patient or say bronchiolitis, or if the TAC shows something similar like RSV or rhino or something (..) a viral picture. Clinically if it’s fitting, I would take it as this even though Ct values are on the higher side, not necessarily the cut-off of thirty. If not, say, most often what we have seen is somebody would be positive for staph, I mean, *staph epidermidis* or say candida, we would go clinically. If that doesn’t fit the clinical picture, we wouldn’t treat it unnecessarily.

JC: How confident did you feel in interpreting the results?

C2: Pretty good actually, we had clearcut guidelines given to us by the research team. A Ct value of 30 or less it’s significant. More than 35 is not significant. Again, if it fits clinically you would treat.

JC: Did you feel that your confidence changed from the beginning of the study to the end in any way?

C2: As we continued to practice, I think it made us more confident. There was no dilemma or confusion in making our decision actually significant or not. Over the course, most of us felt it is quite clear and we were confident by the end, I think.

JC: How did you use the TAC results alongside diagnostic tests that we already use in clinical practice?

C2: Most of the answers to the questions are similar, I guess. Mainly clinical, if the presentation is in keeping with the assay report we would treat it otherwise, probably not really, we would rely on our usual cultures. I would like to make a point here, I mean, sometimes if the clinical suspicion is higher for a pneumonia or something bacterial if the array doesn’t show any bacterial organisms we would wait for the culture, rather than just ignoring the result. You would still continue the antibiotic at that point. It is an adjunct to current practice rather than stand-alone evidence for infection. Depending on the clinical scenario. If someone is in sepsis or in shock, we would not ignore it. Mainly because of (..) respiratory microarray showed a respiratory virus we wouldn’t stop treatment with antibiotics.

JC: Were there any factors related to the way samples were collected that you think may have impacted on the TAC?

C2: You mean, not the deep BAL sample?

JC: Yes, the way that was done.

C2: I’m not sure. I mean we don’t deal with this. Say oncology patients also we didn’t exactly do the deep BAL to rule out fungal but if the clinical suspicion is higher, we would do it. I don’t think that has made any big difference; the way samples were collected. Because most of the clinical scenarios doesn’t need deep BAL in our unit. If there is any suspicion of fungus or anything we would definitely go for a deep BAL otherwise the way, we are collecting samples would show up. If it is positive on the samples which were collected it is positive on the deep BAL.

JC: When you say deep BAL, what do you mean.

C2: Through the actual bronchoscopy. A formal one. So here it’s blind isn’t it, so we’re not sure how deep we are and where the sample is coming from.

JC: Overall would you say it is a reliable test?

C2: From what I observed over the course, I think it is even, results which are (..) I could say I would rely on these microarray results.

JC: Final question, do you think that TAC should be embedded into clinical practice routinely?

C2: Yes, I think if everything, if it’s economically feasible and we are getting results quite quickly it’s definitely worth it. That’s what I feel. If the research is showing it’s helpful then yes. We’re kind of used to it now, getting the results quickly.

JC: Is there anything else you wanted to add [C2]?

C2: Nothing in particular. It was quite helpful, useful for us to get the results quickly.

Date: 16/02/2022

Interview with: N2

Interview duration: 7:05

Interview location: PaNDR clinical offices

JC: Can you tell me your name and what your job is in PICU.

N2: [N2] I’m one of the senior sisters.

JC: [N2], Can you tell me what your experience was of using TAC on the PICU?

N2: For any of our ventilated children with respiratory illnesses we did a bronchial aspirate soon after admission and sent it off for microarray, or whatever it’s called.

JC: Did you find using this test changed management for any of the patients in PICU?

N2: Because the respiratory PCRs always took so long, we were getting results back quicker at that point with RASCALS. The Biofires are now quicker so that’s slightly better. Because our PCRs would take 48hrs this would generally come back quicker. So that was quite useful because we knew what bug they had. I don’t know that it particularly changed what we were doing on the unit.

JC: Were there any times where you found the test was helpful on the unit?

N2: It was helpful that you could, yeah, you would often got which bugs, which respiratory illnesses they came in with and things. There were so many tests and quite often they came up with quite a few bits on there and it was hard to know how many of them were relevant at that point.

JC: I think you’ve started to touch on this, but were there times where the test was problematic?

N2: So, just, I think, because you were looking at that Ct value, weren’t you to see if it was likely that had a high viral load, so it would pick up anything. I think, particularly towards the end it became, a bit more, on the ward rounds that people took less notice of it because they were a bit like ‘oh yeah it shows up stuff but the kids aren’t actually symptomatic with those it doesn’t matter’, so I think sometimes when there was something that was relevant it got missed because people had become so blasé about the fact that there were so many positives being picked up on it.

JC: Outside of the study, if you could routinely order this test. What situations do you think you might order this test?

N2: I think for any of the ones who come in with respiratory symptoms which ends up causing them to be ventilated. We’re now in a better position because the Biofire during daytime hours comes back with four hours and shows most of the respiratory viruses. So, I think it may be less used than it would have done prior to the Biofire because our PCRs would be 2-3 days before they come back often. But yeah, for your bronch’s and pneumonias and flus and things like that it would be useful because it picks up those things.

JC: You mentioned the viruses, are you aware of anything else that is on the array?

N2: So strep’, pseudomonas, I can’t think of what else is on there now.

JC: No problem. Can you talk me through how you would interpret one of the arrays?

N2: I can’t remember which way the Ct score…it’s the Ct score isn’t it? If the Ct score, is it the higher the number, the more likely there is to be a viral load? Or the lower number? I can’t remember which way around it is now. If you were looking, I think if your Ct score is greater than 30, they thought it had a viral load and they were symptomatic and probably shedding. Less than that I think it was that they thought it was a potential, like the covid result, you can sometimes shed for 90 days but you’re not infectious. I may be the wrong way around with the numbers.

JC: And how confident were you in interpreting the test?

N2: So, when I’d look at it on Epic, yeah, fine.

JC: Did you feel that your confidence changed from the start of the study to the end?

N2: Yeah, I think so. I don’t think we ever really thought about Ct values and things like that before on PICU, so I think it was just they were either positive or negative for something so yeah.

JC: Did you interpret results on your own or did you rely on other people within our immediate PICU team or broader team?

N2: So, we would look at it on our own, but we would generally be looked at on the ward round. And yeah, I think was it at 1-30 it was more likely to be viral load? Yeah, pass.

JC: You mentioned the Biofire, but there might be other examples as well, how did you use this test alongside other test we’ve already got within the trust?

N2: So, we do SAMBA COVID swabbing obviously and that comes back quickly and that has a huge impact on where we place patients and what PPE we have to wear so that usually comes back within 2-4 hours. And we will often do the Biofire now, I think it has 30-40 viruses on it, quite a lot of viruses on it, and we used to do the respiratory PCRs. The frustrating thing with them would be that we would send an NPA and often the children would nearly get out of PICU before the result came back so it wasn’t a quick thing. It’s useful probably, probably particularly useful in children like the RSV positive bronch’s, because actually once you’ve got a clinical history of bronch’ and then you’ve got a positive RSV often these children are on ceftriaxone and aciclovir and you can stop that because you know what’s causing them to be sick. If it’s taking 3 days for it to come back, it’s a pain.

[5:00]

JC: Did you change management based on TAC results?

N2: I don’t know actually. I guess if you were getting a result back earlier rather than (..) when we were first doing TACs we didn’t have the Biofire, so I guess if you’re getting a result back earlier which then confirms why the child is ventilated, yes sometimes they’d stop antibiotics earlier or maybe I guess with strep’ and things like that leave them on for longer.

JC: Were there any things related to the way samples were collected that you think might have impacted on how well the test performed?

N2: No, I think it took a while for us to get used to doing a deep bronchial aspirate, but I think actually once it was set up and we knew what we were doing with the saline and 3 way tap I think we got much better at doing it. And I think actually now the specimens we take for other studies are probably better because of having to do the RASCAL study cause we all got more confident with doing a proper bronchial aspirate.

JC: Can you think of parts of it that you did get used to or members of the team got used to that made the sampling improve?

N2: I think just getting used to the setup with your 3 way tap and then knowing when to put your saline in and when to suction and making sure you’ve got a really good sample.

JC: Overall do you think this is a reliable test?

N2: Yes

JC: Finally, would you recommend this test become available as part of routine practice?

N2: I think it’s useful to have it there as an option to do, I don’t think we should be doing it on everyone. But I think it’s useful to have it there as an option to do it.

Date: 24/02/2022

Interview with: N3, R4

Interview duration: 17:54

Interview location: PICU doctor’s office

JC: Can you say your name and what your job was in PICU during the study

N3: I’m [N3] I’m a band 6 on PICU and PaNDR.

R4: I’m [R4] I’m one of the PICU registrars.

JC: Okay, what was your experience of using TAC on the PICU?

R4: I think from a clinical point of view it’s really, actually really useful. I think it was helpful for us to identify whether there was a causative organism first. In terms of understanding the pathology it helped us to stop antibiotics at times where they may not be needed. It was a broad assay, so we got quite a lot of information back.

N3: From a nurse perspective it was more to do with obtaining the sample for the TAC. For a start, while we got used to the idea of how to do it was quite fiddly, and also the idea of putting 5mL of saline straight down into the lungs, especially if you had a sicker patient could be a bit ‘ooh, do I really want to do this or not?’ but obviously it wasn’t just our decision alone. We would be talking to the doctors and saying, ‘are you happy going ahead on this patient or should we wait a bit longer before we do it’? but obviously it’s quite a handy diagnostic tool as well. So, they’re often quite keen for it. And also, we’d coincide it maybe with their physio session, if not, to get the best result.

JC: And [N3] you’ve mention that being a new thing to be doing on the unit. Did you notice any change with the nursing team from the start to the end of the study in doing the sampling?

N3: I think towards the end of the study everyone had become much more confident with the process and how to do it and obviously how your patient was likely to cope with doing the TAC. Most of the time the patients were absolutely fine it didn’t really affect them at all. Actually, it was quite a good way of getting some secretion clearance.

JC: Were there any times you can think of where you had trouble getting samples or where it caused problems with patients?

N3: In terms of difficulty, in terms of obtaining a sample it might be someone with secretions at that particular time weren’t particularly loose or they have a few mild respiratory symptoms but there probably wasn’t much going on. but it’s like ‘oh perhaps something’s wrong with the patient so let’s try it and see’. So sometimes that might be a bit trickier to obtain a sample so we might try but if we didn’t get anything, we might then think about, ‘oh physio will be in in a couple of hours we might try with physio once we’ve got a bit more up’ or something. Or maybe we just need to wait another day for things to move a bit more. In term of difficulty, it might be a patient where it would affect the patient if they were in quite a lot of oxygen or a lot of PEEP you might be a bit wary, thinking is it actually an appropriate time to do the TAC right now, or not. But I haven’t known a patient where I have personally done it where they have desat’ed or anything. But I’m sure it has happened.

JC: So, picking up on that, are there any factors you think about the way we took samples that might impact on how well the test worked?

N3: In what sense do you…?

JC: Any things related to the procedure of taking the sample where you think actually, that might have meant the test might have worked better or not worked as well.

N3: Not that I can think of. Because I think, certainly, saline does aid, the sample. Even if, before, if you think about the alternative was doing an NPA, you know, you do your sample dry and you would always run your catheter through a bit of saline anyway, so I guess it’s just that but going further into the lungs. Sometimes you don’t think you’ve got anything, but you look, and you can see it.

JC: Anything else [R4]?

R4: I guess what you’re saying [N3], thinking about doing it at the right time in term so the natural history of the pathology for that particular patient. I guess making sure the samples you’re getting up will be affected by the volume of fluid we’re using. If they are particularly dry, if it’s safe to give them more.

JC: Are there any situations in particularly that come to mind where you think it was particularly helpful for a patient?

N3: I would say particularly given the fact we’ve just gone through COVID in the last year but even the year, purely on the basis of, a lot of testing stopped for a lot for things. Actually, having the TAC had given more answers than we normally would have got. There weren’t at one point even running [tests] for your basic *rhinoviruses*, the normal winter bugs you would get so actually the TAC gave us answers that weren’t available to us at that point.

[5:00]

R4: I agree, on the clinical application which is giving us information which sets on the right course in terms of understanding what the underlying driver of the child’s illness is and targeting of therapies towards it which might mean stopping things that are unnecessary a lot of the time. Or thinking more broadly about what’s needed. I think that was what worked well. From my experience the number of times where children have been started on antibiotics and we were going through our normal respiratory support management and then someone would come in and say have we done the TAC and be proactively asking for it. I suppose from experience we’ve had, examples of results that come back that have changed the way we do things. I’m struggling to be more specific than that.

JC: That’s okay.

N3: I guess previously they might have looked at doing a BAL, if they needed a bit more of a clear out but equally if they needed a better sample actually. The TAC is somewhere in between doing that extreme to just doing a normal sample.

JC: You mean a bronchoscopy?

N3: Yes, a bronchoscopy.

JC: Yes sure, were there any times where you got the TAC result, and it caused you problems?

R4: The Ct values are useful. Sometimes when they’re borderline it’s difficult to know what to do with that result. I think that’s just a part of becoming experienced with the test and sort of like all of these things, tying up these results you see with the clinical picture. But yeah, if you get a borderline Ct value, you’re like, is this significant, is it not? Should we be treating it or should we not? I think that can be tricky, especially when you have potentially different clinicians who have a different view on that. Yeah, it’s always nice to have a yes/no answer (laughs). That grey zone is always a bit difficult.

N3: I guess from the nursing perspective it would often throw up an infection control issue. The sample might come up positive for something we weren’t expecting and all of a sudden, we’re like ‘oh they’re in the middle of the bay, we might need to put them in a cubicle’, or ‘they’re okay to be here while they’re in closed circuit ventilation but once extubated we might need to isolate’ or vice versa. That would be the main one for us.

JC: So, let’s say the study is finished. If you wanted to order this test for any kind of patient what sort of circumstances, if there are any, would you order the test?

N3: I don’t know I assume I would expect from an ITU point of view it would become part of our admission process. Normally when a ventilated patient comes in you do an ETT sample of secretions as well as an NPA or something. But it may well be that TAC would become part of that. It would be routine probably for respiratory viruses at least, an expectation.

R4: I think it’s the same. I guess we’ve got to choose wisely because it’s annoying getting a result that you have to deal with when actually it’s a bit of a distraction from the clinical picture. If there was a child with a primary respiratory complaint who was intubated and ventilated. And I think that sort of (..) is it going to change the clinical course? I think we have to be expecting they’re intubated and ventilated for a period of time for it to make sense really. I guess you could argue that if they’ve got a respiratory complaint whether or not they remain intubated and ventilated it would be good to know what the [unclear audio] are.

JC: Okay. For these patients where you’ve got the BAL sample, you’ve got the result back. How did you interpret that result?

N3: I guess mostly it’s interpreted by the medical team, I guess it’s just looking at that alongside the clinical picture of the patient. Does it make sense? Does it fit? Is it likely to be a contaminant or not? Probably not, given the way it’s done but actually does it fit what is actually going in with the patient.

R4: Yeah, I think it’s tricky. You could sample well people and see what you get up from that. But I think from our point of view the reason they’ve been selected in the first place is because they’ve got a respiratory issue and so then I would be inclined if they had a significant Ct value to take it at face value, that’s the cause of the problem most of the time. Yeah, I don’t know.

[10:00]

JC: And what do you mean by significant value?

R4: Was it (..) 30 was the threshold? Is that what (..)? I would defer to your (..) it’s essentially the number of rounds of amplification you need to get a positive value, right? The more rounds you go through the less likely it is to be a significant sample. The lower the number the more significant. I don’t know where the thresholds come from and how valid they are, I’d be interested to know a little bit more about that, but I think the rule of thumb was less than 30 was likely to be significant and more than 30 may not be, but exactly where you draw that line is something that’ s sort of been decided (..) beyond what I understand.

JC: How confident were you in interpreting the results when they came back?

N3: I don’t know because I wasn’t aware of all that about the different rounds or what not but on my level as a nurse we just see if it’s positive or negative. It’s more then you guys are looking at how genuine a result it is, you know.

R4: I do think you’ve got to be a little bit careful. It might be a reflection of how the information is presented in Epic because it could be really easy to see something and say it’s positive and therefore it’s significant without going down and looking whether it is or not. So initially I didn’t really understand you know, the full set of information that we were being given and what it meant. But, you know, after a couple of weeks or whatever a few of these being put in front of you I think it’s easy enough. But I guess if there was a way to make that slightly more idiot proof it would probably be good. But sometimes it’s useful to actually see the Ct count because there’s the subtle (..) it might be what is significant for one person might not be significant for somebody else.

JC: My next question is did you feel your confidence in interpreting the test change throughout the study.

R4: I think so, yeah.

JC: Did you interpret results on your own or did you get the help of others to do that?

N3: Definitely the help of others, it would be the help of others, the medical team.

R4: I think mostly by myself. But as far as handing over a positive result I think I would usually explain why I thought it was positive, so ‘it’s come back positive and the Ct count is’.

JC: [N3] you mentioned how we used, depending on the pandemic, NPAs and things like that on the unit. How did you use the results from TAC in the context of us doing all of these other tests in PICU?

N3: Well, I guess the TAC took the place of, normally every patient that comes into PICU, if they’re ventilated, particularly respiratory, you would do a ETT aspirate and NPA. TAC took the place of the routine ETT sample which meant you were able to obtain a better sample I guess because you know you’re putting saline down with it. You’re more likely to get something up initially. To do that I guess it was looked at alongside that. I guess we still did do an NPA as well. But yeah, they would often (..) if the TAC hadn’t been done the doctors would often come around in the morning and be, like, can you do a TAC? It would be interesting to know, I think as well, because it was coming back quicker than some of the other samples at times as well.

R4: I think similarly, I don’t think it replaces a respiratory culture, so we continued to do those, NPAs, sometimes I didn’t appreciate the extent of the number of things that you get back on TAC. For example, I think I remember there was one apnoeic young child where we said ‘oh could this be Pertussis’? We ended up doing an upper nasal or NPA swab for Pertussis purely because I didn’t appreciate, that it came back on the TAC.

N3: I guess the only benefit of that would be, from a Public Health England point of view. I don’t know if they would recognise the TAC or not. I assume they would, but if not, that swab would then be recognised if it was positive. Only because the TAC is in a research phase at the moment so I don’t know if they wouldn’t accept it because we do act on the result, but I guess something like that.

R4: There may be instances where a child is genuinely too unstable for us to be (..) we do want to get early samples where an NPA is easy to do, and you might get a result from that.

N3: I think of an example of a baby that was in bed [identifiers removed from transcript]. He was the pulmonary hypertension. He came over and he was quite unwell and we’d proned and were on nitric and a lot of oxygen. I think they discussed a TAC but thought today is not actually a good day to do it we’ll just (..) we want one, but we’ll hold off for a bit longer.

[15:00]

R4: There’s obviously all your non-ventilated children who I think there is still a role for NPAs. In my experience there weren’t instances, even though we were doing multiple samples, there weren’t instances where we had conflicting results come back from my experience. Which I think is quite reassuring. It’s really annoying if you get a positive on one and a positive on something else or a negative. How do you then interpret it?

JC: Last section, did you change patient management based on the results?

N3: It depends on the result, like you said, in terms of management it might be antibiotics or not antibiotics. Or stopping them. Or starting them. I guess if most of them come back with a viral respiratory it wouldn’t change how we managed them it just gave us a definite answer to (..) ‘oh well that’s what’s caused this respiratory collapse’. We’re often looking for *rhinovirus*, RSV, you know any of that so, it’s confirmed our diagnosis you might say and sometimes it might throw up something useful you need to treat.

R4: I think more than anything it helped us to stop antibiotics. I think it did change management. In my experience there weren’t cases where we then ended up having to backtrack and restart antibiotics because something else, because something had been missed.

JC: On the whole do you think it’s a reliable test?

N3: Yeah.

R4: Yeah, I think so yeah. I think we’re, sort of (..) there’s a healthy scepticism of using anything new. I felt that way initially, but it did feel reasonably reliable to me.

JC: And finally, would you recommend that we embed TAC into our routine clinical practice?

N3: Yeah.

R4: Yeah, I don’t know what the costs are in terms of, you know, time and people and all of that sort of stuff but there’s definitely a clinical application for it. It makes sense.

N3: You might just have to make sure it’s not for everyone or something. A separate criteria. There’s probably no point or your traumatic brain injury patient but in wintertime for your respiratory patient or those where you’re not sure what’s going on and you’ve looked at all other avenues, I guess.

JC: Was there anything else you wanted to add?

N3: No

Date: 01/03/2022

Interview with: C3, PICU consultant

Interview duration: 11:52

Interview location: Consultant office

JC: So, the first question is (.) what your name is and what is your job in PICU?

C3: I am [C3], I’m a PICU consultant

JC: Thanks [C3]. So, the first one’s a bit broad. Can you tell me about your experience of using TAC in the PICU?

C3: So, the TAC has been really useful, especially in giving us a quick result and covering lots of different pathogens (.) viruses and bacteria and it has been helpful in trying to decide whether the patient needs specific antibiotics or whether this is a viral infection (..) whether or not the patient is developing VAP. And, yeah, so that has been one of the major advantages of doing it.

JC: Mm, okay. So how did you use the test in management of your patients?

C3: So, I think at times it has been helpful to decide whether we can switch off antibiotics or whether we can (.) give specific antibiotics to cover for specific pathogens. I don’t know if you want me to give an example?

JC: Yes, that would be great.

C3: I can talk about a patient which I was quite heavily involved with. There was a diagnostic dilemma with this child that had nutritional deficiency (..) came in with unexplained fever and multisystem problems post-COVID infection and it was not certain whether this patient had PIMS-TS pathophysiology or not. And, despite having some early improvement, this child was then deteriorating again, and it was unclear as to what the main driver of that deterioration was. He also had significant skin involvement so there was a question mark around translocation and development of secondary sepsis. TAC was extremely helpful as it gave us [the result of] aspergillus which is what the patient had and then we could direct antifungal treatment specifically towards that. And this child had, had, prior to that (..) had had quite a lot of steroids as well as immune suppression because of the multisystem involvement and possibly that’s what drove the aspergillus. So, we would not have picked it up otherwise and it gave us an early result and we could titrate the treatment towards this specific pathogen.

JC: So that’s a really good example of where it’s worked well…are there times where the test you’ve ordered, and it’s been problematic for you?

C3: Yes. Especially in the initial part when we started using TAC because it’s such a highly sensitive test. You’d sometimes get an almost zoo of different pathogens coming through, and it becomes very difficult to interpret that. Over a period of time, we grew a bit more familiar (..) with becoming familiar with and understanding the Ct values and interpreting it alongside the microbiology team when they came on the ward rounds. To understand whether we take this pathogen seriously or whether we say that this is just colonisation or something that the patient has as a carrier state rather than being infected with it. So that still can be challenging at times and um one really has to have experience as well as the clinical oversite of the patient to take it in context…whether or not what we’re seeing is really the reason why the patient is sick….especially for those borderline Ct values when you have them between 25 and 32. You know when you have it (..) although it is below 30 it’s not quite (.) very low and it’s 27 and 28 and whether or not I want to add specific antibiotic to cover that pathogen. It’s something which is difficult at times.

JC: Mm, [C3] you’ve started to touch on it, but can you tell me how you go about reviewing those results. What’s your process?

C3: I think we never take a decision in isolation it is always discussed with the microbiology team. We’re quite lucky here at Addenbrooke’s we have twice a week micro and virology rounds. So, they come on the unit and usually we’ll discuss it at that point. If it seems urgent enough and I can’t fully interpret the results, I can call them out of hours or in hours on the days they don’t come in. So, the interpretation is always done alongside them, and we make a joint decision whether we’re escalating antibiotics or antifungals or

[5:00]

C3: …whether we’re in a situation where we can take them away. I suppose some of the bacterial pathogens that you would find on TAC sometimes when the Ct values are borderline, we might wait to depend on what the patient is doing – their clinical state, their inflammatory markers as well as awaiting culture results. We might withhold escalating the treatment unless the patient is really sick and then we would act on that. Yeah, I suppose it’s never (.) never the test that is interpreted in isolation (..) and never individually by the clinical team - it’s always done with microbiology input.

JC: Mm, and I suppose as well that this is a new test that goes alongside things, we’ve already got…like biochemical markers, like CRP, we’re still doing culture and things like viral swab panels…all of those other investigations (.) so how did you look at this result in context of all of those other things?

C3: I think specifically when you talk about other respiratory viral panels that we were traditionally used to doing I think whilst you were doing TAC, because the results came back much earlier than, sometimes, we would get from the respiratory panel, it was quicker to start interpreting results from that than wait for the respiratory panel to come back. As well as it’s sometimes even good to know there’s no bacteria so you could try and switch of the antibiotics earlier if you identified a virus in the patient. So, I think yeah those are probably the two key areas that we can start looking at and like the example I gave an early result can also help us in escalating treatment.

JC: Okay (.) and (.) if TAC was routinely available outside of any studies are there certain circumstances in your role where you would want to order the test in particular?

C3: Ah (.) yes and I think we’ve grown used to having TAC and the advantage of early results have really outweighed the actual taking the sample and sending it to the lab [for culture] so in fact we’re asking for it more and more. Especially in the child that presents with a respiratory illness and given the current COVID pandemic of course we do get the COVID results back much quicker. In terms of the intensive care planning, it’s useful if we have an early viral result to try to define whether the patient needs to be isolated and that has huge implications for the staffing and whether we can admit or not. And of course, also in terms of deciding the length of antibiotics and if the patients have already been on PICU for a certain length of time and if they’re deteriorating, we can decide whether they need new antibiotics or whether their deterioration is due to something else. These are the situations in which we’re asking for it more routinely now compared to prior to the study happening.

JC: And individually how confident do you feel in interpreting the results when they come back.

C3: Um, it’s difficult to give a percentage to it, but certainly a lot more comfortable than when we started and when you started doing the study. And (.) yeah, you’re kind of very confident in certain Ct values in [saying] this cannot be a pathogenic bacteria or virus…and moving on and trying to define what else there might be. And a little more confidence in discussing it when we’re making a treatment plan with the infectious disease or microbiology team because they can (..) their understanding of the result is much better (..) to question why we are doing something or why we are not doing something, and it is particularly relevant when we are talking about an immunosuppressed patient, specifically the oncology children.

JC: So last section, did you change management based on the TAC results?

C3: A few times yes.

JC: And then, were there any things you can think of in the way that samples were collected that might have impacted on how well the test performed?

C3: Ah, no.

JC: Okay, that’s alright. So overall do you think it is a reliable test?

[10:00]

C3: I would say yes although I am aware that this is not as well evidence based as I would like it to be, because we haven’t done a control sample [of patients] we just used it and I suppose it will be really good to see that happen where we’re doing it in one lot and not doing it in the other lot. I suppose it might be a difficult study to conduct and get a large enough sample size for it to reach significant. Sample size. I suppose within an individual patient we do have some kind of control cause we’re also doing their cultures and we’re doing some of the other established tests to see how they compare with TAC and certainly it’s quite a sensitive test. And again, as I said, the specificity needs to be taken in the context of the patient’s clinical condition and sometimes that’s not the easiest.

JC: Mm, so based on all of this would you recommend that this test is embedded into routine clinical practice.

C3: Yes.

JC: Is there anything else at all you want to add?

C3: Well, I think the team has worked really well with you and [Research nurse A] and [Research nurse B] you’ve been phenomenal in coming over and identifying patients and working with the clinical team and deciding what might be the right time to take the samples, especially when the patient is unstable. And you’re doing all the legwork so congratulations on finishing the study.

JC: Thank you [C3], I’ll stop there.

Date: 03/03/2022

Interview with: R5, N4

Interview duration: 12:34

Interview location: PICU doctor’s office

JC: First can you say your name and what your job is on this unit?

R5: My name is [R5] I’m a PICU and retrieval registrar.

N4: My name is [N4] I am a junior sister on PICU.

JC: Can you tell me your experience of using TAC on the PICU?

N4: So, I mostly did the actually array and collected it. It was pretty easy to follow. There was really handy step-by-step guides and packs already put together, so we could just follow the instructions pretty simply. We did them at the request of the doctors, or medical team.

R5: I guess my input feels less, so I had little input in terms of the actual technical aspect of doing it, but I would be the mugs who would say let’s do a TAC please and then look at the results.

JC: On that then, can you tell me with the TAC results, how you used them for managing your patients on the unit.

N4: From a nursing point of view, I would just keep an eye out for the results and let the doctors know but it didn’t really change our treatment until the doctors had reviewed.

R5: It probably (…) the most helpful thing I think is in a patient you had no other focus or no other bug for, and something flagged up positive all of a sudden, your mindset and your management changes as you’re less worried that you’re missing some horrendous abdominal sepsis and in fact you’ve got a guilty organism. So that was really helpful. And certainly, it seemed to yield more positive (…) results more useful results than a generic NPA or whatever else.

JC: Are there any times for the patients you’ve cared for where it’s been particularly helpful?

N4: Not really from a nursing perspective.

JC: Or any times you’ve relayed the results and you’ve found that something has changed?

N4: We’ve had (..) I remember having one that had quite a few weird and wonderful sporadic ones which, I think it led off to immune system testing because it wasn’t something you’d typically find from what I remember. I’m sure there was one that led off to an immunosuppressed sort of picture. But, yeah, probably.

R5: I don’t think I can remember a specific case where we’ve thought ‘oh this was very good’. It was very helpful. Most of the time it came back with something and was extremely helpful. But I suppose the other side of the coin is when it comes back completely negative. It’s so broad that you’re then more confident to be able to say well, we’re not missing something here. So, it got used both in (..) it was useful as a rule out as well as a rule in, I think.

JC: Are there any times where you got the results back and it was actually problematic?

N4: I think we had one where it was potentially contaminated, there was too much in there and stuff that looked like it was potential contamination, so it wasn’t really beneficial from that point of view because you didn’t know if it’s a true result or not. Probably the only problematic one.

JC: If the Trust had this test routinely available and you could use it any time, which situations would you order the test if there are any?

N4: From a nursing point of view, if they’ve come in with any sort of respiratory symptoms or unknown (..) sepsis, temperature of unknown origin, it would probably be what we would think about from a nursing point of view.

R5: I agree, it’s a nice low intensity, or it’s an easy to do test from my perspective (laughs) (..)

N4: Yeah, it’s non-invasive.

[5:00]

R5: It gets a good admission screen, as I think that’s helpful. That’s the biggest use from my perspective.

N4: It’s no different to when we do respiratory PCRs on admission anyway for those sorts of patients so it’s not really any, yeah, it’s not causing more work, we already have to do aspirates anyway, so it makes sense.

R5: I suppose the other angle, possibly with covid, is if you’ve got a rapid test and you test a patient in ED or on the ward or somewhere not PICU, and they come back that they’ve got you know, five different bugs, your level of concern in terms of discharge planning is going to be higher than if you’re thinking we’ve just got RSV.

JC: So, you mean co-infection [R5]?

R5: Yeah.

JC: So, when you do get those results back, how do you interpret them?

N4: I don’t (laughs).

R5: We sit in handover and people gesticulate at the screen and go ‘oh look we’ve grown this’ would be (…) I don’t think we interpret them particularly in detail, is that the right word? It says what’s positive and you go, well that’s that then.

JC: So, we also had Ct values for each of the targets did you use those at all?

R5: Sometimes, my own experience and knowledge about Ct values is limited but yes, certainly if there was a low Ct value then we would not be as excited as if it was super high. Have I got that the right way around? Other way around.

JC: So how confident were you in interpreting the results?

R5: As a group, confident.

JC: Okay, who would be in the that group?

R5: Consultants.

JC: PICU? Anyone else?

R5: Not normally.

N4: Micro? Would micro come.

R5: Yeah, probably on micro ward round. But...

N4: I think micro look at them, don’t they?

R5: But that’s, what, weekly?

N4: Yeah.

R5: So, more often than not, it would be the PICU team.

JC: And did you feel any more confident as the study went on in being able to interpret results?

R5: It was the same baseline level of confidence.

JC: With the TAC result, how did you use that in the setting of us doing other investigations as well, things like NP swabs for viruses, we can do things like cultures, we can do CRPs, lots of other tests. So how did this fit in with all of that?

N4: I guess if your TAC covers some other stuff, you would replace some of the other screening tools if that one covered other screening. It incorporated it, that’s the right word.

R5: Can you ask the questions again?

JC: In PICU we use lots of different infection diagnostics, things like cultures, pre-existing PCR tests, we use biochemical tests, so how do you use TAC in the setting of all of those other types of investigations?

R5: I think my perspective, probably it’s an adjunct rather than a replacement, but I suspect that comes down to lots of doctors wanting more data points. So, if you’ve got lots more data. You can add data points to your CRP, your cultures, your PCR then you feel like you have a fuller picture. If people said let’s stop doing cultures and CRPs and lets just do a TAC people would push back.

JC: Okay. So, here’s the last bit. Did you change patient management based on results?

N4: Yeah, I think some of the kids were started on different drugs weren’t they.

R5: Yeah.

N4: From what I remember there were a couple that led to further testing of other things, so yeah.

R5: Yeah, I can’t remember a specific case but definitely being able to refine things (..) or further hone your treatment modality I think that definitely happened.

JC: Were there any factors related to the way samples were collected that may have impacted on how well the test performed?

[10:00]

N4: Because there are a lot of different elements, there are a lot of contact points that have the potential to be contaminated, I think if it’s something we’re doing more regularly, if there was a possibility for a pre-set kit, sort of already made, that would be beneficial because there’s less points at risk of being contaminated. And then, when you would turn the tap sometimes, you’d turn it the wrong way and you’d have to start again because you’d suck out the water, you’d turn the suction the wrong way. I think now we’ve used it and we’ve found the potential areas, there are a couple of bits that could be tweaked moving forward but nothing remarkable if that makes sense.

R5: I have nothing to add.

JC: So, on the whole do you think the test is reliable?

N4: Reliable with adequate staff knowledge. I think if you’ve not been show how to do it, then there’s a risk of contamination, but if you’ve got a trained member of staff when you do it and you know for a fact that you’ve not contaminated it then yeah. Again, down to competence.

R5: I think so, and I think the fact that you’ve got the numbers on there as well, assuming there’s adequate staff training…reliability…because then you can discount low level stuff.

JC: Would you recommend that this is something that is embedded into routine clinical practice.

N4/R5: Yes

JC: Is there anything else you wanted to share?

N4: No, I think it was really well done. It was very easy to do and probably one of my favourite ones to do, it was really easy to follow and go along with.

R5: I agree, easily done and helpful datapoints as an outcome that changed care and practice, it was good.

Date: 21/03/2022

Interview with: N5, N6

Interview duration: 16:33

Location: PICU doctor’s office

JC: Can you tell me what your name is and what your job is in this PICU?

N5: My name is [N5] I am a staff nurse on PICU.

JC: What band [N5]?

N5: Band 5.

N6: I’m [N6] and I’m a band 7.

JC. Thank you. First question, can you describe your experience of using TAC on PICU?

N6: I can start. So, mine has been quite limited but I have on minimal occasions helped, or have been helped with you taking a TAC and doing the BAL.

N5: I’m fairly happy with doing the BAL, as you know I have involved the research team on occasions that they are free to do clinical stuff with myself. Generally, once the steps were out and once the sample, the template, of how to do BALs, it was fairly easy to put together.

N6: And I’ve never put it together.

JC: Can you describe if you use the TAC, as in the results of the test, in the management of patients on the unit?

N6: Definitely. We’ve isolated children based on the TAC result. I think in terms of nursing management, yeah, isolation.

N5: Same. And I think once we were told whether to isolate or not then I think the care that we deliver is pretty much the same anyway. Apart from the interventions like isolating or starting so and so medications but generally.

N6: Yeah, I mean, I don’t know if it’s nursing or medical intervention, but if you’ve got a virus and you know you can stop the antibiotics or vice versa if it is a bacterial one then making sure the doctors have seen the result and getting the appropriate medication prescribed.

JC: So, I’m interested in the isolation actually, and what was different when we started using TAC compared to before, about how you would isolate?

N6: A lot of it’s based on hoping it’s just a common virus that we are only able to isolate to a certain degree. We only have 3 cubicles, so I think it helped us make the decision whether to isolate them in a cubicle or not. Or whether we could leave them where they were. Either in a single bay or whether we could cohort them, I guess that would be the other thing. Which is very different from going to the ward because they isolate most viruses.

N5: Or they cohort.

N6: Yeah.

JC: Were there any situations where the TAC was helpful?

N6: The only other thing in terms of if you could actually get a virus, whether it was quicker than getting it the normal way, it’s confirmation for the parents. From a psychological point of view, I think it helps when families are able to identify what has actually cause the problem for their children.

N5: Help them with something to Google (laughs).

JC: What about the other side of things. Were there any times where the test was problematic?

N5: I don’t think it’s the test itself but it’s getting a BAL on an unstable patient. I think it’s tricky for (..) you would look at the skill mix of the people looking after the patient as well because not everyone is either confident to do a BAL or put together pieces or you know, do it on their own, or have someone to do it with them. But I think one of the trickier parts is when it’s sort of, the only test remaining that you need to do, and you know that you have to do it but then this patient is very sensitive to being touched. It’s kind of getting other people and having everyone ready, I’m going to do this, I’m stuck in a cubicle.

N6: I think that would be my side of it, based on that, would be exactly, that I’ve never done it without your help or somebody else’s so I’m less confident doing it therefore I probably wouldn’t do it unless I had someone that knew what they were doing.

[5:00]

JC: Next question, if you could order a TAC for any patient and it was routinely available what times would you want to request one of these tests?

N6: Ideally first thing in the morning.

JC: Sorry I meant what situations?

N5: Probably on admission usually (..) depending on the history that the patient has presented with, obviously if they come in with something and from an oncology background, you’re not going to consider it anyway first hand of course. If it’s more respiratory failure.

N6: I think it would mainly be respiratory. Most other conditions there’s either some other background that would lead you to not think about it. I can’t think of why you would use it apart from respiratory.

N5: If they were probably already admitted in PICU and then new symptoms come up that would indicate an infection or something respiratory in origin then you could probably consider that.

N6: And also, if you got an oncology child that presents with something that (..) the BAL is from right inside the lungs whereas when you have awake patients, you’d have to put them to sleep probably to do it. That might be more helpful, to have a bronchoscopy instead.

JC: Can you describe how you would interpret a TAC?

N5: [Nursing team member] told me how to do it earlier.

N6: I don’t know. It’s been a while since I looked at one. I’m trying to remember what’s on there.

N5: Same. I’ll be honest, I rarely looked at one. I’m more involved with how to do it.

N6: I look at them on the doctors rounds but I’m trying to remember a list of viruses. I think it would have to be in terms of interpretation it might explain why they’re unwell in terms of respiratory but maybe it’s an incidental finding, the viruses. I guess it would depend on why they’ve come in and what their history is. Which is why you have doctors that actually decide whether it’s relevant or not.

N5: I think I speak from a bedside nursing point of view, sometimes you don’t really get a chance to look at the results or you know it’s obviously we have to verify that what we were told is correct. Most of the time people just tell us as well. It’s not obviously always best practice. If you’re running around sometimes you can’t look at the results and interpret them. So, from my point of view, I haven’t looked at (..) I’ve sent a few off but I haven’t really studied in detail if that makes sense.

N6: I think sometimes it’s just confirmation of why they are so unwell. I think that’s the thing. When you grow a specific virus, you often say it explains they do what they do. I think we are all very aware you can get a virus; viruses will cause certain children to behave in a particular way. Sometimes it’s getting the actual virus to say what it actually is. It doesn’t always change treatment sometimes

N5: Or it’s the final straw to make everything make sense. It’s the last bit of information, so sometimes ‘ah okay it makes sense’, to make you go like that.

JC: So how confident were you in interpreting the results?

(…)

JC: Not very?

N5: I haven’t had very much experience with ...

N6: I think it’s more a medical thing. I think it might explain if it’s a virus, why they’re acting (…) but it generally falls down to a medical person to interpret the results.

JC: There are other ways we can diagnose respiratory infections we use often in PICU, like a culture, or sometimes we do NPAs, so how did you use TAC alongside these other things that we already have in place?

[10:00]

N6: I think the TAC is more deep seated, if that makes sense, you’re able to get to places that you can’t get to with just a swab up the nose. Even with trying to get tracheal aspirates there’s not always enough sputum there to get it. The fact you do a lavage and give it a really good washout and a good rinse.

N5: You get the good stuff.

N6: You get to places you wouldn’t normally be able to get to, I think.

N5: And the quality of the specimen, basically, is what you’re getting as well.

JC: Because of the way the samples are obtained, does that change your confidence in the relevance in the relevance of that information. Is that what you’re saying to me?

N6: I don’t think it changes the confidence; I think it probably…

N5: It’s more that the sensitivity of the test.

N6: Maybe, I don’t think it changes your practice but the fact you’ve got a specific virus or organism…

N5: I wouldn’t say more information, but if you get what I mean, it kind of gives you a more detailed (…) information I suppose, if that makes sense?

N6: It’s a bit wishy washy that one (laughs).

JC: Final section, were there any factors relating to the way samples were collected that impacted on how the test performed?

N5: I think, especially from doing BALs, sometimes you’re just not sure if you managed to get enough sample. When you say put 10mL down and say you only get 2mL back, well what happened? Have I gotten the right amount of sample? Am I meant to get all the 10mL or just 5mL back? That sort of thing.

N6: I have literally done one with you. I feel that in this particular case I’m not actually very confident in answering that question.

JC: That’s okay. Do you think it’s a reliable test?

N6: I think generally it has been. I think everything is open to some sort of contamination.

N5: Same.

N6: I think because you are very careful (..) we’re always very careful, but there’s less (..) what was the question again?

JC: Do you think the test is reliable?

N6: I think it’s probably more reliable sometimes than what we do. When you do certain things, if you don’t go far enough you don’t get enough sample, or you might get a very limited amount. Whereas with this because you’re going much deeper you’ve got more fluid to wash it around and suck it out, I think you’ve probably got a better chance of it being more reliable but again there’s always that risk of contamination because you’ve not done something correctly.

N5: And the factor of technique as well, how you obtain the sample as in any test, not just this test. That I guess, that also factors in to how effective the test is and how good the results are (..) good or bad the results are because of technique.

JC: Would you recommend that TAC is embedded into clinical practice?

N6: Certainly, for respiratory I think it would be something that is good. I think doctors clearly are still asking for it

N5: Like today they asked for it

N6: They asked about it today, so I think it is something for which they are quite keen, and I think it probably would be quite a good thing to have. Again, I think, as you say based on the patient stability as it not something that is done lightly. The difference is with doing a swab up the nose or a small tracheal aspirate is much easier, but I think it probably gives you a better range of perhaps the load of the virus, and perhaps getting the actual viruses as opposed to maybe not getting anything.

N5: It would also depend, this is from a nursing and medical communication point of view, it would also depend on the doctor’s awareness as well of the existence, and how much experience the doctor has with the TAC if that makes sense. So, you do see a few, not stereotyping doctors, but you do see doctors who would order this more frequently or would ask for this or some that are just completely, not blasé but don’t really mention it.

N6: I think it was because you were doing a study that was much more highlighted and it was only perhaps the consultants that asked for it, and the registrars because they changed have no idea what you’re talking about. If it were something that was embedded it would become standard practice and I think people’s techniques would be better, nurses would be more confident.

N5: We had a point that we didn’t really have that many ventilated patients. Or ventilated patients but not necessarily respiratory in nature so they wouldn’t require TAC and obviously for at least more than half of the staff here wouldn’t have done it throughout the duration of the study, which is not their fault, it’s just we didn’t really have those patients to begin with.

N6: We had the wrong clientele.

JC: Is there anything else you wanted to add?

N6: It will just be interesting to see the results when it’s published, because I think it would perhaps highlight (…) we know what you have been doing but whether it was done well (..) whether it was worth doing and something that would be taken forward. I guess like everything, how it is interpreted.

Date: 28/03/2022

Interview with: C4

Interview duration: 16:42

Interview location: Consultants office

JC: Can you tell me what your name and is and what you do on the PICU?

C4: My name is [C4] and I’m a consultant in paediatric intensive care.

JC: Thanks [C4] can you tell me about your experience of using TAC on our PICU?

C4: Using the TAC over many months now, I think that going into it there was a fair amount of interest in using it. There was certainly quite a lot of excitement around it and to suddenly have something which would give us fairly quick and definitive results. We did get quite a lot of information and learning about it and stuff like that. So initially that was very exciting. I think that we’ve all really, kind of, changed the way we practice in many ways. It has certainly added a lot to the diagnostic process, and it has certainly made a difference in terms of management. We’ve found it quick; we’ve found it reliable. I guess the thing is I’m sure we’ll miss it when it goes, or when it changes. It’s been very good.

JC: Can you describe if you used it in the management of your patients?

C4: As I said before, going into it, to suddenly have a new diagnostic test was quite a big deal. What we’ve been doing now (..) it’s really become part of normal practice and part of regular practice. We’ve been pretty much initiating it as soon as a child with respiratory failure comes in. It’s certainly become part of the admission discussion, if not, the initial investigation. It’s something that’s very quickly mentioned and conducted at the soonest possible opportunity. And certainty part of our daily routine, means that everyone’s asking about the results and we’re asking about how it may change management. Which certainly has had an impact on (…) but you might come onto that slightly later, I guess. So certainly, it’s become a normal part of practice.

JC: Can you give me some examples, more specifically, where it has been helpful?

C4: I think in a general sense it’s certainly adding to diagnostic information we can get. I mean the vast majority of children, as you know, that come into PICU, will come into PICU with single organ disease. And they come in with respiratory failure, the majority of those will have a viral aetiology. We have pretty good testing for those. But what we’re finding more and more is that we’re getting patients with more complicated histories, certainly more patients who have multiple hospital stays, multiple other problems and you’re looking for organisms outside *rhinovirus*, *RSV*, and all of that sort of thing. So certainly, it’s making a difference for those sorts of patients. So certainly, with other conditions, for example, now that we’re dealing with COVID and we’re dealing with children who are on immunomodulation, for example, certainly looking for bacterial foci, looking for atypical foci, has become definitely a bigger part of our work for sure.

JC: And can you tell me any times where the test was problematic for you?

C4: Yeah, so, one of the things that we’ve found, as with any test, it’s all well and good doing the test but we have to interpret the results. We have to interpret the results in context with the patient we’re looking after. Part of that is obviously taking the clinical information and adding the diagnostic test to either confirm or deny what we’ve found. Rather than looking for something new that’s suddenly going to change our management. So, I think the biggest challenge we’ve had is dealing with the sensitivity of the test. Because we are getting a lot more positive results than we did on culture. The challenge is putting that into context.

[5:00]

Otherwise, taking what you know from a clinical point of view. Taking your experience. Adding that onto the clinical scenario you’re dealing with. Then interpreting that in a way that’s hopefully going to have an effect on patient care. The second part of that is using appropriate antimicrobials and using the result to do that. And again, that’s a challenge. Some of the time it's relatively straightforward, but I said I can’t say for certain but subjectively we do need more advice from microbiology about that. Certainly, in the context of PICU I think we probably talking to them more about the significance of certain results which (..) probably (..) we did more than we did in the past. We’re all working together; I don’t think it’s a big issue. We’re pretty well supported so I think that’s not a huge issue but certainly there’s probably an additional element of screening that you need to do of a positive result to say it’s positive, sure, but is it actually positive in the context of the patient?

JC: Now the core of the RASCAL study is finished, TAC is now available, what circumstances, if there are any, will you be requesting a TAC?

C4: That’s on a spectrum. I think, to some degree. TAC has been so useful to us (..) there’s a school of thought that said it would become a default test almost. I know that’s probably a dangerous thing to say. I think that still there will certainly be some screening. There will still be use for instances where you have a single organ respiratory failure and if you don’t get *RSV* back, if you don’t get a viral aetiology very quickly. I can see already that TAC has become a routine part of PICU life and a routine part of PICU testing. Just because of the ease of doing it, the speed at which it comes back, in its value in tailoring antimicrobial care. Also, its ability to help us to stop antimicrobial care. I think all those things, like many things it may become a victim of its own success in a way. It may become a routine part of testing. You know, we have to be careful of that. I think we probably have to ration it’s use and make it cost effective and not just do it for everybody. Certainly, it’s become part and parcel of PICU care I would say over the last two years.

JC: So, you send your TAC off, you get a report on Epic. When you’re looking at that report how do you interpret it?

C4: I think that it’s extremely important that it’s taken in context. Obviously, it’s important to know the patient’s history, all the other factors we look at on the ICU. How they’re ventilating, their x-rays, all the other bits of looking at their inflammatory screen and all that sort of stuff. But I think it will be just one other tool, used in the context of all the other tools we had. With the old analogy of the jigsaw, it will be once more piece to add to it. Is it a very useful piece? Yeah, and it’s quite a big piece as well. Certainly, I think that having extra information particularly when you do have diagnostic dilemmas and diagnostic uncertainty then it’s certainly going to be really useful.

JC: I guess, getting in depth, looking at that more in depth. When you look at the report how do you interpret that?

C4: When we do have a positive result, if we get an organism that is in context with the patient illness then of course it makes it much more certain that’s a causative organism. The issue we did raise slightly before is when that’s not in context which is either an organism you’re not expecting, or it’s detected at a relatively low level, so you think is that colonisation? Is it really a thing? But I think that’s not a new decision, it’s not a new process. I think almost every diagnostic test we have; we have to make that decision. Information is power, the more information you have I think the better decisions you make. So, the interpretation, as you interpret more tests you get better at doing it.

[10:00]

If you looked at people doing the test two years ago you would probably have a very different idea of how people interpret it. I think our interpretation of it is getting better.

JC: That’s one of my questions, has your confidence in interpreting this test changed throughout the study?

C4: For sure, for sure it has. As you get to know the test, as you get to know the information it gives you. If you understand the context in which we’re getting it, both positive and negative, as you know we’ve had many positive outcomes, but we have had outcomes which have left us wondering the value of the test. But I think you have to put all those together and decide what you use it for (..) the useful thing is that you have a collective knowledge and on top of that you have the collective knowledge of everyone interpreting the test. You have a big clinical team, all interpreting the test, you have input from microbiology. There’s a process by which you improve your decision making over time. For sure. I think that the end product of that is that people are fairly confident in their ability to interpret the test. We don’t often sit there scratching our head thinking what does this all mean? I think that’s the bottom line.

JC: We spoke a little about your management based on TAC results, but in a pragmatic sense, did you actually change things like antibiotics and –

C4: Yeah, I think that we do, and I think that over time we’ve really come to rely on it quite a lot. I think that over time it has become a really important part of the diagnostic process as much as a culture I would say. It’s at that level. I think that’s made our decision making better. It think it’s made it easier, and I think we are putting quite a lot of weight on the TAC results, for sure.

JC: A slightly different consideration, were there any factors relating to the way samples were collected on patients that you think might have impacted on how well the test performed?

C4: With any direct sample you need a good sample. It is an invasive process to get the sample there’s no doubt. And there’s no doubt that there are situations where people questioned whether you should take the sample or not. I invariably think it’s worth the effort and it’s worth the inherent risk because the information it gives is so good and so useful to us that, you know, I think it far outweighs the (..)one, the issue about getting a good sample. Of course, it’s important that the majority of the samples are taken by well-trained people who make sure they get good samples. I think that the diagnostic yield has been good enough to say the samples you get are good. I guess the thing I don’t know is, we are micromanaging the quality of the samples. That’s almost impossible to do. But I guess the follow on to that is that the samples (..) whatever we get …the quality of the samples we can rely on. It’s not an uncommon question to say, ‘well should we be taking it on a patient that’s extremely critically unwell’? or extremely unstable and my answer to that is its almost invariably it’s worth the risk to get.

JC: Overall do you think it’s a reliable test.

C4: Yes. If I broke that down into how you decide whether a test is reliable. I think that the results that we get for patients are reflected by the context of the illness of the patient. I think we get results quickly. I think that they almost always correlated with the clinical picture we’re looking at. Slowly over this period of time we’ve become very reliant on the TAC results. I think we invariably find them very useful. We invariably find them helpful; we’re often starting antimicrobials but also taking them away. I think it’s been very important for us.

JC: Would you recommend that the test is embedded into routine clinical practice?

[15:00]

C4: I think that there’s many ways we look at whether they’re suitable for that or not. I think for us I would certainly say yes. However, I’m sure there are people at some stage will look at its cost effectiveness, it’s diagnostic yield, the difference it being a diagnostic test or a screening test. Whether it fills the criteria for both. It almost has become a default already. Like many things which inherently are safe and reliable and give you quick results by stealth they sometimes become the norm. Whether you like it or not. Or sometimes without evidence. And it’s very difficult to unpick that and turn the wheel (..) the clock back. But I think it’s a sign of it’s success in how much of a norm it’s become to be honest. It’s not an unusual situation to hear a TAC to become part of a discussion on the ward round. There’s probably not a ward round that goes by now without mentioning TAC. Which I guess is a sign of success.

JC: Is there anything else you want to add as a final comment?

No, I think that as a unit we want to support innovation. We want to support new diagnostic tests. I think that we were very keen to support it in the beginning and I think pretty much we did everything we could to support it, so I hope that we’ve done that. I’m hoping that the diagnostic yield will be good, and it will make a difference in patient care. I think it has.

Date: 08/04/2022

Interview with: C5

Interview duration: 12:01

Interview location: Consultants office

JC: Can you tell me what your name is and what job you do on PICU?

C5: My name is doctor [C5] and I’m a PICU consultant.

JC: Thanks [C5] The first question is can you tell me about your experience of using the TAC on PICU?

C5: So, I found it very useful in trying to identify any pathogens earlier than usual, which would then affect my decision as to whether to either have a narrow spectrum antibiotic, continue with broad spectrum, or discontinue antibiotics altogether.

JC: You alluded to this already, but how did you use it in managing your patients?

C5: More or less like I said, there were cases where we weren’t sure whether the origin was bacterial or viral in origin, so TAC was helpful in helping us decide that way. It affected antibiotic initiation or stoppage, and equally it would affect narrowing down the spectrum of antibiotics if we had a result earlier that suggested we could use something narrow spectrum. So, practicing good microbiology-based decision making.

JC: Were there any times where you found it particularly helpful, any patient cases or scenarios?

C5: I think in patients on (..) it’s tricky to say because the bulk the admissions on PICU is with respiratory causes and that’s where we’ve used it most of the time. So obviously respiratory cause where the main beneficiaries but especially during the pandemic viral illness were probably the main reason for admission. So, I would say those with a viral illness where we could stop antibiotics early, were the more frequent beneficiaries of the TAC.

JC: Were there any times when you got those test results back and actually it caused you a problem as a consultant, about how you used that information?

C5: Not really because you never base your decision purely on TAC. You go by the overall clinical picture, so it was almost always a useful addendum to that overall clinical picture. I would never rely my decision purely on the TAC or any single test as a principle. It hasn’t really given me a headache per se. I found it very useful.

JC: Now the study is done, say you’re the PICU consultant on, are there any situations, if there are any, where you would want to order a TAC?

C5: Yes, especially I would like it in most respiratory cases as early as possible I would say. Because obviously these patients come on a broad-spectrum cephalosporin plus or minus sometimes clarithromycin if they’re suspecting atypical. Allowing us to stop those earlier is a long-term gain really in reducing, in our region, resistance to those antibiotics. It’s obviously beneficial to the patient directly as well. Similarly, if in cases of a more chronic child, someone being on the unit for longer and developing an early suspected VAP TAC would allow us to detect that earlier. So that more directly and immediate gain to that population so definitely need that repeat TAC in those patients.

JC: When you order the TAC test you get a result back, tell me about your process of interpreting those results. What did you do?

C5: I think we would see what would flag up, we would see the titre, the degree of positivity if you want, we would then decide if it was significant or not. In context of the titre count and the clinical picture.

[5:00]

We would then base our decision accordingly.

JC: What about in situations where (..) there were for some organisms multiple targets for the same pathogen, so how did you deal with that?

C5: Can you clarify the question a bit more?

JC: As an example, *strep pneumoniae* for instance had two targets on the TAC. Sometimes they might not necessarily have been both positive, it might be one or the other -

C5: I suspect those were the tricky ones. I think that’s where the expertise of you guys running the study was useful and also the discussion on the micro round to understand their significance essentially. But essentially you take everything in the broader picture, of the clinical picture. Because if that flagged up out of left field when you were not suspecting it then you were less likely to act on it. Whereas if we were actively looking for something that would cause a sudden deterioration, we would be more likely to act on it. But I think those sort of grey areas we’ve not used TAC enough or long enough, in clinical medicine, within PICU, to be able to make a standalone decision without discussing them with the experts so to speak.

JC: On that point, how confident did you feel interpreting the results, yourself, firstly?

C5: I’ve used TAC before on research, I was reasonably well versed in it but not within the context of micro or virology. It was more genetic research. In this context the more we used it the more confident we became. It took me time to get accustomed to it.

JC: TAC, you mentioned you were using as an adjunct test for diagnosing respiratory infection. How did you use the information from the TAC alongside those other diagnostic modalities that you’ve got available to you in routine practice?

C5: Like I said the most important part of the TAC (..) it would help me stop antibiotics early, narrow them down early or diagnose VAP early. So, early admissions versus late onset of a new infection or pre-existing admission to PICU. So that, in conjunction with any other broader microbiological studies or inflammatory markers or persistence of fever would be an extra sort of safeguard that we are making the right clinical choice whether it would be escalation or de-escalation. I never used it as a standalone feature. It was a nice luxury to have that. It tells you you’re probably doing the right thing starting or stopping or narrowing down.

JC: The next part is, did you change patient management based on TAC results?

C5: Yes, so it feeds into what I’ve said earlier.

JC: Were there any factors relating to the way samples were collected may have impacted on how the test performed?

C5: That’s a harder question to answer. I’ve always felt if it was a proper BAL sample done by physiotherapists then the yield would be higher. Rather than if the sample was done by myself or a PICU nurse. That’s all I would say. I’ve not seen (…) because the samples were collected by a multitude of different professionals, but you need consistency as to how they are collected and how deep they are collected.

JC: If I can pick that apart a bit, what do you think is different about the way physiotherapy do it compared to some other staff?

C5: It’s more aggressive and you’re more likely to get a larger and deeper sample.

[10:00]

JC: What do you mean by aggressive? Is it that they are doing chest manoeuvres, or is it the breaths –

Yeah, Chest manoeuvres, cough assist, if it’s bigger breaths, more confident with higher PEEPs, it looks like they’re doing CRP basically(..) almost. Whereas PICU nurses are probably more gentle.

JC: That’s really good feedback to have. So, on the whole do you think it’s a reliable test?

C5: Yes, and it will carry on being increasingly more reliable more confident and familiar we become with the grey areas we alluded to earlier. Those are the key ones that need the experts to hold my hand a bit more with.

JC: Is this a test that you would recommend that we bring into routine clinical practice in PICU?

C5: I think it would be useful to, in order to practice accurate medicine, rather than broader strokes medicine. So, it would be beneficial to the patients for sure. It needs to be taken with a broader context of cost in a publicly funded system, however. If money was no object, absolutely yes. If it costs a lot to make those decisions slightly more accurate, then maybe not. But the science of it is very useful and if I could use it long term I would if it’s affordable for sure.
